# Insights into the Global Transcriptome Response of *Lentinula edodes* Mycelia during Aging

**DOI:** 10.3390/jof9030379

**Published:** 2023-03-20

**Authors:** Qi Gao, Yangyang Fan, Sai Wei, Shuang Song, Yuan Guo, Shouxian Wang, Yu Liu, Dong Yan

**Affiliations:** 1 Beijing Engineering Research Center for Edible Mushroom, Institute of Plant Protection, Beijing Academy of Agriculture and Forestry Sciences, 9 Shuguang Garden Zhonglu, Haidian District, Beijing 100097, China; 2 College of Plant Science and Technology, Beijing University of Agriculture, 7 Beinong Road, Changping District, Beijing 102208, China

**Keywords:** *Shiitake mushroom*, mycelial senescence, WGCNA, autophagy, mitophagy

## Abstract

The spawn of *Lentinula edodes* and other basidiomycete fungi tend to age with long-term culture. This causes heavy yield losses if aging spawn is used for propagation. In this study, we cultivated dikaryotic *L. edodes* mycelia in plates for 60 days to produce intrinsic aging phenotypes. We found that intracellular reactive oxygen species levels increased in contrast to mitochondrial depolarization and also observed greater DNA fragmentation with longer culture time. Transcriptome analysis of mycelia at different growth stages revealed pronounced expression differences between short- and long-term cultures. In particular, “phenylalanine, tyrosine, and tryptophan biosynthesis”, “mitophagy and autophagy”, “MAPK signaling pathway”, and “ABC transporter” were among the enriched terms in the mycelial aging process. Weighted correlation network analysis identified *LeAtg8*, *LeHog1*, *LePbs2*, and *LemTOR* as key genes during aging. Western blotting confirmed that LeATG8 and phosphorylated LeHOG1 protein levels were significantly upregulated in aging mycelia. Our combined analytical approach provides insights into the mechanisms that regulate mycelial aging, indicating that autophagy/mitophagy plays a major role in counteracting the effects of age on mycelial growth development.

## 1. Introduction

*Lentinula edodes* (shiitake) is the second most-consumed mushroom globally. In Asia, *L. edodes* is widely used as a healthy dietary and medicinal ingredient because of its unique taste and flavor, high carbohydrate/fiber content, and low calorie content [1]. It has also attracted considerable attention owing to its biological properties, such as anti-tumor, anti-viral, anti-microbial, immunomodulatory, and cholesterol-regulating activities [2]. Given the ease of its artificial cultivation, *L. edodes* has been a focus of rural economic development, typical recycle-economy, as well as sustainable agriculture and forestry in China.

The mushroom spawn is essentially a mycelium “seed” used for mushroom production. It is with spawn that mushroom growers inoculate their growth substrates. After inoculation of a sawdust substrate with spawn, the mycelium colonizes and produces fruiting bodies. Therefore, spawn quality plays an important role in mushroom production [3]. With the development of the *L. edodes* industry, spawn aging has emerged as a major factor that limits production yield. Mycelial aging manifests as a physiological degradation process under long-term culture or nutrient depletion. However, there is a lack of systematic research on the aging mechanism of long-term cultured spawn.

In filamentous fungi, *Podospora* anserina has been extensively studied as a model organism to unravel the molecular pathways involved in the control of biological aging [4]. When *P. anserina* turn to senescence, the mycelia will reduce the growth rate and change their morphology. Characteristic features of aging *P. anserina* include a decrease in mycelium growth rate, reduced aerial hyphae formation, increased pigmentation, and programmed cell death (PCD) [4]. A complex network of interacting pathways, including those governing mtDNA instability, mtDNA repair, the generation and scavenging of reactive oxygen species (ROS), copper metabolism, proteostasis, mitochondrial dynamics, autophagy, mitophagy, and apoptosis, emerged as being of relevance to aging in *P. anserina* [5,6]. ROS are essential signaling molecules, and increased mitochondrial levels of ROS cause molecular damage, which induces aging [7,8]. In decreasing mitochondrial levels of ROS, superoxide dismutases (SOD) are key players which convert superoxide into hydrogen peroxide, along with the catalases and peroxidases, which enzymatically catalyze the further degradation of peroxides into harmless substances [5]. If damage passes critical limits, the impaired molecules as well as those present in excess are degraded by different forms of autophagy [8]. According to transcriptome analysis of *P. anserina*, autophagy takes over quality-control functions from the ubiquitin proteasome system in the control of aging [9]. Additionally, dissipation of mitochondrial membrane potential releases apoptogens, which leads to the execution of cell death.

The features of *L. edodes* mycelial aging, including pigment accumulation and decrease in nuclear numbers, which has been suggested to be associated with apoptosis and autophagic PCD, have been explored [10,11]. However, the mechanisms underlying these aging-associated phenomena remain to be explored. In the present study, we aimed to elucidate the physiological and biochemical mechanisms involved in *L. edodes* mycelial aging. By systematically analyzing colony phenotypes, subcellular structural changes, ROS contents, and antioxidant enzyme activities in mycelia, we determined the aging degrees of *L. edodes*. Transcriptome sequencing and weighted correlation network analysis (WGCNA) revealed that autophagy is involved in the aging process of *L. edodes*. Further, autophagy-related protein 8 (LeATG8) and phosphorylated high osmolarity glycerol 1 protein (LeHOG1) are significantly related to the aging of *L. edodes* mycelia. The present findings provide a foundation for the development of accurate and early detection technologies for aging mycelia, which is of particular importance for *L. edodes* cultivation throughout China.

## 2. Materials and Methods

### 2.1. L. edodes Strain Culture and Sample Preparation

The dikaryotic *L. edodes* strain JZB2102217, which is isolated from the fruiting body of the widely cultivated mushroom variety 0912, is preserved in the Beijing edible fungi germplasm resource library. Mycelia were inoculated on potato dextrose agar (PDA, 20 mL per 9 cm plate) overlaid with cellophane and cultured at 22 °C for 7, 12, 23, 38, and 60 days. The entire mycelia were collected at different timepoints, flash-frozen in liquid nitrogen, and stored at −80 °C for transcriptome and protein assays. The tips or upper mycelia were carefully removed with tweezers, rinsed with phosphate-buffered saline, and immediately used for microscopic observation.

### 2.2. Microstructure Observation and Antioxidant Activity

The ROS contents in mycelia were detected using 2,7-Dichlorodi-hydrofluorescein diacetate (DCFH-DA). Fresh *L. edodes* mycelia under different treatments were washed with PBS buffer three times and incubated with 10 µM DCFH-DA (Beyotime Biotechnology, Beijing, China) for 30 min at 25 °C in the dark. Staining was observed under an IX71 inverted fluorescence microscope (Olympus, Tokyo, Japan). The total protein contents of *L. edodes* mycelia were extracted using a protein extraction kit (Bestbio, Nanjing, China). The total protein concentrations were determined using a BCA protein assay kit (Beyotime). SOD activity was measured using a kit (Nanjing Jiancheng Bioengineering Institute, Nanjing, China).

The mitochondrial probe 5,5′,6,6′-tetrachloro-1,1′,3,3′-tetraethylbenzimidazolyl-carbocyanine iodide (JC-1, Beyotime, Beijing, China) was used to measure mitochondrial membrane potential (MMP). *L. edodes* mycelia were treated with 10 μM JC-1 in the dark for 30 min and washed twice with JC-1 buffer. Green and red fluorescence were observed under the same conditions using an IX71 inverted fluorescence microscope (Olympus, Tokyo, Japan). The intensities of red and green fluorescence in each cell were analyzed using ImageJ software. The MMP of hyphal cells was determined as the ratio of red to green fluorescence. In total, 100 cells were observed per sample. 4′,6-diamidino-2-phenylindole (DAPI, Sigma-Aldrich, St. Louis, MO, USA) and terminal deoxynucleotidyl transferase dUTP nick-end labeling (TUNEL) fluorescence staining (Beyotime) were used to detect nuclei and DNA fragments [10].

### 2.3. RNA-Sequencing and Transcriptome Analysis

RNA from three mycelial samples (three independent replicates per sample) was extracted using TRIZOL^®^ Reagent (Invitrogen, Waltham, MA, USA). RNA quality and integrity were evaluated using a NanoDrop 2000 spectrophotometer (Thermo Fisher Scientific, Waltham, MA, USA) and an Agilent 2100 bioanalyzer (Agilent Technologies, Santa Clara, CA, USA), respectively. Library construction and RNA-sequencing were conducted by Novogene Bioinformatics Technology Co., Ltd. (Beijing, China). cDNA libraries were constructed using the NEBNext^®^ Ultra™ RNA Library Prep Kit for Illumina^®^ (NEB, Ipswich, MA, USA). After the library was qualified, different libraries were pooled according to the effective concentration and machine data, then sequenced using an Illumina platform with 150 bp paired-end reads.

Quality control of the raw data was performed using the FastQC plugin of TBTools v1.09876 [12]. After removing low-quality pair reads and adaptor contamination using the Trimmomatic plugin, the clean reads were spliced and aligned to the reference *L. edodes* strain SP3 (https://ngdc.cncb.ac.cn/bioproject/browse/PRJCA007678, accessed on 27 December 2021) using the Hisat2 plugin. Gene expression levels were measured based on fragments per kilobase of transcript per million fragments mapped (FPKM), and the expression matrix was obtained using the StringTie quantification plugin. Principal co-ordinates analysis (PCOA) was applied to a matrix of Bray–Curtis coefficients and performed using Omicshare PCOA tools (https://www.omicshare.com/tools/Home/Soft/pcoa, accessed on 2 December 2022).

The WGCNA Shinny R package was used to perform the subsequent analyses (https://gitee.com/shawnmagic/swtbplugin, accessed on 17 March 2021). The low-expressed genes were prefiltered, and 8865 genes were finally used in the WGCNA analysis. We calculated the pick Soft Threshold to determine the optimal soft threshold parameter (β) for the distribution of a scale-free network. Expressed similar genes were classified into one category, and modules were established. The co-expression matrix was constructed by a one-step method. According to the coefficient relationship between phenotype and module, the larger the correlation coefficient, the higher the correlation between the module and the phenotype. The expression module related to the target trait was selected to further mine the differentially expressed genes (DEGs). DEGs were screened using DESeq2 (|log2(fold change)| > 0, padj < 0.05) and subjected to Kyoto Encyclopedia of Genes and Genomes (KEGG) pathway enrichment analysis.

### 2.4. Antibody Preparation and Western Blotting

To obtain a LeATG8-specific rabbit polyclonal antibody, we amplified LeAtg8 from the cDNA of *L. edodes* mycelia using the following primer pair: 5′-GAATTCATGAGGTCAAAGTTCAAGGACGAGC-3′ (forward) and 5′-CTCGAGTCATGCATCCATCGGCAGCT-3′ (reverse). A pET-28a(+)-LeAtg8 recombinant plasmid was constructed and transformed into *Escherichia coli* BL21 (DE3) cells. After ultrasonication, LeATG8 in the supernatant was eluted with buffer containing 500 mM imidazole on a Ni-Sepharose column. The purified LeATG8 protein was obtained after ultrafiltration and dialysis (Figure S1). An anti-LeATG8 antibody was raised in rabbits by sq-biotech (Beijing, China).

The loading amount of each sample was normalized based on the protein concentration of the sample. The loaded protein was separated using SDS-PAGE and transferred onto a polyvinylidene fluoride membrane at 120 mA for 1 h. After blocking, the membrane was further incubated with the anti-LeATG8 primary antibody and goat anti-rabbit IgG H&L secondary antibody (Abcam, Cambridge, MA, USA) at a 1:1000 dilution. Immunoreactive bands were detected using a ChemiDoc imaging system (Bio-Rad, Hercules, CA, USA). 

Phospho-p38 mitogen-activated protein kinase MAPK (Thr180/Tyr182) (D3F9) XP^®^ rabbit antibody (Cell Signaling Technology, Danvers, MA, USA) was used to analyze the phosphorylation level of *L. edodes* high osmolarity glycerol 1 (LeHOG1), whereas a p38 MAPK (D13E1) XP^®^ rabbit antibody (Cell Signaling Technology) was used as the control.

Total protein was used for normalization for quantitative Western blot analysis. We stained gels with Coomassie brilliant blue after SDS-PAGE and detected total protein bands. The normalized target protein expression to total protein expression was analyzed using the ChemiDoc imaging system.

### 2.5. Statistical Analysis

The SOD activity, JC-1-labeled MMP, relative total protein contents, and transcription levels (FPKM) of *LeAtg8*, *LeHog1*, *LePbs2*, *LemTOR*, *LeTubulin*, *LeActin*, *LeH3*, and *LeGAPDH* obtained in this study are presented as means ± standard deviations (SDs). The numbers of biological replicates in each experiment are noted in the figure legends. Statistical analysis was performed by two-tailed Student’s *t*-tests. 

## 3. Results

### 3.1. Aging Characteristics of L. edodes Mycelia

**Figure 1:**
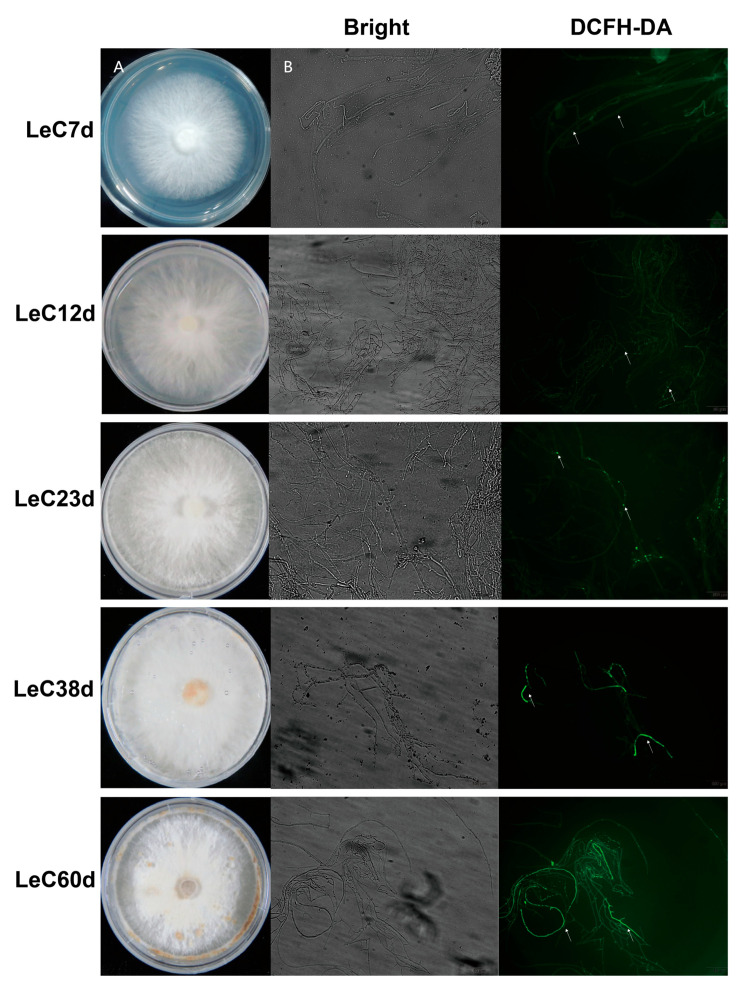
Aging characteristics of *Lentinula edodes* mycelia. (**A**) The phenotypes of colonies under different culture times. (**B**) DCFH-DA staining marked the reactive oxygen species (ROS) contents of mycelia cultured for different numbers of days. The white arrows mark ROS fluorescence.

In order to better determine the degrees of mycelial aging, we systematically analyzed the colony phenotypes, subcellular structural changes, ROS contents, and antioxidant enzyme activities in mycelia cultured for different periods. Colony phenotyping showed that aerial hyphae increased with longer culture time. Mycelial browning was observed after 38 days of culture. Following culture for 60 days, the number of browning mycelia increased, the edges of colonies shriveled, and spherical bulges appeared on the colonies (Figure 1A). 

Accumulating evidence suggests that oxidative damage is a key cause of aging [13]. Thus, intracellular ROS levels were detected with DCFH-DA staining. DCFH-DA is an ROS indicator on account of its highly fluorescent oxidized product being generally proportional to the number of intracellular ROS. At 12 days, the fluorescence signal was almost the same as for the control (7 days). After 23 days, the fluorescence area of ROS in mycelium cells increased gradually with the increase in culture time, indicating that the accumulation of ROS increased in the aging process (Figure 1B). SOD catalyzes the breakdown of superoxide into hydrogen peroxide and water and therefore the most important defenses against oxygen radicals [13]. The enzyme activity of SOD initially increased with culture time and then decreased, peaking after 23 days of culture. Even at 60 days, significantly higher SOD activity was observed than at 7 and 12 days (Figure S2A). These results could demonstrate that both the production and regulation of ROS were enhanced in mycelial long-term culture.

**Figure 2:**
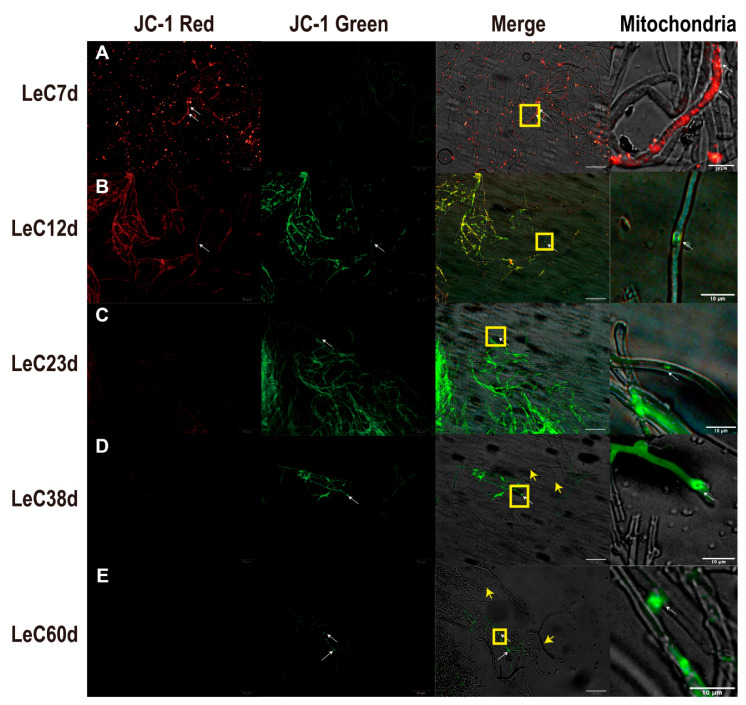
Mitochondrial membrane potential (MMP) changes based on JC-1 staining in mycelia. (**A**) MMP of mycelia under 7 days; (**B**) MMP of mycelia under 12 days; (**C**) MMP of mycelia under 23 days; (**D**) MMP of mycelia under 38 days; (**E**) MMP of mycelia under 60 days. Red fluorescence indicated that J-aggregates formed in the mitochondrial matrix, and the mitochondrial membrane potential was high. Green fluorescence indicated that JC-1 appeared as a monomer, and the mitochondrial membrane potential was low. The mitochondria images are the yellow frame enlarged images of merge. The white arrows mark JC-1 labeled mitochondria. The yellow arrows mark empty cells without mitochondria. Scale bars for JC-1 red, JC-1 green, and merge images: 50 µm. Scale bars for mitochondria: 10 µm.

The increased production of reactive oxygen species will trigger mitochondrial membrane potential depolarization (MMP) and activate pro-apoptotic signaling [14]. As the major source of ROS in cells, mitochondria appear to be key targets of oxidative damage during aging [13]. When mitochondria are damaged by ROS, the MMP will decrease and lead to further efforts of depolarization. Qualitative and quantitative JC-1-based analyses of mycelial MMP revealed that, at 7 days, MMP was high, with mainly red fluorescence observed in cells as health status (Figure 2A and Figure S2B). After culture for 12 days, MMP decreased, and yellow fluorescence appeared in the mycelia (Figure 2B). After culturing for 23 days, MMP continued to decrease, and the mycelia exhibited mainly green fluorescence as depolarized status (Figure 2C). After culture for 38 and 60 days, the green fluorescence gradually decreased, and most mycelia showed no fluorescence (Figure 2D,E). Terminal deoxynucleotidyl transferase dUTP nick-end labeling (TUNEL) assays are commonly used to investigate cleaved and fragmented levels of chromosomal DNA in apoptosis [10]. DAPI-labeled blue nuclei were observed in mycelia cultured for 7 days; in contrast, TUNEL-labeled green fluorescent DNA fragments were not obvious (Figure S3A). After 12 days of culture, the green fluorescence of TUNEL-labeled DNA fragments appeared in individual hyphal cells (Figure S3B) and increased in the mycelia after 23 days (Figure S3C,D). At 60 days, the number of DAPI-labeled nuclei in the mycelia decreased, with fragmented DNA and predominantly non-nucleated cells (Figure S3E). The above results indicate that greater mitochondrial depolarization and DNA fragmentation were observed with longer culture time. At 38 and 60 days, vacuolization of the mycelia was observed, which may be related to autophagy or apoptosis (Figure 2D,E and Figure S3E).

### 3.2. Raw Data and Quality Assessment of the L. edodes Mycelial Aging Transcriptome

**Figure 3:**
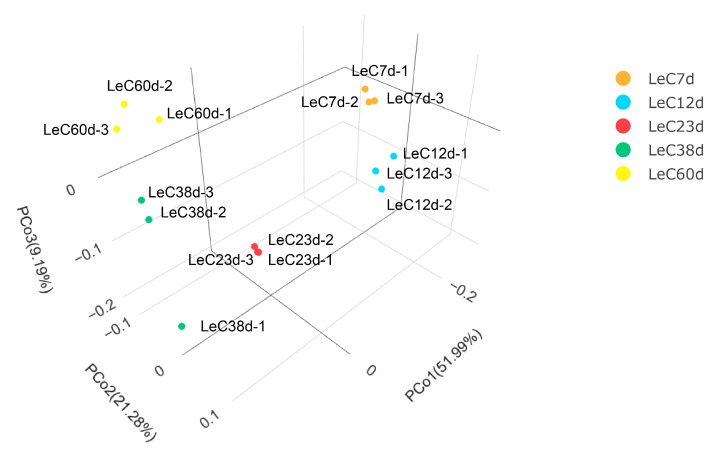
Principal coordinates analysis (PCOA) of all the samples.

Raw RNA-sequencing data are available at the National Genomics Data Center, China National Center for Bioinformation, under BioProject ID PRJCA012101. A total of 649 Mb clean reads with Q20 values greater than 97% were obtained, with an average of 89.22% reads mapped (Tables S1 and S2). The lowest Pearson correlation coefficient between biological replicates was 0.77 (Figure S4). Except for LeC38d, the Pearson correlation coefficients for the other three repeated samples were approximately 0.9. Among the three samples in the LeC38d treatment group, the correlation coefficients between LeC38d-1 and the other two samples were lower. To further analyze the correlation between samples, we employed principal coordinates analysis (PCOA) to conduct dimensionality reduction on the gene count data for each sample. PCOA 3D plot results showed that the distance between LeC38d-1 and the other samples was relatively large, while those between replicate samples of the other groups were relatively small (Figure 3). Therefore, we excluded the RNA-sequencing data for LeC38d-1 from subsequent analyses.

Ten DEG categories (LeC7d vs. LeC12d, LeC7d vs. LeC23d, LeC7d vs. LeC38d, LeC7d vs. LeC60d, LeC12d vs. LeC23d, LeC12d vs. LeC38d, LeC12d vs. LeC60d, LeC23d vs. LeC38d, LeC23d vs. LeC60d, and LeC38d vs. LeC60d) were compared to understand *L. edodes* mycelial aging (Figure S5). Compared to LeC7d, LeC12d, LeC23d, LeC38d, and LeC60d had 6477 (3304 upregulated and 3173 downregulated), 7402 (3776 upregulated and 3626 downregulated), 5987 (3174 upregulated and 2813 downregulated), and 7321 DEGs (3753 upregulated and 3568 downregulated), respectively. With longer culture time, the number of DEGs in each category gradually decreased. The number of DEGs between LeC38d and LeC60d was 2025 (976 upregulated and 1049 downregulated), which was less than a third of those between LeC7d and LeC60d. PCOA analysis indicated that LeC38d and LeC60d were relatively close to each other, having similar subcellular and colony morphological phenotypes. Therefore, these two datasets were considered in the subsequent mining of aging-related genes.

### 3.3. Exploring Aging-Related Genes in L. edodes via WGCNA

WGCNA was used to analyze the FPKM data of mycelia at different culture times, to select the genes related to aging, and to construct a gene co-expression network. We calculated the pick Soft Threshold (Table S3) to determine the optimal soft threshold parameter (β). The network tends infinitely toward the distribution of a scale-free network. Through preliminary calculations and repeated verification, we selected the value of (β) = 7, which had a correlation coefficient greater than 0.8, for further analysis (Figure S6 and Table S4). A gene cluster dendrogram was then constructed (Figure S7). Genes with similar expression patterns were integrated into the same modules. The grey modules represent genes that could not be integrated into any other module.

**Figure 4:**
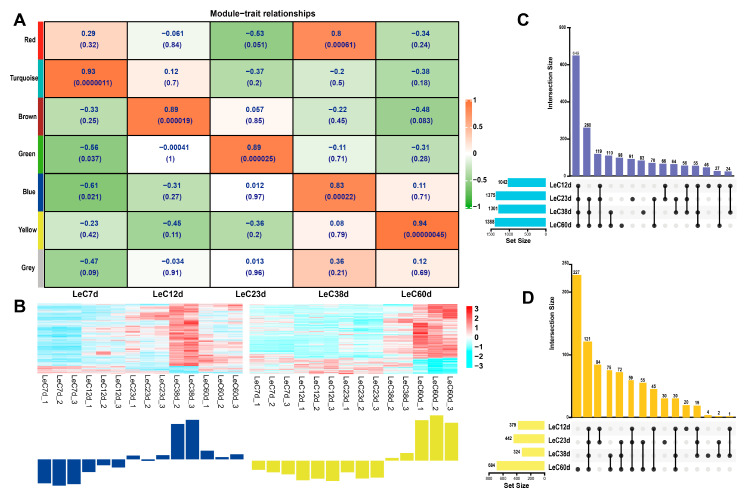
WGCNA and UpSet Venn analysis selection of aging-related genes in *L. edodes*. (**A**) Module−trait relationships. The more red the color of the module, the larger the correlation coefficient R. The upper number in the module indicates the correlation coefficient, and the number below indicates the *p*-value. (**B**) Heatmap of gene expression in blue and yellow modules. Red represents a high level of transcription, and blue represents a low level of transcription. (**C**) UpSet Venn diagram of the blue module. (**D**) UpSet Venn diagram of the yellow module.

In this study, genes were divided into seven modules, six of which were correlated with the physiological data for five culture times (Figure 4A). Here, we selected the blue module that contained 2187 genes (Table S4) and exhibited a significant positive correlation with *LeC38d* (r = 0.8, *p* < 0.01), as well as a yellow module that contained 945 genes (Table S4) and was significantly positively correlated with *LeC60d* (r = 0.94, *p* < 0.01). While the red module also showed a significant positive correlation with *LeC38d* (r = 0.83, *p* < 0.01), data suggested a positive relationship with *LeC7d*. Therefore, the red-module genes were not used for subsequent analyses. Expression heat map analysis showed that the genes of the blue and yellow modules were mostly upregulated, with their expression levels in *LeC38d* and *LeC60d* being significantly higher than those in the other groups (Figure 4B).

### 3.4. KEGG Enrichment and Co-Expression Network Analyses of Aging-Related Module Genes

**Figure 5:**
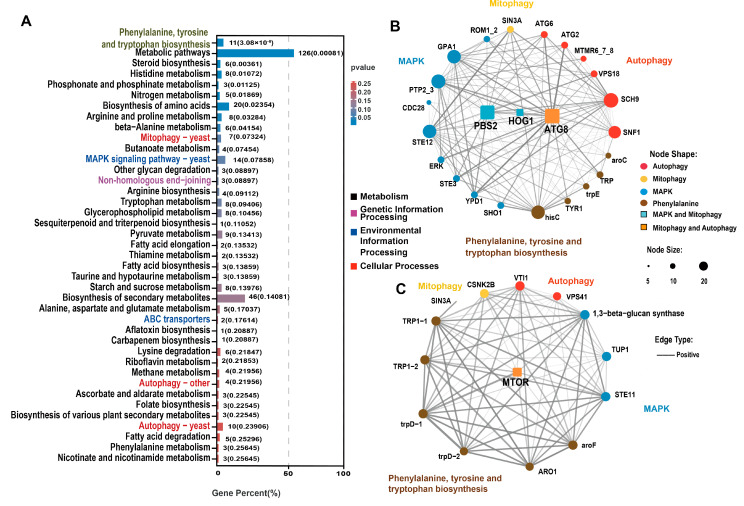
KEGG enrichment and co-expression network of aging-related genes. (**A**) KEGG enrichment of blue and yellow-module genes. Black and grey pathways belong to the “metabolism” class; magenta pathways belong to the “genetic information processing” class; blue pathways belong to “environmental information processing”; red pathways belong to the “cellular process” class; and grey pathways are related to melanin formation. The numbers outside the brackets are the numbers of enriched genes. The numbers in brackets are *p*-values. The blue color of the bar indicates that the *p*-value was small. (**B**) The co-expression network diagram of genes contained in the blue module. (**C**) The co-expression network diagram of genes contained in the yellow module. Yellow dots belong to “mitophagy-yeast”; red dots belong to “autophagy-yeast”; blue dots belong to “MAPK-yeast”; grey dots belong to “phenylalanine, tyrosine and tryptophan biosynthesis”; orange dots belong to both “mitophagy-yeast” and “autophagy-yeast”; “turquoise dots” belong to both “autophagy-yeast” and “MAPK-yeast”. Larger dots indicate more genes being associated with them. A solid line indicates a weight greater than 0.3.

After further screening of the genes related to aging, we drew an UpSet Venn diagram. KEGG enrichment analysis was performed on the DEGs that were selected via WGCNA and UpSet Venn analysis to explore the key genes associated with aging. Based on the DEGs between each group and LeC7d, an UpSet Venn diagram was drawn. DEGs in the blue and yellow modules were further screened. In the blue module, we selected *LeC12d*, *LeC23d*, *LeC38d*, and *LeC60d* common DEGs; *LeC23d*, *LeC38d*, and *LeC60d* common DEGs; as well as *LeC38d* and *LeC60d* common DEGs, for a total of 1019 DEGs (Figure 4C and Table S5). In the yellow module, we selected *LeC12d*, *LeC23d*, *LeC38d*, and *LeC60d* common DEGs; *LeC23d*, *LeC38d*, and *LeC60d* common DEGs; *LeC38d* and *LeC60d* common DEGs; as well as *LeC60d* DEGs, for a total of 495 DEGs (Figure 4D and Table S6). The selected blue and yellow DEGs were combined for KEGG enrichment analysis. Thirty-four metabolic pathways were among the top 40 enriched KEGG terms (Figure 5A and Table S7). This indicated that the mycelial aging process was mainly driven by changes in metabolism. Among the 34 metabolic terms, “phenylalanine, tyrosine and tryptophan biosynthesis”, which was the top enriched term, is related to melanin formation. Three autophagy-related enriched terms were noted in the cellular processes category, namely, “mitophagy-yeast”, “autophagy-other”, and “autophagy-yeast”. In addition, “MAPK signaling pathway-yeast” and “ABC transporter”, which belong to environmental information processing and the end-joining pathway, were among the enriched terms.

In the “autophagy-yeast“ pathway, 10 genes were enriched, including 7 upregulated and 3 downregulated genes. In the blue module, which was significantly associated with the mycelium after 38 days of culture, except for the downregulated expression of myotubularin-related protein 6 (MTMR6), other autophagy-related genes were upregulated. After 60 days of culture, the expression of the above-mentioned autophagy-related genes was lower than that of 38D (MTMR6 was upregulated) yet was still significantly higher (or lower) than that of *LeC7d*. Interestingly, in the yellow module, which was significantly related to the mycelium cultured for 60 days, three autophagy-related genes were enriched. Among these, vesicle-mediated protein transport (VPS41) and Vps10 interactor 1 (VTI1) were downregulated. Similarly, seven genes were enriched in the “mitophagy-yeast” pathway, of which six were upregulated and one was downregulated. The genes in the blue module were upregulated. Our results indicated that autophagy and mitophagy in the mycelia of *L. edodes* peaked at 38 days. After 60 days of culture, although still at high levels, both autophagy and mitophagy began to decrease.

Based on the “phenylalanine, tyrosine and tryptophan biosynthesis”, “mitophagy-yeast”, “autophagy-other”, “autophagy-yeast”, and “MAPK signaling pathway-yeast” terms, co-expression network diagrams of the genes contained in the blue and yellow module were drawn, respectively (Figure 5B,C). In the blue module, we selected three DEGs of network hubs in at least two pathways. These included the *LeAtg8* gene, which mediates both autophagy and mitophagy, as well as dual specific kinase (*LePbs2*) and *LeHog1*, which mediate both the MAPK and mitophagy pathways (Figure 5B). In the yellow module, we selected only one hub gene found in at least two pathways, the mammalian target of rapamycin (*LemTOR*) gene, which mediates both autophagy and mitophagy (Figure 5C).

### 3.5. LeHOG1 and LeATG8 Protein Expression in Aging Mycelia of L. edodes

**Figure 6:**
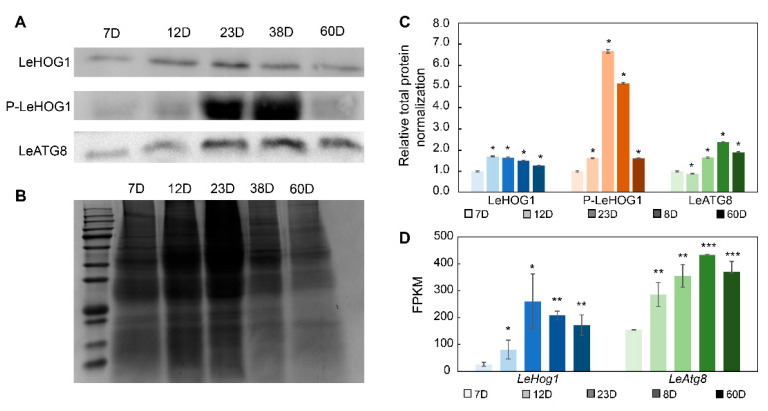
Related protein variation in *Lentinula edodes* mycelia under long-term culture. (**A**) Western blots of LeHOG1 phosphorylation and LeATG8 expression. (**B**) SDS-PAGE of total protein. (**C**) Normalization of Western blotting protein level data. (**D**) FPKM values of LeHog1 and LeAtg8 gene transcription. The blue color presents LeHOG1, orange color presents LeHOG1 phosphorylation, and green color presents LeATG8. Color deepening represents increasing culture days. Means ± SDs; * *p* ≤ 0.05, ** *p* ≤ 0.01, *** *p* ≤ 0.001 (two-tailed Student’s *t*-tests).

In view of Atg8-mediated autophagy and the key role of the HOG1 homologous protein P38-mediated P38 MAPK pathway in cell aging [15,16], we analyzed the protein expression of the key proteins LeATG8 and LeHOG1 in the blue module during the aging of *L. edodes* mycelium by Western blotting. Hog1 is activated by dual phosphorylation of a tripeptide motif (Thr-X-Tyr), thereby driving oxidative stress and other responses to environmental regulation. We also analyzed the degree of phosphorylation of LeHOG1 protein in *L. edodes* mycelia. At the end of mycelium culture, the transcript levels of various housekeeping genes, such as histone 3, tubulin, actin, and GAPDH, were significantly altered (Figure S8). We did not find a suitable internal control for Western blotting. Therefore, we normalized target protein expression to total protein expression. Longer culture time resulted in a gradual increase in LeATG8 protein levels. The levels of phosphorylated LeHOG1 on the 23rd and 38th days were significantly higher than those on the other days. There was no significant change in LeHOG1 protein levels (Figure 6).

## 4. Discussion

Aging is the process of progressive decline in organismal functions, which culminates in death [17,18]. PCD is an important characteristic feature of aging filamentous fungi [4]. Fungal PCD can be divided into apoptosis and autophagic cell death, which are driven by distinct mechanisms [19]. The main characteristics of PCD include chromatin condensation, DNA fragmentation, morphological as well as functional alterations in mitochondria, and the degradation of cytoplasmic regions or entire organelles [19]. Similar to the aging-model fungus *P. anserina*, mycelial pigment was observed in *L. edodes* after 38 days of culture, and aerial mycelia decreased, in addition to the shriveling of mycelium edges, after 60 days of culture. Further, DNA fragmentation and mitochondrial membrane depolarization were noted in aged mycelia via TUNEL and JC-1 staining, respectively. Previous studies demonstrated characteristic phenomena of apoptosis and autophagic cell death, such as nuclear condensation as well as organelle lysis, in *L. edodes* mycelia under long-term culture [10,11]. Taken together, these findings support roles for apoptosis and autophagy in mycelial aging. 

Transcriptome analysis provided further evidence of the involvement of autophagy in mycelium aging. In the cellular process category, the three enriched pathways were related to autophagy and mitophagy, as the yellow-module hub gene rapamycin complex (TOR or mTOR) encodes a negative regulator of autophagy and mitophagy in yeast [20]. mTOR exerts negative regulation on the ATG1-ATG13-ATG17 complex, or indirectly inhibits class III phosphoinositide 3-kinase (PI3K) components (ATG6, ATG14, and vesicle-mediated protein transport 34 (VPS34)), leading to the inhibition of autophagy [21,22,23]. The negative regulator mTOR was upregulated in the yellow module (Figure S8), while the expression of casein kinase 2β (CSNK2B) was downregulated. Switch-independent 3a (SIN3A) has two homologous genes, gene-*Led05903-sp3* in the blue module and gene-*Led02394-sp3* in the yellow module. However, the expression of gene-*Led05903-sp3* was significantly higher than that of gene-*Led02394-sp3*. We consider that gene-*Led05903-sp3* in the blue module plays a leading role in the expression of SIN3A. Our results indicated that mTOR plays a key role in aging through its effects on proteostasis and mitochondrial function [24]. In mycelia under long-term culture, the expression of mTOR was significantly increased only at 60 days.

Another hub gene, ATG8, which participates in both the autophagy and mitophagy pathways, is a key ubiquitin-like protein and autophagosome component [25]. ATG8 can bind to phosphatidylethanolamine (PE) under the action of ubiquitin-activating enzyme E1, ubiquitin-conjugating enzyme E2, and ubiquitin ligase E3, thereby anchoring to the autophagosome membrane and outer membrane to help autophagosome expansion [26]. ATG8 protein is thus widely used to indicate autophagosomes and assess autophagic activity [27]. In our study, the LeATG8 protein was highly expressed after 23 days of culture, and its expression level began to decrease on day 60, in agreement with transcriptome data. LeATG8 protein expression may reflect changes in autophagic activity in *L. edodes* mycelia. In multiple fungi, autophagy and mitophagy have established roles in the control of aging [8,28,29]. In *P. anserina*, the expression of genes encoding autophagic machinery increased with age, followed by a subsequent decrease in autophagic activity at very old age [8]. Combined with the expression of mTOR and CSNK2B, the autophagy levels of *L. edodes* mycelia decreased after 60 days of culture, and the numbers of dead cells in the mycelia increased further, indicative of aging. 

ROS are another key factor associated with various functions, including autophagy, aging, mycelial growth, and cell cycle progression [7]. ROS accumulation causes mitochondria-dependent PCD, which is considered the key event underlying biological aging in *P. anserina* [30,31]. Intracellular ROS levels are regulated through the activity of multiple antioxidant enzymes, including SOD [8]. In this study, intracellular ROS levels I and SOD activity increased with *L. edodes* aging. ROS can induce autophagy by regulating the mTOR and MAPK signaling pathways [32]. The MAPK pathway includes c-Jun amino-terminal kinase (JNK), p38 kinase (Homology HOG1 in yeast), and extracellular signal-regulated kinase (ERK). The p38 (HOG1) signaling pathway is involved in ROS-activated autophagosome/lysosomal fusion [32]. In *Saccharomyces cerevisiae*, PBS2 can activate HOG1 by phosphorylating specific Thr174 and Tyr176 residues in the HOG1 T-G-Y motif [33]. HOG1 phosphorylation leads to autophagy-related gene phosphorylation and activation, while loss of HOG1 leads to high levels of ROS production [34]. Therefore, HOG1 maintains intracellular ROS homeostasis and regulates autophagy. During the aging process of *L. edodes* mycelia, we noted the co-expression of the *LeHog1*, *LePbs2*, and *LeAtg8* genes. Moreover, Western blotting results showed that LeHOG1 phosphorylation and LeATG8 protein levels followed the same trend. Now, ROS and the autophagy mechanism and their functions in relation to mushroom growth and fruit-body formation are becoming hot research topics [10,35,36]. Thus, the correlation between ROS and autophagy in *L. edodes*, as well as the role of LeHOG1 protein in ROS-mediated oxidative stress, require further study. We will identify the gene functions of *LeAtg8* and *LeHog1* in our further work.

Phenylalanine, tyrosine, and tryptophan are precursors of a wide variety of secondary metabolites that play crucial roles in growth, development, reproduction, defense, and environmental responses [37]. In particular, phenylalanine and tyrosine are precursors of arylpyruvic, cinnamic, and benzoic acids, which represent the building blocks for various pigments in mushrooms [38]. Both phenylalanine and tyrosine, as well as their products, can be converted via quinones into the heterogeneous dark pigment melanins through enzymatic browning reactions. Based on ultraviolet, visible, and infrared spectra, as well as elemental analyses, it was predicted that the pigment of *L. edodes* brown mycelia was melanin [11]. The *L. edodes* genome does encode machinery necessary for the complete synthesis of eumelanin, GHB melanin, PAP melanin, and catechol melanin [39]. In the 3,4-dihydroxyphenylalanine (DOPA) synthesis pathway, chorismate production is catalyzed by prephenate dehydrogenase. Under the activity of 4-hydroxyphenylpyruvate aminotransferase and polyphenol oxidase/tyrosinase, tyrosine and L-DOPA, key metabolites in the tyrosine-dopa synthesis pathway, are formed [39]. In this study, we found that the expressions of the *Led05523-sp3* gene encoding prephenate dehydrogenase (TYP1) and the *Led05850-sp3* gene encoding polyphenol oxidase/tyrosinase were significantly upregulated after 38 and 60 days of culture. In our previous studies, we reported that mycelia formed brown films during long-term culture, as reported in this article; furthermore, we observed the melanin accumulation process at the cell walls, intracellular spaces, and intercellular spaces of the mycelia in brown films, which suggested that contents of melanin increased during long-term culture [11]. The accumulation of melanin during long-term culture is consistent with the “phenylalanine, tyrosine and tryptophan biosynthesis term” being enriched during long-term culture in this article. Therefore, we speculate that the pigment produced under long-term cultivation of *L. edodes* may be eumelanin or pheomelanin via the DOPA pathway.

Besides gene expression regulation, gene mutation also might be involved in strain degeneration by inducing aging. The generation of ROS has been implicated in mtDNA mutation and aging; meanwhile, organisms have many repair systems that respond to DNA damage, such as mutations caused by ROS [40,41]. Here, we found base excision repair genes, e.g., AP endonuclease 1 (gene-*Led05208-sp3*) and endonuclease 3 (gene-*Led021048-sp3*), and nucleotide excision repair genes, e.g., transcription initiation factor (gene-*Led05557-sp3*), and DNA excision repair protein (gene-*Led00888-sp3*) presented in blue-module DEGS. These results support that DNA mutations in aging mycelia accumulated in long-term culture would be repaired by the DNA repair system.

## 5. Conclusions

In summary, physiological phenotypes and microstructures were characterized during the intrinsic aging process of *L. edodes* mycelia over 60 days of culture. We found that ROS accumulation, mitochondrial depolarization, and DNA degradation occurred during mycelial aging. Genome-wide transcriptional responses were compared, with numerous growth- and aging-associated DEGs identified under long-term culture. Our results further clarified that LeATG8 and phospho-LeHOG1 showed time-dependent changes at the transcriptional and protein levels, indicating that autophagy/mitophagy is involved in mycelial aging. These findings pave the way for further investigation into the mechanisms that drive edible fungus aging. Future work will focus on the functions and interactions of ATG8 and HOG1 in the context of fungal aging.

## Data Availability

All data generated or analyzed during this study are included in this published article and its supplementary information files. The raw sequencing data of RNA-seq are available at the National Genomics Data Center, China National Center for Bioinformation, under BioProject ID PRJCA012101 (https://ngdc.cncb.ac.cn/bioproject/browse/PRJCA012101, accessed on 17 March 2023). The sample LeC7d was name LeC1.

## Supplementary Materials

The following supporting information can be downloaded at: https://www.mdpi.com/article/10.3390/jof9030379/s1. File contains Figures S1–S8 and Tables S1–S7. Figure S1: SDS-PAGE-tested purified LeATG8 protein; Figure S2: DCFH-DA staining marked the reactive oxygen species (ROS) contents of mycelia cultured for different numbers of days; Figure S3: Nuclei and DNA fragments were stained via DAPI and TUNEL staining; Figure S4: Pearson correlation values between biological replicates; Figure S5: The number of DEGs among the ten categories; Figure S6: The soft connectivity and correlation coefficient when the soft threshold parameter was 7; Figure S7: Construction of scale-free network and module classification; Figure S8: Transcription of DEGs of network hubs and housekeeping genes; Table S1: Data summary of RNA-seq; Table S2: Summary of clean reads mapped to reference genome; Table S3: Information on soft threshold; Table S4: The numbers of genes in co-expressed modules; Table S5: Blue-module DEGS list filtered by Upset Venn diagram; Table S6: Yellow-module DEGS list filtered by Upset Venn diagram; Table S7: The top 40 enriched KEGG terms of DEGs in blue and yellow modules.
