# Supplementary material for: Insights into the Global Transcriptome Response of Lentinula edodes Mycelia during Aging
Source: J Fungi (Basel). 2023 Mar 20;9(3):379. doi: 10.3390/jof9030379 (PMC10057243; doi:10.3390/jof9030379)
Supplement: Supplementary file 1 [file jof-09-00379-s001.zip › jof-2214389-supplementary.pdf]

Supplement materials

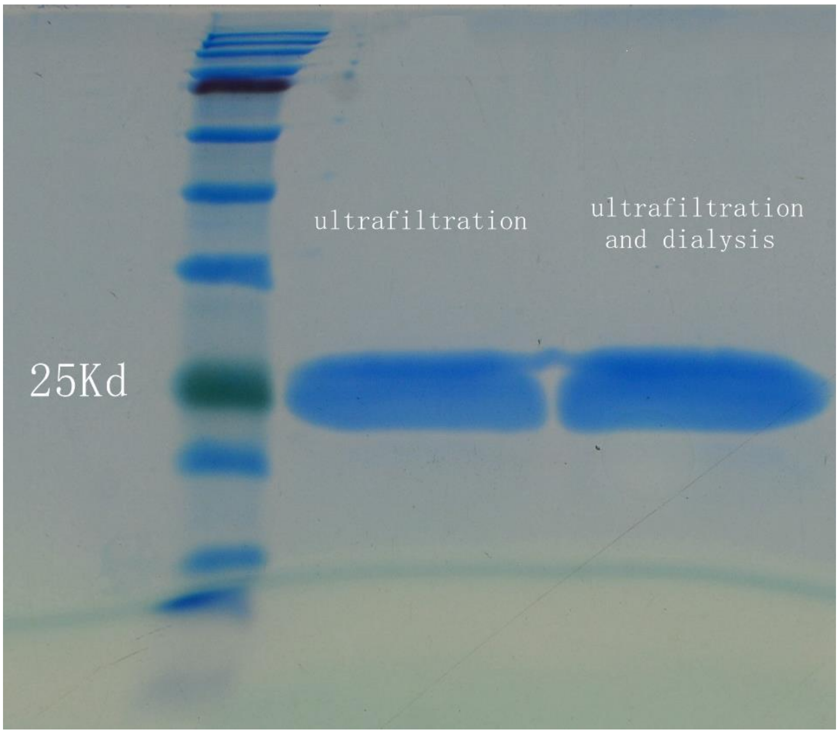

Figure S1. SDS-PAGE tested purified LeATG8 protein**.**

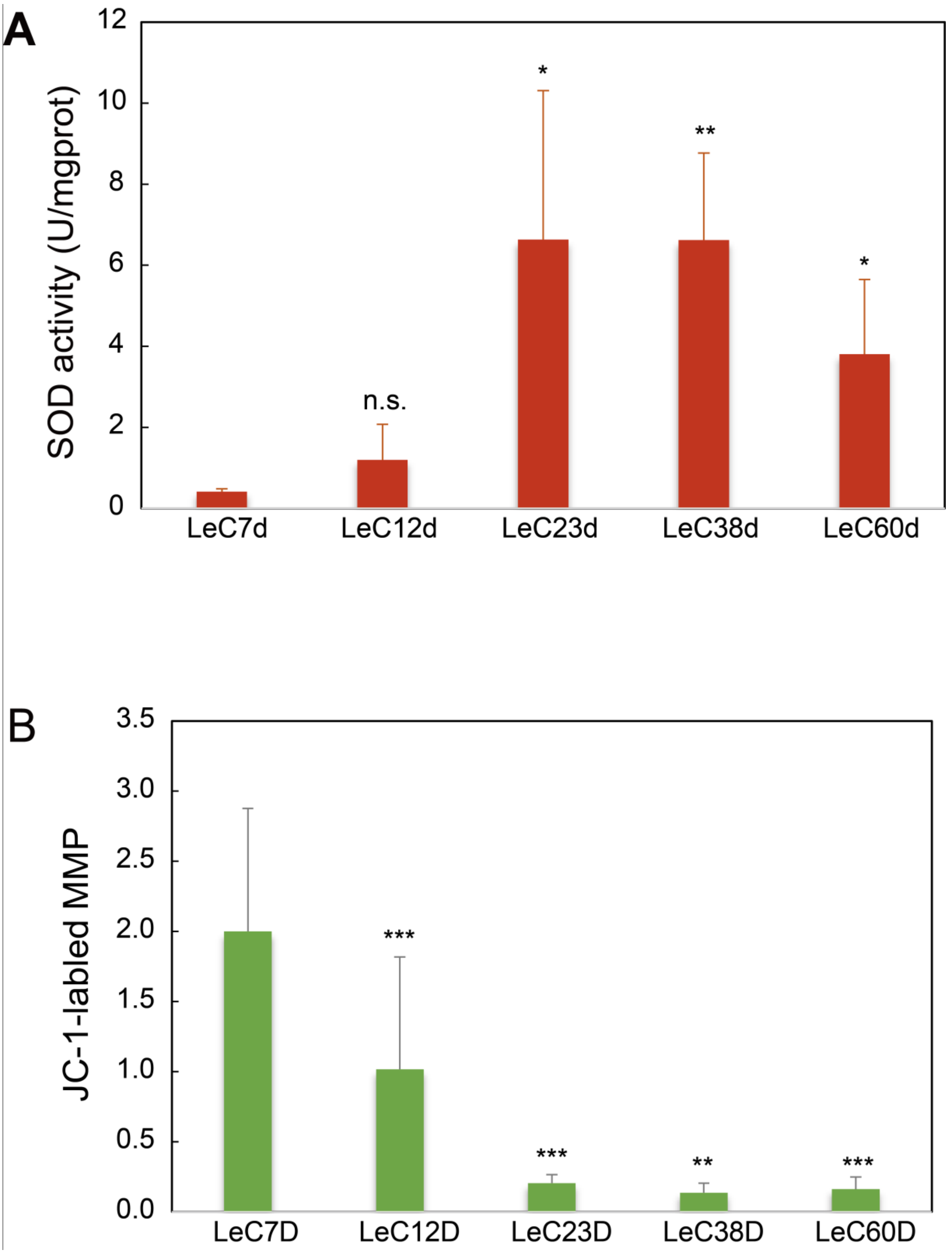

Figure S2. Superoxide dismutase (SOD) activity and JC-1 labeled mitochondrial membrane potential in mycelia under different times . (**A**) SOD activity of mycelia under different culture times. Mean ± SD, n = 3. n.s., no significance, * $p \leq 0.05$, ** $p \leq 0.01$ (two-tailed Student's t-test). (**B**) Quantitative analysis of mitochondrial membrane potential changes based on JC-1 staining in mycelia under different times. Mean ± SD, n = 100. n.s., no significance, ** $p \leq 0.01$, *** $p \leq 0.001$ (two-tailed Student's t-test).

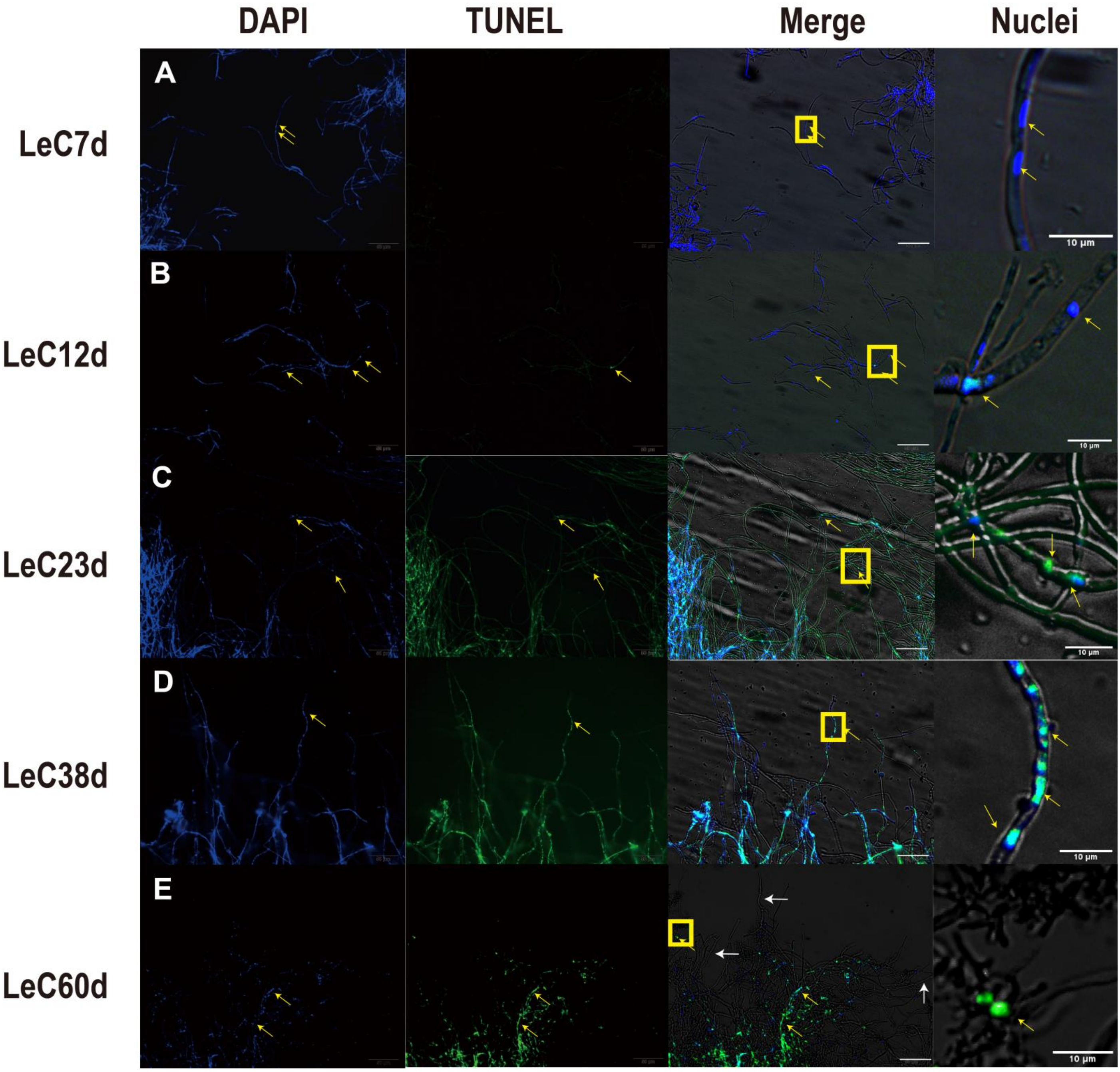

Figure S3. Nuclei and DNA fragments were stained via DAPI and TUNEL staining. (A) 7 days **mycelia**; (B) 12 days **mycelia**; (C) 23 days **mycelia**; (D) 38 days **mycelia**; (E) 60 days **mycelia**. Blue fluorescence indicates DAPI-labeled nuclei. Green fluorescence indicates TUNEL-labeled DNA fragments. The yellow arrow was DAPI or TUNEL labeled nuclei. The white arrow was empty cell without mitochondria. Scale bar of DAPI, TUNEL and merge was 50 μm. Scale bar of mitochondria was 10 μm.

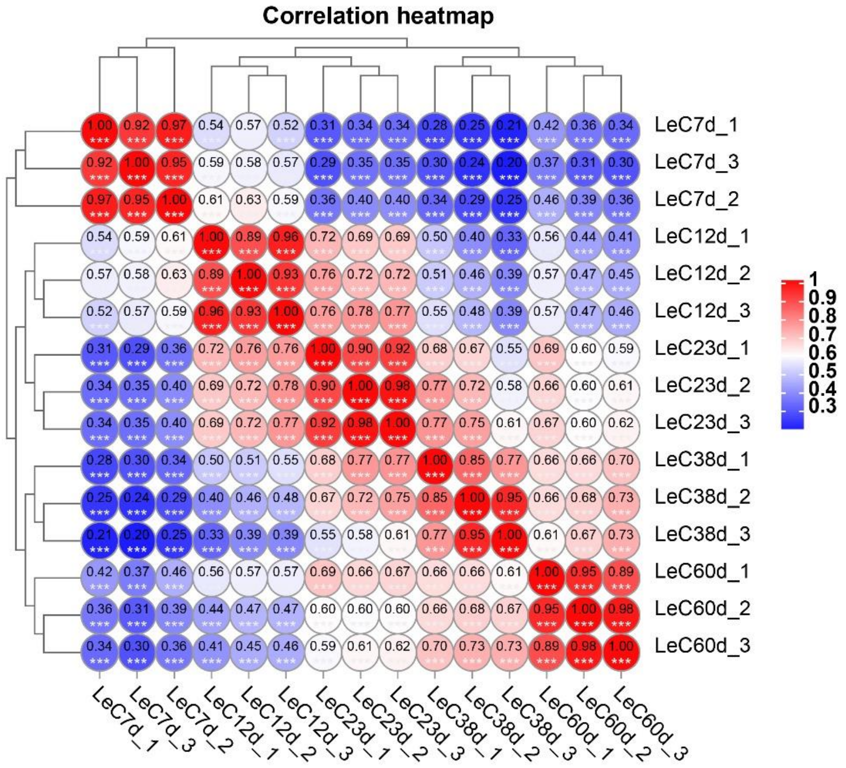

Figure S4. Pearson correlation value between biological replicates.

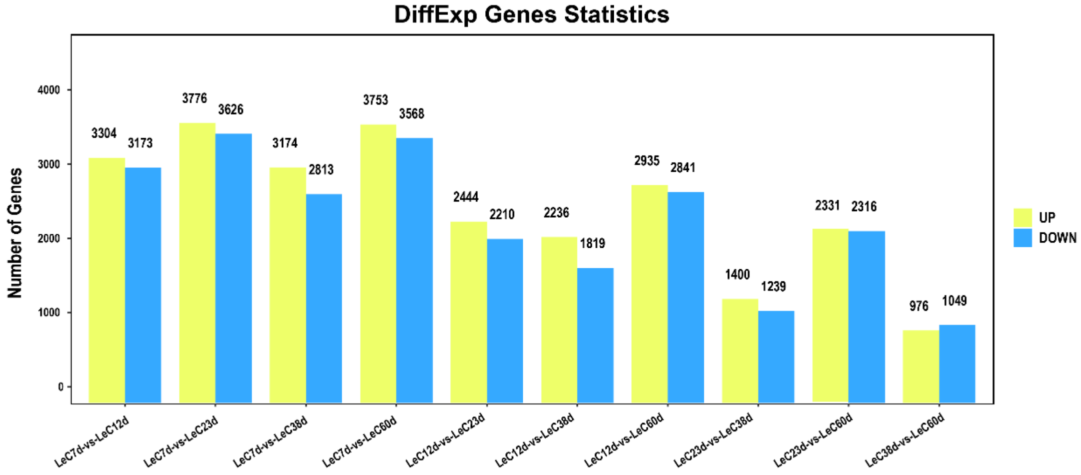

Figure S5. The number of DEGs among the ten categories. Yellow bars indicate upregulated genes. Blue bars indicate downregulated genes.

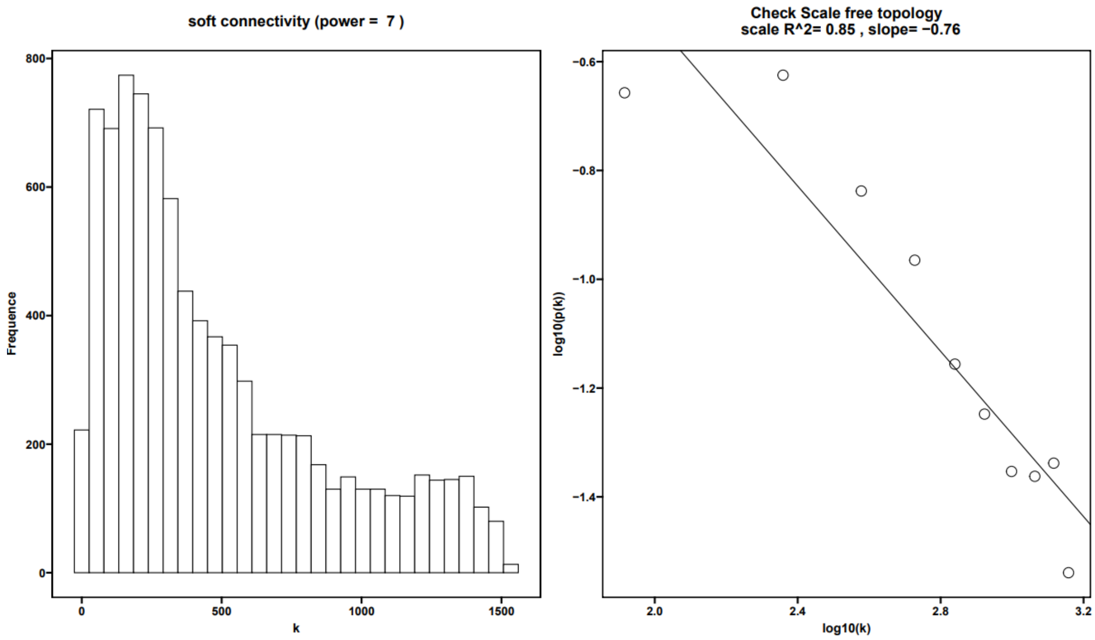

Figure S6. The soft connectivity and correlation coefficient when the soft threshold parameter was 7.

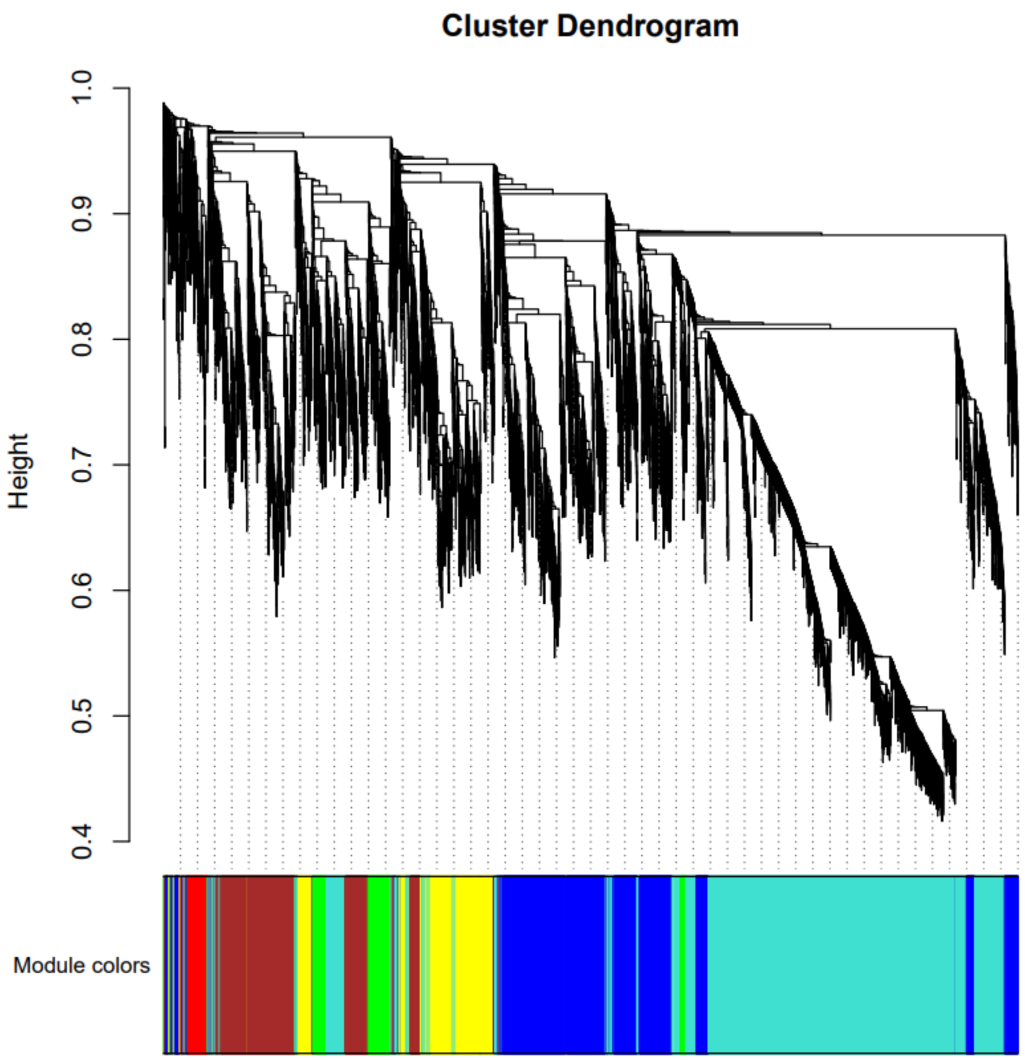

Figure S7. Construction of scale-free network and module classification. Dynamic tree clipping represents the initial module. The module color indicates the final module. Each branch in the hierarchical tree or each vertical line in the color bar represents a gene. Genes not attributed to any module are colored grey.

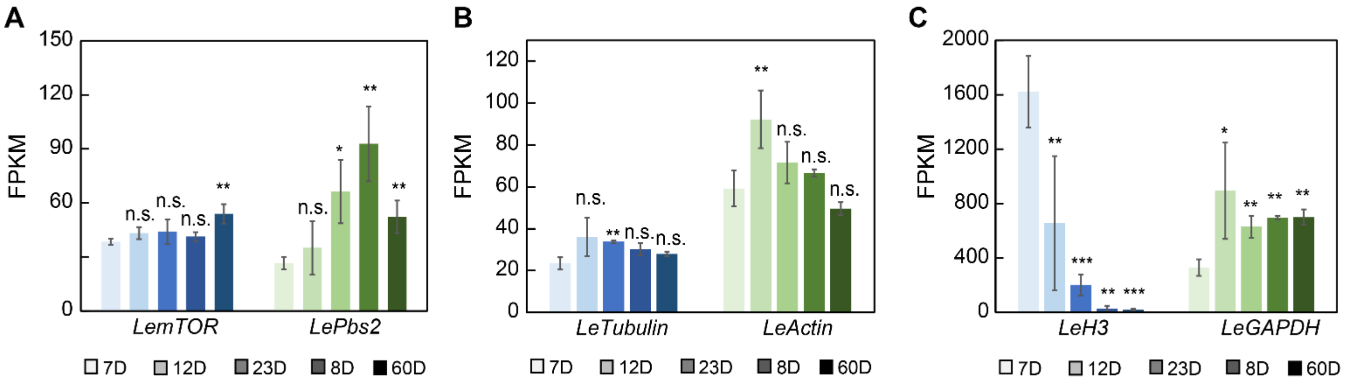

Figure S8. Transcription of DEGs of network hubs and housekeeping genes. (A) FPKM value of *LemTOR* and *LePbs2* gene. (B-C) FPKM value of housekeeping genes. Mean ± SD, * $p \leq 0.05$, ** $p \leq 0.01$, *** $p \leq 0.001$ (two-tailed Student's *t*-test).

Table S1. Data summary of RNA-seq

sample	raw_reads	clean_reads	clean_bases	error_rate	Q20	Q30	GC_pct
LeC7d_1	43100600	42305864	6.35G	0.02	98.12	94.66	48.76
LeC7d_2	46298694	45450024	6.82G	0.02	98.24	94.92	48.50
LeC7d_3	45594734	44563200	6.68G	0.02	98.35	95.16	48.50
LeC12d_1	47500690	46027784	6.90G	0.03	97.45	92.63	48.45
LeC12d_2	42526450	41598776	6.24G	0.02	97.96	94.34	48.54
LeC12d_3	41143786	40359730	6.05G	0.02	98.22	94.87	48.43
LeC23d_1	42170750	40863368	6.13G	0.02	98.28	94.96	47.95
LeC23d_2	44853426	43354786	6.50G	0.02	98.20	94.78	48.27
LeC23d_3	45469462	44619024	6.69G	0.02	98.35	95.17	48.18
LeC38d_1	45497880	44168098	6.63G	0.02	98.17	94.90	47.07
LeC38d_2	43516436	42557148	6.38G	0.02	98.22	94.85	48.20
LeC38d_3	44062466	43038134	6.46G	0.02	98.21	94.86	48.07
LeC60d_1	44997876	43741834	6.56G	0.02	98.20	94.86	48.20
LeC60d_2	43073420	41850382	6.28G	0.02	98.31	95.07	48.30
LeC60d_3	45379234	44368320	6.66G	0.02	98.16	94.78	48.25

Table S2. Summary of clean reads mapped to reference genome

sample	total_reads	total_map	unique_map	multi_map	read1_map	read2_map
LeC7d_1	42305864	39336809(92.98%)	38764697(91.63%)	572112(1.35%)	19481358(46.05%)	19283339(45.58%)
LeC7d_2	45450024	42069096(92.56%)	41442341(91.18%)	626755(1.38%)	20797776(45.76%)	20644565(45.42%)
LeC7d_3	44563200	40875358(91.72%)	40276955(90.38%)	598403(1.34%)	20190636(45.31%)	20086319(45.07%)
LeC12d_1	46027784	41580792(90.34%)	40936654(88.94%)	644138(1.4%)	20566385(44.68%)	20370269(44.26%)
LeC12d_2	41598776	37497636(90.14%)	36922224(88.76%)	575412(1.38%)	18578473(44.66%)	18343751(44.1%)
LeC12d_3	40359730	36044971(89.31%)	35511713(87.99%)	533258(1.32%)	17827632(44.17%)	17684081(43.82%)
LeC23d_1	40863368	34541113(84.53%)	33931480(83.04%)	609633(1.49%)	17007609(41.62%)	16923871(41.42%)
LeC23d_2	43354786	37359972(86.17%)	36658364(84.55%)	701608(1.62%)	18394370(42.43%)	18263994(42.13%)
LeC23d_3	44619024	37529107(84.11%)	36857797(82.61%)	671310(1.5%)	18483600(41.43%)	18374197(41.18%)
LeC38d_1	44168098	40019671(90.61%)	39318648(89.02%)	701023(1.59%)	19728582(44.67%)	19590066(44.35%)
LeC38d_2	42557148	37257002(87.55%)	36625401(86.06%)	631601(1.48%)	18390707(43.21%)	18234694(42.85%)
LeC38d_3	43038134	37578515(87.31%)	36976739(85.92%)	601776(1.4%)	18567082(43.14%)	18409657(42.78%)
LeC60d_1	43741834	40117597(91.71%)	39519645(90.35%)	597952(1.37%)	19843109(45.36%)	19676536(44.98%)
LeC60d_2	41850382	37709599(90.11%)	37133483(88.73%)	576116(1.38%)	18628132(44.51%)	18505351(44.22%)
LeC60d_3	44368320	39546111(89.13%)	38893048(87.66%)	653063(1.47%)	19530574(44.02%)	19362474(43.64%)

Table S3. Information of soft threshold

Power	SFT.R.sq	slope	truncated.R.sq	mean.k.	median.k.	max.k.
1	0.834937273	3.335990997	0.993161341	3793.659694	3843.199626	5209.611684
2	0.596908076	1.069590301	0.954557767	2191.844116	2146.232353	3748.369806
3	0.104515522	0.197404861	0.860548377	1443.717804	1339.149675	2927.755211
4	0.202250877	-0.228180346	0.820796231	1026.567165	893.5832622	2396.292733
5	0.613666752	-0.475817617	0.878623676	768.3076413	628.5514787	2022.823756
6	0.785532947	-0.639741133	0.905955236	596.7633815	458.2899545	1745.737239
7	0.865957525	-0.759546039	0.925698265	476.8410093	344.6636056	1535.289415
8	0.901976002	-0.865218548	0.928795187	389.6615043	265.2104534	1375.532213
9	0.918155112	-0.943001472	0.928713552	324.2857756	209.8163328	1246.13614
10	0.931625813	-1.003091774	0.935770204	274.0023011	167.7521859	1139.127982
11	0.928338596	-1.099574613	0.919328003	202.9093742	112.1279422	972.2115005
12	0.929796064	-1.161312985	0.916346474	156.1306649	78.92092536	847.6570316
13	0.935685726	-1.198516879	0.92011564	123.7354391	57.16707436	750.8229659
14	0.940108658	-1.221482972	0.924148579	100.3872706	42.4054574	673.1366729
15	0.935342313	-1.238945525	0.918275963	83.01118081	32.36848186	609.2553385
16	0.927470296	-1.251055789	0.90823573	69.73456854	24.83095428	555.6786663
17	0.928760535	-1.256413162	0.909921805	59.36455067	19.34245861	510.0174035
18	0.917191458	-1.267426902	0.895927723	51.11224892	15.2389853	470.5835451
19	0.918918138	-1.270274685	0.898088365	44.43917561	12.24639298	436.1490365
20	0.919447482	-1.266986415	0.899543762	38.96764017	9.963129192	405.7976169

Table S4. The number genes in co-expressed modules

Module	Gene numbers
Blue	2187
Brown	1262
Green	459
Grey	7
Red	206
Turquoise	3790
Yellow	945

Table S5 Blue module DEGS list filtered by Upset Venn diagram

gene	LeC7d_vs_LeC38d_log2FoldChange	LeC7d_vs_LeC38d_pvalue	LeC7d_vs_LeC38d_padj
gene-Led00960-sp3	1.147903181	0.012274386	0.024046703
gene-Led11025-sp3	1.027400303	0.01013666	0.020210517
gene-Led04387-sp3	0.972967846	3.26E-06	1.14E-05
gene-Led01639-sp3	2.637166358	2.57E-15	2.16E-14
gene-Led01833-sp3	0.646623906	0.000341863	0.000902117
gene-Led09931-sp3	1.455674038	9.50E-19	1.04E-17
gene-Led11178-sp3	5.348116708	7.89E-30	1.56E-28
gene-Led04493-sp3	4.909406522	5.30E-63	3.68E-61
gene-Led07964-sp3	1.845904535	5.47E-21	6.80E-20
gene-Led09395-sp3	1.052533699	0.000136879	0.000384026
gene-Led09417-sp3	2.666624175	5.39E-14	4.06E-13
gene-Led01921-sp3	5.318933528	2.97E-37	8.22E-36
gene-Led04002-sp3	4.39064993	4.60E-59	2.91E-57
gene-Led04826-sp3	5.175461743	3.75E-25	5.66E-24
gene-Led10019-sp3	-1.391173447	4.58E-13	3.17E-12
gene-Led07671-sp3	1.76803083	1.23E-05	3.97E-05
gene-Led03469-sp3	3.230568583	1.30E-32	2.86E-31
gene-Led00029-sp3	2.945404061	1.50E-17	1.50E-16
gene-Led03768-sp3	1.845398699	2.06E-13	1.48E-12
gene-Led07617-sp3	1.302181572	2.29E-10	1.27E-09
gene-Led07043-sp3	5.468008309	3.43E-207	4.93E-204
gene-Led07148-sp3	3.056977703	2.22E-06	7.96E-06
gene-Led10551-sp3	5.514636001	3.09E-22	4.10E-21
gene-Led06780-sp3	2.532688071	5.02E-08	2.20E-07
gene-Led04164-sp3	-2.782825658	1.24E-17	1.25E-16
gene-Led03800-sp3	1.819718431	1.96E-15	1.66E-14
gene-Led06091-sp3	1.610649648	9.55E-26	1.49E-24
gene-Led09649-sp3	1.425987839	9.40E-06	3.09E-05
gene-Led01824-sp3	2.505512228	9.50E-33	2.10E-31
gene-Led00214-sp3	3.629911473	8.83E-49	3.70E-47
gene-Led02706-sp3	2.388151456	1.94E-13	1.40E-12
gene-Led05844-sp3	2.947137976	8.59E-21	1.05E-19
gene-Led00086-sp3	5.302735618	2.17E-56	1.23E-54
gene-Led05744-sp3	5.516828605	1.08E-71	1.03E-69
gene-Led05902-sp3	4.223577581	4.99E-60	3.17E-58
gene-Led06278-sp3	-1.999814108	5.70E-13	3.91E-12
gene-Led02898-sp3	3.475867122	2.61E-33	5.91E-32
gene-Led02681-sp3	3.1941396	4.94E-43	1.68E-41
gene-Led03563-sp3	1.360231589	0.007320824	0.015024046

gene-Led02610-sp3	0.352890748	0.014444466	0.027874604
gene-Led06196-sp3	3.87143099	2.11E-35	5.35E-34
gene-Led05879-sp3	1.471094147	1.24E-08	5.79E-08
gene-Led03554-sp3	1.823910244	8.34E-13	5.65E-12
gene-Led01364-sp3	2.767083698	4.34E-18	4.53E-17
gene-Led06805-sp3	1.277940525	0.009213297	0.018522851
gene-Led00709-sp3	-1.159782148	9.91E-08	4.20E-07
gene-Led03965-sp3	7.386620136	6.52E-45	2.38E-43
gene-Led03331-sp3	4.30914698	4.57E-41	1.45E-39
gene-Led06357-sp3	2.728329347	5.63E-26	8.86E-25
gene-Led05327-sp3	5.530774442	1.50E-45	5.57E-44
gene-Led09374-sp3	8.407407935	1.63E-34	3.99E-33
gene-Led06463-sp3	-1.616685728	0.000378673	0.000989528
gene-Led05104-sp3	2.135283824	3.54E-16	3.21E-15
gene-Led00661-sp3	-1.575432221	3.02E-42	1.01E-40
gene-Led00884-sp3	1.184586807	3.66E-16	3.31E-15
gene-Led07263-sp3	1.558170311	0.015312367	0.029369144
gene-Led05210-sp3	3.935912954	1.89E-23	2.65E-22
gene-Led07309-sp3	2.775847553	2.41E-40	7.50E-39
gene-Led02929-sp3	1.899999507	1.23E-12	8.21E-12
gene-Led10082-sp3	-1.118231585	7.25E-11	4.18E-10
gene-Led01806-sp3	2.775313633	2.37E-58	1.44E-56
gene-Led05708-sp3	1.325980148	1.39E-16	1.29E-15
gene-Led08679-sp3	0.57938621	9.26E-06	3.05E-05
gene-Led08306-sp3	0.911017305	2.00E-07	8.19E-07
gene-Led01271-sp3	1.65005636	1.98E-07	8.11E-07
gene-Led07193-sp3	5.056528718	2.45E-71	2.33E-69
gene-Led05336-sp3	0.892335013	4.07E-05	0.000122176
gene-Led08477-sp3	2.434891584	2.64E-29	5.01E-28
gene-Led07999-sp3	-1.119713068	0.008578793	0.017378751
gene-Led08562-sp3	1.866068027	2.33E-07	9.43E-07
gene-Led03548-sp3	2.777445656	2.73E-15	2.28E-14
gene-Led06489-sp3	1.853270102	2.21E-05	6.85E-05
gene-Led02627-sp3	1.111502085	5.01E-08	2.19E-07
gene-Led09414-sp3	1.380149211	0.000820205	0.002021472
gene-Led02712-sp3	1.247708268	1.64E-05	5.20E-05
gene-Led07726-sp3	1.112641246	4.27E-06	1.47E-05
gene-Led04786-sp3	1.493947239	0.002406727	0.00544402
gene-Led06016-sp3	1.982922216	2.21E-23	3.08E-22
gene-Led06101-sp3	4.429674727	7.52E-18	7.70E-17
gene-Led09614-sp3	3.412063082	1.58E-34	3.87E-33
gene-Led03853-sp3	2.88993946	2.32E-11	1.40E-10

gene-Led01350-sp3	2.467134377	3.64E-19	4.09E-18
gene-Led00114-sp3	1.289878126	9.94E-06	3.26E-05
gene-Led09148-sp3	2.185356184	6.35E-11	3.68E-10
gene-Led10885-sp3	1.059109461	4.16E-08	1.84E-07
gene-Led06110-sp3	3.754397173	1.71E-27	2.93E-26
gene-Led05858-sp3	2.623773383	2.49E-11	1.50E-10
gene-Led09840-sp3	4.361110249	1.29E-29	2.52E-28
gene-Led03700-sp3	0.826113871	0.006509339	0.013513092
gene-Led10545-sp3	8.118814647	9.12E-50	4.01E-48
gene-Led05448-sp3	2.004841734	3.19E-06	1.12E-05
gene-Led04296-sp3	1.293938083	0.012729628	0.024861087
gene-Led06907-sp3	2.719330524	1.89E-22	2.53E-21
gene-Led02241-sp3	6.8061941	1.33E-32	2.93E-31
gene-Led04404-sp3	2.035758691	4.43E-15	3.64E-14
gene-Led10905-sp3	2.871384499	2.29E-13	1.63E-12
gene-Led08617-sp3	1.549183407	1.13E-09	5.84E-09
gene-Led02457-sp3	3.844519108	4.25E-56	2.39E-54
gene-Led11360-sp3	2.056098967	5.75E-06	1.95E-05
gene-Led09869-sp3	0.541768396	1.90E-06	6.83E-06
gene-Led08115-sp3	1.101533807	1.72E-10	9.61E-10
gene-Led02291-sp3	4.713508704	7.78E-21	9.56E-20
gene-Led05829-sp3	1.76810999	2.72E-11	1.63E-10
gene-Led05403-sp3	1.431372146	5.85E-12	3.73E-11
gene-Led06421-sp3	2.066707013	2.59E-13	1.84E-12
gene-Led06290-sp3	3.448678662	1.12E-10	6.33E-10
gene-Led01739-sp3	5.096111613	1.58E-17	1.58E-16
gene-Led04706-sp3	7.508528659	2.27E-24	3.34E-23
gene-Led05457-sp3	-0.67730427	0.010585073	0.02100309
gene-Led02518-sp3	4.459174048	3.27E-33	7.39E-32
gene-Led03141-sp3	2.596237153	3.22E-15	2.68E-14
gene-Led08151-sp3	1.455065435	6.08E-12	3.87E-11
gene-Led09177-sp3	5.93196834	9.76E-20	1.13E-18
gene-Led03815-sp3	1.248850735	0.002973194	0.006610165
gene-Led00974-sp3	3.513668247	9.86E-29	1.80E-27
gene-Led03657-sp3	2.835283588	2.05E-14	1.59E-13
gene-Led07719-sp3	2.044276485	3.71E-14	2.81E-13
gene-Led04609-sp3	1.422619844	5.34E-09	2.58E-08
gene-Led09508-sp3	4.563109222	4.67E-53	2.29E-51
gene-Led03355-sp3	1.244241912	0.014231762	0.027516928
gene-Led00416-sp3	1.297685621	1.06E-05	3.44E-05
gene-Led07976-sp3	2.131262748	3.75E-61	2.46E-59
gene-Led09284-sp3	1.839145918	1.80E-15	1.53E-14

gene-Led11386-sp3	3.399418099	1.14E-10	6.42E-10
gene-Led05101-sp3	0.897440521	3.63E-06	1.26E-05
gene-Led08565-sp3	0.991143537	9.61E-06	3.16E-05
gene-Led01865-sp3	5.010335532	3.80E-49	1.62E-47
gene-Led06269-sp3	2.742140929	7.20E-11	4.16E-10
gene-Led02165-sp3	1.500232828	0.000473706	0.001220113
gene-Led10829-sp3	2.162653698	5.07E-08	2.22E-07
gene-Led06697-sp3	3.002476273	1.87E-15	1.59E-14
gene-Led08574-sp3	1.571317689	8.22E-17	7.78E-16
gene-Led02845-sp3	4.488352845	1.83E-39	5.55E-38
gene-Led04381-sp3	1.311365358	1.37E-08	6.41E-08
gene-Led02299-sp3	1.965790119	4.87E-05	0.000145086
gene-Led06856-sp3	3.515864489	1.33E-60	8.55E-59
gene-Led05333-sp3	3.94385171	4.11E-63	2.89E-61
gene-Led07521-sp3	1.917531476	0.004271571	0.009174347
gene-Led06847-sp3	-2.227251582	2.05E-08	9.38E-08
gene-Led05965-sp3	2.304323885	9.89E-22	1.28E-20
gene-Led02800-sp3	0.8769316	0.008548585	0.017324534
gene-Led11052-sp3	2.141386819	2.19E-10	1.21E-09
gene-Led00038-sp3	5.85323886	5.29E-25	7.93E-24
gene-Led00149-sp3	1.106239168	5.68E-08	2.47E-07
gene-Led02524-sp3	0.804059562	0.001861337	0.004292898
gene-Led01516-sp3	1.944474274	1.03E-16	9.75E-16
gene-Led06888-sp3	4.701320187	5.32E-13	3.66E-12
gene-Led02756-sp3	1.056670514	4.40E-05	0.000131665
gene-Led03908-sp3	9.080991131	1.19E-101	2.25E-99
gene-Led02067-sp3	0.938690967	7.82E-06	2.60E-05
gene-Led04055-sp3	4.34668902	2.56E-19	2.92E-18
gene-Led05193-sp3	1.405871084	5.41E-05	0.000160182
gene-Led09843-sp3	2.124670303	3.19E-11	1.90E-10
gene-Led04814-sp3	6.109918572	1.14E-53	5.80E-52
gene-Led05422-sp3	1.956384726	1.14E-21	1.47E-20
gene-Led03434-sp3	8.241255806	7.81E-56	4.34E-54
gene-Led01020-sp3	3.83454974	5.71E-61	3.70E-59
gene-Led08436-sp3	-1.041959857	1.21E-22	1.63E-21
gene-Led03683-sp3	2.159123679	1.77E-15	1.51E-14
gene-Led02769-sp3	1.284650925	1.35E-06	4.94E-06
gene-Led01664-sp3	1.179931213	1.41E-12	9.39E-12
gene-Led01229-sp3	5.883619397	3.54E-28	6.31E-27
gene-Led11212-sp3	3.18507808	2.60E-15	2.18E-14
gene-Led05632-sp3	3.810489388	6.47E-32	1.38E-30
gene-Led05720-sp3	1.650113271	4.43E-15	3.64E-14

gene-Led07813-sp3	3.511017098	1.31E-52	6.27E-51
gene-Led08107-sp3	1.550132414	9.26E-07	3.46E-06
gene-Led06084-sp3	7.97672387	9.18E-29	1.69E-27
gene-Led05456-sp3	0.638129969	0.004905281	0.010402132
gene-Led03622-sp3	5.201528562	5.01E-30	9.94E-29
gene-Led04680-sp3	2.372282359	1.72E-10	9.61E-10
gene-Led06728-sp3	5.153370931	1.66E-51	7.58E-50
gene-Led07090-sp3	3.644271439	2.43E-68	2.03E-66
gene-Led08186-sp3	2.632813395	4.47E-21	5.61E-20
gene-Led06361-sp3	0.879147354	4.77E-07	1.85E-06
gene-Led07558-sp3	1.336165002	4.96E-06	1.70E-05
gene-Led00777-sp3	1.508652465	2.08E-07	8.50E-07
gene-Led08839-sp3	1.174679455	3.64E-10	1.98E-09
gene-Led10035-sp3	1.335355796	2.94E-08	1.33E-07
gene-Led00901-sp3	2.619219132	8.60E-29	1.59E-27
gene-Led04061-sp3	3.409058237	2.20E-16	2.02E-15
gene-Led09805-sp3	1.159118135	1.55E-09	7.89E-09
gene-Led05184-sp3	1.853986882	6.00E-08	2.60E-07
gene-Led06453-sp3	1.983661143	6.28E-10	3.31E-09
gene-Led05860-sp3	2.40946299	7.24E-12	4.58E-11
gene-Led09305-sp3	1.525435092	2.87E-11	1.73E-10
gene-Led05531-sp3	1.09721546	0.000108384	0.000308818
gene-Led01612-sp3	1.12044252	8.48E-06	2.80E-05
gene-Led09104-sp3	0.823633181	6.33E-07	2.42E-06
gene-Led08152-sp3	3.275592002	8.49E-36	2.21E-34
gene-Led07502-sp3	-1.117196819	0.023198156	0.042562396
gene-Led05886-sp3	6.00432371	1.07E-22	1.44E-21
gene-Led09665-sp3	0.992098505	3.82E-07	1.50E-06
gene-Led08168-sp3	3.555726044	1.70E-29	3.27E-28
gene-Led09363-sp3	3.17749374	1.79E-64	1.31E-62
gene-Led07975-sp3	1.02770778	1.22E-17	1.23E-16
gene-Led00716-sp3	1.445493158	3.82E-11	2.26E-10
gene-Led06023-sp3	1.86619314	0.008755606	0.017715528
gene-Led01439-sp3	1.773357534	0.000170794	0.000473759
gene-Led00216-sp3	4.162672903	1.18E-82	1.44E-80
gene-Led10636-sp3	2.774227865	6.00E-36	1.59E-34
gene-Led09591-sp3	1.091225246	9.41E-11	5.37E-10
gene-Led06093-sp3	1.160316953	0.022638621	0.041626919
gene-Led06953-sp3	1.510802341	2.28E-20	2.72E-19
gene-Led01713-sp3	1.067992235	1.43E-08	6.65E-08
gene-Led06553-sp3	1.129886773	0.000553232	0.00140693
gene-Led09381-sp3	2.098247934	5.17E-11	3.02E-10

gene-Led04437-sp3	2.314398566	9.27E-13	6.25E-12
gene-Led05557-sp3	1.463676581	0.001339558	0.003171796
gene-Led00743-sp3	0.703661056	1.54E-06	5.60E-06
gene-Led03876-sp3	3.748194073	1.95E-79	2.25E-77
gene-Led08341-sp3	3.337580632	4.23E-35	1.06E-33
gene-Led07200-sp3	2.345524917	6.13E-23	8.38E-22
gene-Led09231-sp3	0.779738881	1.07E-05	3.47E-05
gene-Led10606-sp3	4.036472943	2.41E-26	3.91E-25
gene-Led09055-sp3	2.156151258	5.72E-11	3.33E-10
gene-Led08479-sp3	6.070309634	3.53E-70	3.15E-68
gene-Led10115-sp3	2.623908148	1.48E-06	5.39E-06
gene-Led08708-sp3	0.92317954	2.46E-09	1.23E-08
gene-Led05868-sp3	0.904479363	3.26E-05	9.94E-05
gene-Led05794-sp3	0.683918422	0.022219287	0.040930694
gene-Led00020-sp3	4.608176357	1.06E-14	8.41E-14
gene-Led03533-sp3	0.943357698	0.024437269	0.044562172
gene-Led00035-sp3	1.743664943	0.012447849	0.024358068
gene-Led02410-sp3	3.65753923	3.81E-40	1.18E-38
gene-Led04013-sp3	1.819050313	4.30E-06	1.48E-05
gene-Led05264-sp3	1.066799538	0.005354525	0.01128343
gene-Led06272-sp3	1.52192135	1.28E-31	2.70E-30
gene-Led01932-sp3	3.730809542	2.15E-56	1.23E-54
gene-Led00322-sp3	3.825614614	8.80E-25	1.31E-23
gene-Led08847-sp3	0.824148674	0.00505511	0.010704063
gene-Led00204-sp3	5.141981221	3.43E-27	5.80E-26
gene-Led05462-sp3	1.961322264	0.001304601	0.003095465
gene-Led07733-sp3	1.99357277	9.10E-09	4.32E-08
gene-Led09752-sp3	1.198319171	1.92E-05	6.02E-05
gene-Led00283-sp3	1.225900921	2.32E-08	1.06E-07
gene-Led06506-sp3	4.208964482	1.54E-29	2.98E-28
gene-Led04048-sp3	1.570961013	0.003904179	0.008439335
gene-Led11249-sp3	1.018547295	0.007459235	0.015283151
gene-Led03130-sp3	-1.284585421	2.87E-07	1.15E-06
gene-Led07920-sp3	1.260254591	0.002857935	0.006379296
gene-Led07170-sp3	5.615172133	1.62E-49	7.02E-48
gene-Led05131-sp3	1.785362847	1.10E-07	4.64E-07
gene-Led02284-sp3	0.762665278	0.022383407	0.041202838
gene-Led07386-sp3	1.335732154	0.001568981	0.003669955
gene-Led06112-sp3	2.335294762	1.35E-27	2.32E-26
gene-Led01048-sp3	1.415090044	1.33E-14	1.04E-13
gene-Led10547-sp3	6.163487926	1.29E-35	3.32E-34
gene-Led06227-sp3	4.525112997	5.58E-42	1.83E-40

gene-Led07019-sp3	1.686558881	3.86E-11	2.28E-10
gene-Led00618-sp3	2.623726041	1.37E-28	2.48E-27
gene-Led06378-sp3	5.511523939	1.27E-21	1.64E-20
gene-Led00384-sp3	0.951429971	0.022546869	0.041473374
gene-Led01007-sp3	4.139908542	1.25E-66	9.90E-65
gene-Led00805-sp3	2.11018569	5.44E-66	4.12E-64
gene-Led04834-sp3	2.823840273	1.38E-07	5.78E-07
gene-Led08480-sp3	4.020956129	1.27E-11	7.90E-11
gene-Led10363-sp3	7.601879795	1.16E-35	3.01E-34
gene-Led00571-sp3	3.918382315	4.77E-24	6.85E-23
gene-Led03096-sp3	4.567987043	1.52E-50	6.76E-49
gene-Led09491-sp3	1.400734853	3.02E-08	1.36E-07
gene-Led08554-sp3	2.046029908	6.33E-15	5.14E-14
gene-Led06109-sp3	2.952077813	1.68E-15	1.45E-14
gene-Led00242-sp3	1.172359491	2.70E-05	8.31E-05
gene-Led01854-sp3	2.426639009	5.28E-06	1.80E-05
gene-Led09607-sp3	3.606722858	1.94E-23	2.71E-22
gene-Led06651-sp3	2.440623139	4.19E-13	2.90E-12
gene-Led09378-sp3	5.085128347	2.27E-100	4.07E-98
gene-Led01294-sp3	3.954473575	4.55E-96	7.62E-94
gene-Led00049-sp3	5.314772926	1.44E-19	1.66E-18
gene-Led01970-sp3	4.375994568	4.39E-18	4.57E-17
gene-Led09035-sp3	3.806639845	7.69E-37	2.11E-35
gene-Led03636-sp3	2.532073736	5.22E-26	8.25E-25
gene-Led10023-sp3	3.575874867	1.15E-40	3.62E-39
gene-Led03153-sp3	0.961986634	0.006221351	0.01298762
gene-Led04212-sp3	2.50855247	0.004541742	0.009688407
gene-Led06099-sp3	3.981387122	9.14E-27	1.52E-25
gene-Led01196-sp3	5.884845968	1.27E-84	1.66E-82
gene-Led03547-sp3	1.621972368	3.30E-19	3.72E-18
gene-Led00055-sp3	6.138926223	8.13E-25	1.21E-23
gene-Led06784-sp3	0.981977352	1.05E-21	1.36E-20
gene-Led09867-sp3	1.345511274	1.53E-07	6.36E-07
gene-Led01570-sp3	2.905540465	1.11E-45	4.13E-44
gene-Led03651-sp3	5.813944674	4.86E-80	5.75E-78
gene-Led01463-sp3	1.063210849	0.001344426	0.003182452
gene-Led10103-sp3	2.794164837	4.27E-11	2.52E-10
gene-Led05326-sp3	0.462516595	0.01080146	0.0213742
gene-Led09458-sp3	1.251217054	0.011441789	0.022529661
gene-Led04603-sp3	2.284530559	0.003463565	0.00759628
gene-Led07253-sp3	1.0522165	7.08E-05	0.000206679
gene-Led02341-sp3	2.18925838	8.54E-18	8.70E-17

gene-Led10283-sp3	4.0102561	2.03E-25	3.10E-24
gene-Led06041-sp3	1.75994929	0.00225806	0.0051372
gene-Led01472-sp3	2.314941188	1.94E-23	2.71E-22
gene-Led01146-sp3	-0.998282421	0.014895895	0.028630406
gene-Led10185-sp3	6.695014814	3.08E-106	6.59E-104
gene-Led07898-sp3	2.472290744	7.12E-30	1.41E-28
gene-Led06571-sp3	1.159745054	0.001276739	0.003032933
gene-Led08148-sp3	3.019433653	2.40E-33	5.47E-32
gene-Led04701-sp3	1.020378492	0.000928425	0.002263781
gene-Led02139-sp3	2.622439796	2.67E-21	3.38E-20
gene-Led10887-sp3	1.720236937	0.016148882	0.030814923
gene-Led05522-sp3	2.789464052	1.70E-15	1.46E-14
gene-Led06302-sp3	6.838242925	6.85E-36	1.80E-34
gene-Led11422-sp3	2.261288504	3.47E-25	5.25E-24
gene-Led07482-sp3	1.088814143	0.020582415	0.038265792
gene-Led03435-sp3	0.783528597	8.52E-05	0.000245759
gene-Led02641-sp3	2.159489739	3.10E-27	5.25E-26
gene-Led10652-sp3	4.258069259	5.19E-71	4.79E-69
gene-Led03532-sp3	1.643981777	3.16E-10	1.72E-09
gene-Led09422-sp3	2.990431385	1.65E-33	3.79E-32
gene-Led08008-sp3	-2.259615616	9.24E-09	4.38E-08
gene-Led10332-sp3	2.59848699	1.76E-33	4.02E-32
gene-Led03550-sp3	3.750341784	5.39E-29	1.00E-27
gene-Led00052-sp3	5.753935393	1.06E-37	3.00E-36
gene-Led06836-sp3	2.456080133	2.10E-43	7.38E-42
gene-Led01556-sp3	2.071450354	3.02E-11	1.81E-10
gene-Led02321-sp3	1.202061233	5.18E-10	2.76E-09
gene-Led03852-sp3	2.291211628	1.09E-18	1.19E-17
gene-Led07056-sp3	5.307571394	7.46E-35	1.85E-33
gene-Led01837-sp3	3.970930937	6.24E-49	2.64E-47
gene-Led07543-sp3	2.621804232	8.42E-19	9.26E-18
gene-Led02517-sp3	0.976186627	0.016402289	0.031239202
gene-Led11358-sp3	6.036606068	1.82E-28	3.29E-27
gene-Led10810-sp3	1.123742557	0.024256315	0.044269646
gene-Led02846-sp3	1.573349897	1.11E-06	4.11E-06
gene-Led00099-sp3	1.495620349	0.021208747	0.039285004
gene-Led08610-sp3	2.434511095	0.000228805	0.000621475
gene-Led02934-sp3	1.077077764	9.48E-15	7.60E-14
gene-Led00888-sp3	1.026766906	1.98E-17	1.97E-16
gene-Led10537-sp3	1.331601963	5.39E-10	2.86E-09
gene-Led01955-sp3	4.857809477	9.58E-11	5.45E-10
gene-Led05186-sp3	2.043827325	2.44E-15	2.05E-14

gene-Led00016-sp3	2.121391301	1.74E-08	8.04E-08
gene-Led02749-sp3	2.259062268	2.42E-18	2.56E-17
gene-Led09307-sp3	1.129030111	0.00017671	0.000488686
gene-Led01351-sp3	2.007981723	0.001131577	0.002716973
gene-Led10208-sp3	-1.382542131	3.56E-05	0.000107907
gene-Led00973-sp3	3.477165483	9.85E-27	1.63E-25
gene-Led07319-sp3	2.804464122	1.79E-21	2.28E-20
gene-Led07833-sp3	2.458668928	2.50E-13	1.78E-12
gene-Led06223-sp3	2.214944728	2.35E-43	8.17E-42
gene-Led06652-sp3	0.729814028	0.016316806	0.031105845
gene-Led09149-sp3	0.804498406	1.58E-05	5.02E-05
gene-Led05945-sp3	2.069982721	1.78E-14	1.38E-13
gene-Led10604-sp3	1.243354486	1.71E-15	1.46E-14
gene-Led06320-sp3	1.34201285	6.27E-15	5.09E-14
gene-Led05229-sp3	2.606155772	4.28E-21	5.39E-20
gene-Led06722-sp3	3.534050922	1.16E-17	1.17E-16
gene-Led03242-sp3	3.908165748	1.40E-26	2.29E-25
gene-Led08815-sp3	6.409298325	1.93E-22	2.57E-21
gene-Led00982-sp3	5.3394198	1.54E-25	2.37E-24
gene-Led06214-sp3	3.822357883	1.67E-34	4.07E-33
gene-Led02272-sp3	1.476820604	0.008474819	0.017192361
gene-Led03650-sp3	2.17571397	4.42E-16	3.97E-15
gene-Led00741-sp3	6.696057296	4.89E-194	4.10E-191
gene-Led05827-sp3	3.007302994	4.64E-14	3.50E-13
gene-Led09410-sp3	3.055251015	5.61E-41	1.77E-39
gene-Led06420-sp3	2.531643559	6.36E-12	4.03E-11
gene-Led04704-sp3	3.580438878	2.03E-20	2.43E-19
gene-Led09953-sp3	1.344285325	0.020453687	0.038057868
gene-Led04380-sp3	2.162226613	8.23E-22	1.07E-20
gene-Led08171-sp3	1.577883303	4.13E-07	1.62E-06
gene-Led05247-sp3	4.078515714	8.02E-34	1.88E-32
gene-Led08566-sp3	2.142748853	0.00016866	0.000468744
gene-Led08930-sp3	5.262780548	3.25E-34	7.78E-33
gene-Led01934-sp3	1.208576806	1.11E-11	6.91E-11
gene-Led06042-sp3	1.198402168	1.32E-12	8.82E-12
gene-Led10511-sp3	1.458331423	1.61E-11	9.85E-11
gene-Led07311-sp3	5.30882217	1.78E-57	1.05E-55
gene-Led05449-sp3	0.978255683	0.000690509	0.001722078
gene-Led00022-sp3	2.152566233	4.29E-17	4.12E-16
gene-Led10582-sp3	-1.208141521	1.17E-17	1.18E-16
gene-Led10186-sp3	2.674992304	4.52E-61	2.95E-59
gene-Led06848-sp3	5.040700623	2.63E-31	5.46E-30

gene-Led08149-sp3	3.969906213	1.72E-29	3.30E-28
gene-Led07784-sp3	3.25941888	3.16E-24	4.57E-23
gene-Led06989-sp3	2.199192848	6.49E-16	5.74E-15
gene-Led00091-sp3	3.936621296	1.11E-17	1.12E-16
gene-Led10358-sp3	1.444382383	3.91E-07	1.54E-06
gene-Led04558-sp3	2.483990501	2.87E-08	1.30E-07
gene-Led00880-sp3	2.59045768	7.71E-28	1.34E-26
gene-Led05208-sp3	1.947735753	1.03E-42	3.49E-41
gene-Led01919-sp3	1.075600838	1.54E-06	5.60E-06
gene-Led01145-sp3	4.97886061	3.31E-77	3.69E-75
gene-Led03133-sp3	2.386533883	7.23E-24	1.03E-22
gene-Led02268-sp3	1.412598685	5.17E-11	3.02E-10
gene-Led00871-sp3	0.901156951	0.003769241	0.008186358
gene-Led04154-sp3	1.367778881	9.33E-16	8.15E-15
gene-Led09977-sp3	5.054665918	2.89E-29	5.47E-28
gene-Led10063-sp3	2.556334094	1.07E-14	8.48E-14
gene-Led10619-sp3	1.409059061	1.02E-17	1.04E-16
gene-Led07652-sp3	0.484909184	0.000220654	0.000601447
gene-Led10047-sp3	3.900969135	1.00E-43	3.60E-42
gene-Led04379-sp3	1.155788366	0.000616086	0.001553809
gene-Led01656-sp3	2.839759821	4.62E-48	1.89E-46
gene-Led06335-sp3	1.630959366	8.61E-05	0.000248129
gene-Led01084-sp3	5.556235904	1.36E-54	7.20E-53
gene-Led08710-sp3	0.539337258	0.000267691	0.000718366
gene-Led09745-sp3	2.189570818	6.45E-21	7.94E-20
gene-Led01913-sp3	2.000550213	6.88E-15	5.56E-14
gene-Led00040-sp3	3.305531394	8.53E-13	5.77E-12
gene-Led08845-sp3	-2.240552547	1.08E-17	1.09E-16
gene-Led07316-sp3	5.369014257	3.01E-93	4.88E-91
gene-Led07886-sp3	0.974853383	0.02529292	0.045853675
gene-Led04388-sp3	6.46810033	9.78E-63	6.69E-61
gene-Led09425-sp3	3.091306041	5.95E-33	1.34E-31
gene-Led00206-sp3	1.941150315	0.000724754	0.001799451
gene-Led00031-sp3	4.422231756	8.44E-18	8.60E-17
gene-Led10117-sp3	2.150229905	5.95E-12	3.79E-11
gene-Led10151-sp3	0.703448114	0.001921888	0.004419375
gene-Led07208-sp3	1.488834483	5.63E-21	6.99E-20
gene-Led11211-sp3	1.652506845	3.10E-17	3.03E-16
gene-Led00941-sp3	0.64162905	0.002479758	0.005593498
gene-Led09853-sp3	0.701485827	1.15E-06	4.26E-06
gene-Led00456-sp3	1.038854065	3.78E-06	1.31E-05
gene-Led03799-sp3	4.458560432	9.64E-69	8.22E-67

gene-Led06081-sp3	5.559219372	1.68E-54	8.87E-53
gene-Led02502-sp3	1.690296656	6.79E-45	2.46E-43
gene-Led04190-sp3	0.771504218	0.004519806	0.009647753
gene-Led00085-sp3	1.178443745	1.32E-10	7.42E-10
gene-Led00215-sp3	0.667606129	0.00025159	0.000677146
gene-Led03110-sp3	1.08293362	0.006551072	0.013591312
gene-Led07005-sp3	5.869779569	6.45E-19	7.13E-18
gene-Led10133-sp3	0.699241968	0.020619672	0.038327973
gene-Led01465-sp3	5.358335387	4.64E-33	1.04E-31
gene-Led08570-sp3	4.067901548	2.86E-32	6.22E-31
gene-Led03526-sp3	3.310819546	5.29E-11	3.09E-10
gene-Led03671-sp3	-1.635393209	4.86E-07	1.89E-06
gene-Led10349-sp3	1.253234187	5.82E-12	3.71E-11
gene-Led10694-sp3	1.095285488	2.66E-07	1.07E-06
gene-Led07790-sp3	1.326804411	5.64E-13	3.88E-12
gene-Led10622-sp3	2.102190192	1.32E-16	1.24E-15
gene-Led07789-sp3	0.816351863	0.006422641	0.013352402
gene-Led01794-sp3	3.553772706	4.03E-27	6.79E-26
gene-Led11317-sp3	4.123860984	8.61E-71	7.73E-69
gene-Led08225-sp3	2.925688602	9.85E-51	4.40E-49
gene-Led05872-sp3	4.432807128	2.17E-17	2.15E-16
gene-Led00688-sp3	-1.179104222	9.79E-15	7.83E-14
gene-Led07246-sp3	6.979316141	2.25E-28	4.03E-27
gene-Led05969-sp3	1.758444161	1.30E-13	9.48E-13
gene-Led03973-sp3	0.691037642	9.74E-07	3.64E-06
gene-Led07249-sp3	6.761476471	1.71E-27	2.93E-26
gene-Led08718-sp3	4.288697514	4.24E-15	3.49E-14
gene-Led03564-sp3	1.727957286	0.000447372	0.001156429
gene-Led07014-sp3	0.803269529	0.026519358	0.047870011
gene-Led05476-sp3	1.399663599	1.64E-15	1.41E-14
gene-Led02184-sp3	0.872519653	1.62E-06	5.89E-06
gene-Led01107-sp3	0.621240254	0.00240087	0.005433833
gene-Led02435-sp3	2.166934148	8.68E-13	5.86E-12
gene-Led04605-sp3	6.01787227	6.54E-84	8.33E-82
gene-Led01707-sp3	-1.702337186	1.18E-10	6.66E-10
gene-Led00396-sp3	1.194958976	1.42E-08	6.62E-08
gene-Led01198-sp3	1.544904782	3.91E-07	1.54E-06
gene-Led01791-sp3	1.398518028	9.79E-15	7.83E-14
gene-Led08974-sp3	1.517137927	1.70E-14	1.33E-13
gene-Led10174-sp3	1.140836681	7.80E-14	5.79E-13
gene-Led00698-sp3	2.190148346	2.28E-46	8.74E-45
gene-Led05505-sp3	0.710771618	3.25E-10	1.77E-09

gene-Led07664-sp3	1.158958077	0.002132277	0.004871977
gene-Led03688-sp3	2.635680785	1.03E-12	6.92E-12
gene-Led03075-sp3	-1.876823445	1.02E-17	1.03E-16
gene-Led09830-sp3	3.480064306	1.04E-13	7.67E-13
gene-Led04972-sp3	5.460470567	1.26E-09	6.48E-09
gene-Led02743-sp3	3.98055537	7.98E-33	1.78E-31
gene-Led11005-sp3	1.282665478	2.19E-05	6.81E-05
gene-Led10012-sp3	0.81535471	0.002858871	0.006379969
gene-Led04157-sp3	3.454290094	5.85E-19	6.49E-18
gene-Led04827-sp3	4.42923011	1.91E-10	1.06E-09
gene-Led09167-sp3	2.629700993	2.99E-66	2.28E-64
gene-Led09056-sp3	0.993076508	1.06E-07	4.49E-07
gene-Led03479-sp3	7.652868353	5.54E-214	1.11E-210
gene-Led08578-sp3	1.946558912	3.19E-14	2.43E-13
gene-Led02039-sp3	3.677004053	7.20E-52	3.31E-50
gene-Led06675-sp3	2.377711171	4.35E-20	5.14E-19
gene-Led08607-sp3	6.15704572	7.10E-24	1.01E-22
gene-Led04870-sp3	2.834913988	1.55E-09	7.92E-09
gene-Led04129-sp3	2.604608458	5.23E-11	3.05E-10
gene-Led01201-sp3	3.598099936	3.75E-26	6.01E-25
gene-Led09684-sp3	1.981721738	1.57E-18	1.69E-17
gene-Led03549-sp3	4.076190927	1.41E-63	1.01E-61
gene-Led07410-sp3	6.554425793	1.47E-58	8.95E-57
gene-Led10901-sp3	1.864097302	6.10E-11	3.55E-10
gene-Led01261-sp3	4.020207864	5.25E-72	5.07E-70
gene-Led00291-sp3	1.974371384	2.05E-13	1.47E-12
gene-Led07198-sp3	2.538054649	5.98E-06	2.02E-05
gene-Led00018-sp3	3.533723257	4.39E-25	6.59E-24
gene-Led09818-sp3	1.940908294	1.70E-36	4.59E-35
gene-Led02384-sp3	0.999065356	1.77E-10	9.86E-10
gene-Led05947-sp3	0.852892204	0.000187499	0.000516676
gene-Led03998-sp3	1.306918477	8.19E-09	3.90E-08
gene-Led01761-sp3	1.143409592	0.010801855	0.0213742
gene-Led07957-sp3	3.325573774	3.90E-35	9.84E-34
gene-Led04459-sp3	8.993320154	2.53E-69	2.17E-67
gene-Led02402-sp3	0.747301522	0.000131635	0.000370242
gene-Led10053-sp3	2.328474273	2.27E-13	1.63E-12
gene-Led01308-sp3	1.44026017	6.40E-14	4.78E-13
gene-Led10548-sp3	6.983227795	2.24E-37	6.25E-36
gene-Led10904-sp3	1.72395568	0.006308754	0.013150973
gene-Led07308-sp3	2.486964673	4.08E-67	3.28E-65
gene-Led10632-sp3	1.207675561	0.001623558	0.003787048

gene-Led05627-sp3	0.850214071	1.98E-05	6.19E-05
gene-Led00189-sp3	1.348272981	4.27E-10	2.29E-09
gene-Led01121-sp3	9.917392224	2.87E-197	2.89E-194
gene-Led07261-sp3	6.890990182	1.14E-48	4.75E-47
gene-Led03284-sp3	3.056716448	9.70E-25	1.44E-23
gene-Led09507-sp3	1.550007516	3.36E-09	1.66E-08
gene-Led00054-sp3	5.389238481	3.20E-18	3.36E-17
gene-Led06913-sp3	4.377020632	3.34E-15	2.78E-14
gene-Led07817-sp3	3.53231193	2.66E-30	5.34E-29
gene-Led07791-sp3	1.096954671	0.008465553	0.017177029
gene-Led02348-sp3	2.92001406	3.72E-10	2.02E-09
gene-Led00457-sp3	0.71660964	3.65E-07	1.44E-06
gene-Led06712-sp3	1.165107947	0.002324031	0.005272977
gene-Led03696-sp3	1.508452769	7.34E-11	4.22E-10
gene-Led00658-sp3	1.709083364	3.40E-12	2.20E-11
gene-Led01698-sp3	2.045605823	5.83E-23	7.98E-22
gene-Led04337-sp3	0.565618388	4.03E-07	1.58E-06
gene-Led04047-sp3	1.514013891	1.63E-05	5.16E-05
gene-Led09537-sp3	1.85371354	2.36E-08	1.07E-07
gene-Led02647-sp3	5.648657896	9.97E-231	3.34E-227
gene-Led07847-sp3	1.994076766	6.69E-09	3.21E-08
gene-Led01295-sp3	3.300101982	2.79E-20	3.32E-19
gene-Led06694-sp3	1.157341187	0.000901715	0.002203998
gene-Led10364-sp3	2.789837368	3.05E-11	1.82E-10
gene-Led07088-sp3	0.727223988	0.000235975	0.000638707
gene-Led08555-sp3	3.114664733	3.00E-132	9.72E-130
gene-Led09187-sp3	1.135392247	0.000434125	0.001125659
gene-Led00413-sp3	1.929652323	5.38E-10	2.86E-09
gene-Led04711-sp3	7.496131599	4.19E-32	8.97E-31
gene-Led03652-sp3	4.074617669	2.48E-26	4.03E-25
gene-Led00646-sp3	0.868555808	0.000183325	0.000505866
gene-Led07015-sp3	3.7725399	1.92E-35	4.87E-34
gene-Led07524-sp3	2.29585221	1.14E-18	1.24E-17
gene-Led10773-sp3	0.852128842	0.015491396	0.029669875
gene-Led04211-sp3	2.03973619	1.29E-11	8.00E-11
gene-Led03472-sp3	2.459953816	9.50E-23	1.29E-21
gene-Led01871-sp3	1.078685984	0.000339104	0.00089567
gene-Led05908-sp3	1.716168542	3.84E-12	2.48E-11
gene-Led02229-sp3	1.373844869	0.000863006	0.002122276
gene-Led06027-sp3	1.633239322	0.000761667	0.001884123
gene-Led03809-sp3	1.42688922	4.31E-05	0.000129051
gene-Led10856-sp3	2.358605513	6.50E-08	2.81E-07

gene-Led06614-sp3	0.990627237	0.00022141	0.000603018
gene-Led07213-sp3	1.408743226	0.0002453	0.000661987
gene-Led06807-sp3	1.321257324	0.006279712	0.013094809
gene-Led00682-sp3	1.385679523	0.000738916	0.0018319
gene-Led08402-sp3	1.659505873	6.21E-10	3.28E-09
gene-Led03407-sp3	0.787043025	0.006147168	0.012846087
gene-Led03107-sp3	1.102507259	1.97E-10	1.09E-09
gene-Led11023-sp3	5.760747473	9.78E-24	1.39E-22
gene-Led07154-sp3	3.763274492	6.09E-23	8.33E-22
gene-Led07782-sp3	1.396136768	0.000370164	0.000970571
gene-Led03131-sp3	1.485183666	7.75E-08	3.32E-07
gene-Led10513-sp3	1.567836401	7.77E-08	3.33E-07
gene-Led02612-sp3	-0.967490463	3.18E-07	1.26E-06
gene-Led04056-sp3	4.125784486	2.38E-05	7.37E-05
gene-Led09140-sp3	4.788224136	1.45E-110	3.47E-108
gene-Led04284-sp3	0.883674841	0.002677207	0.005995847
gene-Led06022-sp3	1.36088561	1.49E-05	4.75E-05
gene-Led06916-sp3	1.220198929	0.000302411	0.000805523
gene-Led08185-sp3	4.413526014	5.73E-51	2.57E-49
gene-Led00042-sp3	4.33775343	1.39E-35	3.58E-34
gene-Led06344-sp3	1.788020607	1.84E-24	2.70E-23
gene-Led05268-sp3	2.648688366	4.74E-17	4.53E-16
gene-Led03531-sp3	2.13172461	1.51E-19	1.74E-18
gene-Led01494-sp3	2.058273879	3.22E-22	4.25E-21
gene-Led01841-sp3	4.156683908	8.30E-54	4.24E-52
gene-Led03418-sp3	2.13620808	8.86E-42	2.88E-40
gene-Led02126-sp3	1.122306173	0.023973785	0.043817614
gene-Led04003-sp3	0.895115567	0.000156386	0.000435957
gene-Led06860-sp3	1.605133735	1.25E-11	7.74E-11
gene-Led05811-sp3	1.137145456	0.001139626	0.002734993
gene-Led05953-sp3	0.998786674	0.000158602	0.000441522
gene-Led03747-sp3	0.617492554	0.000991627	0.002406802
gene-Led06157-sp3	2.647195969	2.52E-10	1.39E-09
gene-Led09673-sp3	0.914023408	0.023991004	0.043841118
gene-Led04974-sp3	1.863585389	1.74E-34	4.22E-33
gene-Led10975-sp3	0.645998121	0.011227465	0.022133642
gene-Led03258-sp3	2.366643565	1.97E-10	1.09E-09
gene-Led06083-sp3	3.952073595	1.67E-22	2.23E-21
gene-Led03166-sp3	0.699919928	8.18E-06	2.71E-05
gene-Led06362-sp3	4.104319393	3.72E-32	8.02E-31
gene-Led10189-sp3	1.433531598	5.59E-09	2.69E-08
gene-Led08158-sp3	1.561421559	0.000113203	0.000321454

gene-Led00427-sp3	0.949575737	0.012755262	0.024906315
gene-Led10581-sp3	8.889626211	2.54E-68	2.11E-66
gene-Led01915-sp3	0.682740808	8.63E-06	2.85E-05
gene-Led03810-sp3	3.549053849	4.75E-08	2.08E-07
gene-Led04634-sp3	0.855132042	1.64E-08	7.59E-08
gene-Led06952-sp3	4.519695643	2.01E-38	5.83E-37
gene-Led10678-sp3	1.385192952	7.28E-10	3.81E-09
gene-Led05861-sp3	2.685328762	2.48E-56	1.40E-54
gene-Led05903-sp3	1.62860129	2.76E-06	9.75E-06
gene-Led06839-sp3	4.068483996	2.39E-18	2.53E-17
gene-Led05419-sp3	2.080854676	0.004115239	0.0088556
gene-Led05870-sp3	2.072992252	2.93E-09	1.45E-08
gene-Led00013-sp3	1.021760881	1.25E-10	7.03E-10
gene-Led08167-sp3	0.952673415	2.66E-05	8.18E-05
gene-Led05288-sp3	6.57882055	2.50E-48	1.03E-46
gene-Led09448-sp3	-0.697654276	5.40E-06	1.84E-05
gene-Led08843-sp3	1.004701728	8.35E-05	0.00024125
gene-Led07634-sp3	0.798732094	3.01E-05	9.24E-05
gene-Led07231-sp3	1.833646218	0.001394835	0.003290163
gene-Led04136-sp3	3.75441307	5.07E-56	2.83E-54
gene-Led05615-sp3	1.53235143	1.73E-15	1.48E-14
gene-Led06250-sp3	1.541766734	0.000219072	0.000597459
gene-Led04821-sp3	1.039955752	0.000150894	0.000421581
gene-Led09779-sp3	-1.804211238	1.48E-05	4.71E-05
gene-Led09362-sp3	5.22302175	4.15E-92	6.23E-90
gene-Led03306-sp3	1.51619589	0.000107923	0.000307677
gene-Led02754-sp3	0.906757954	7.00E-10	3.67E-09
gene-Led07168-sp3	2.078751493	2.40E-17	2.37E-16
gene-Led06143-sp3	1.968035576	3.26E-17	3.19E-16
gene-Led06794-sp3	3.815806215	5.55E-49	2.35E-47
gene-Led03902-sp3	4.330828744	6.02E-204	7.57E-201
gene-Led03404-sp3	5.26085704	2.92E-29	5.50E-28
gene-Led10540-sp3	2.939657063	5.39E-21	6.71E-20
gene-Led10925-sp3	2.360998013	1.33E-16	1.24E-15
gene-Led00670-sp3	1.476298495	3.52E-05	0.0001068
gene-Led04705-sp3	5.295328999	1.33E-16	1.25E-15
gene-Led09952-sp3	3.226334034	1.09E-21	1.40E-20
gene-Led01891-sp3	1.383990997	1.11E-06	4.11E-06
gene-Led07085-sp3	3.482926554	6.41E-15	5.20E-14
gene-Led00729-sp3	1.981534607	5.28E-16	4.70E-15
gene-Led05828-sp3	1.73694295	3.51E-18	3.68E-17
gene-Led02084-sp3	1.01554773	0.001339609	0.003171796

gene-Led07542-sp3	1.017963529	2.51E-06	8.91E-06
gene-Led00068-sp3	5.636123084	2.26E-54	1.17E-52
gene-Led10605-sp3	5.256459722	1.15E-106	2.58E-104
gene-Led10246-sp3	1.27740927	0.000879021	0.002157441
gene-Led10744-sp3	1.664483789	6.69E-14	4.99E-13
gene-Led08061-sp3	1.169899499	0.012301441	0.024085633
gene-Led07012-sp3	0.94170706	2.59E-10	1.42E-09
gene-Led05420-sp3	2.395949277	2.66E-38	7.68E-37
gene-Led04216-sp3	3.945771418	2.06E-16	1.90E-15
gene-Led04698-sp3	3.96425709	3.28E-19	3.70E-18
gene-Led09715-sp3	0.595371093	0.005177206	0.010939566
gene-Led03649-sp3	1.303594719	2.72E-13	1.92E-12
gene-Led01444-sp3	-1.681211489	0.000292466	0.000781308
gene-Led04885-sp3	4.925009562	6.58E-31	1.35E-29
gene-Led04370-sp3	6.127451273	1.53E-55	8.45E-54
gene-Led05722-sp3	2.600054497	4.10E-17	3.95E-16
gene-Led10557-sp3	3.334300757	7.28E-12	4.60E-11
gene-Led06747-sp3	0.653099185	0.003523279	0.007713788
gene-Led11091-sp3	2.721935982	1.37E-36	3.72E-35
gene-Led01044-sp3	1.390913274	3.04E-14	2.33E-13
gene-Led01574-sp3	4.27860262	1.70E-34	4.15E-33
gene-Led04770-sp3	1.155002889	0.003272792	0.007217219
gene-Led05695-sp3	1.376219468	0.000463067	0.001194237
gene-Led00998-sp3	4.56895024	2.64E-29	5.01E-28
gene-Led02724-sp3	1.482123739	8.72E-08	3.71E-07
gene-Led03496-sp3	1.256266693	0.000176694	0.000488686
gene-Led00446-sp3	-1.633655593	3.44E-10	1.87E-09
gene-Led06985-sp3	7.062269034	3.54E-190	2.74E-187
gene-Led03509-sp3	2.470278994	3.70E-19	4.15E-18
gene-Led06683-sp3	1.737614461	7.37E-06	2.46E-05
gene-Led03953-sp3	3.162568183	1.16E-12	7.80E-12
gene-Led08688-sp3	1.931086502	1.70E-08	7.84E-08
gene-Led01482-sp3	3.208352566	2.18E-11	1.32E-10
gene-Led07333-sp3	-1.180599721	0.001413547	0.003331175
gene-Led03759-sp3	5.759924371	4.09E-57	2.39E-55
gene-Led06210-sp3	0.712944163	1.56E-06	5.68E-06
gene-Led00687-sp3	3.162033934	6.88E-08	2.97E-07
gene-Led00543-sp3	1.085921151	0.000186327	0.000513588
gene-Led01038-sp3	0.458604881	0.009754539	0.019515072
gene-Led11070-sp3	6.076995072	2.30E-52	1.08E-50
gene-Led09268-sp3	0.967729334	0.009774411	0.019548822
gene-Led01938-sp3	0.897081013	1.23E-05	3.96E-05

gene-Led02023-sp3	1.263360647	2.03E-17	2.02E-16
gene-Led09544-sp3	4.710739404	6.28E-13	4.29E-12
gene-Led03080-sp3	1.133178142	0.000197362	0.000541923
gene-Led10460-sp3	2.495193427	1.05E-13	7.72E-13
gene-Led07758-sp3	8.942454519	6.06E-151	2.77E-148
gene-Led02672-sp3	3.495732334	2.48E-23	3.45E-22
gene-Led05581-sp3	1.184876695	0.001268653	0.003015149
gene-Led04695-sp3	1.304779927	0.000570753	0.001445571
gene-Led00648-sp3	5.876347129	1.20E-18	1.31E-17
gene-Led04111-sp3	1.212951429	3.13E-14	2.40E-13
gene-Led01086-sp3	2.506486772	3.58E-52	1.67E-50
gene-Led01404-sp3	-1.506718522	2.70E-05	8.31E-05
gene-Led10656-sp3	1.925502436	7.19E-09	3.44E-08
gene-Led07169-sp3	1.645982706	4.64E-25	6.96E-24
gene-Led02781-sp3	1.443042185	2.56E-13	1.81E-12
gene-Led06372-sp3	0.853720759	0.000555596	0.001411513
gene-Led00924-sp3	1.452227423	0.000141932	0.000397868
gene-Led08588-sp3	1.019822199	0.007387215	0.015157203
gene-Led08212-sp3	1.363129243	0.014076447	0.027242815
gene-Led10653-sp3	1.011666407	2.14E-16	1.97E-15
gene-Led07785-sp3	-1.443498476	0.000224153	0.000609828
gene-Led09218-sp3	1.108791534	0.019754863	0.036938912
gene-Led09351-sp3	1.168196598	2.96E-15	2.47E-14
gene-Led05016-sp3	2.636346379	4.37E-24	6.28E-23
gene-Led09078-sp3	1.004580268	0.005634925	0.011832166
gene-Led06999-sp3	1.664053922	2.09E-14	1.62E-13
gene-Led02171-sp3	1.300897774	2.12E-07	8.64E-07
gene-Led09820-sp3	3.46454555	4.34E-08	1.91E-07
gene-Led07677-sp3	3.366140348	1.58E-11	9.70E-11
gene-Led03727-sp3	1.334440123	4.34E-07	1.70E-06
gene-Led08885-sp3	0.984993952	0.00014712	0.00041138
gene-Led04307-sp3	0.58851908	0.007818503	0.01594778
gene-Led01981-sp3	1.338899376	4.30E-05	0.000128761
gene-Led08813-sp3	-1.07216816	0.000864761	0.002125034
gene-Led10853-sp3	1.845005936	4.91E-12	3.15E-11
gene-Led01609-sp3	0.804234029	1.80E-15	1.54E-14
gene-Led09186-sp3	0.870193429	0.002302925	0.00523217
gene-Led05826-sp3	1.542180598	4.35E-05	0.000130234
gene-Led05076-sp3	0.472886291	0.001046142	0.002529958
gene-Led02915-sp3	2.454108278	5.64E-25	8.45E-24
gene-Led04348-sp3	2.629564229	4.08E-09	2.00E-08
gene-Led01693-sp3	7.571878429	4.89E-177	3.28E-174

gene-Led10351-sp3	1.235362595	3.12E-11	1.86E-10
gene-Led02343-sp3	1.044180293	0.000421936	0.001096314
gene-Led07408-sp3	1.513827186	6.22E-06	2.10E-05
gene-Led07442-sp3	0.835644114	0.003350427	0.007369034
gene-Led09884-sp3	0.404835014	0.001371897	0.003239855
gene-Led09828-sp3	1.945420436	2.79E-14	2.14E-13
gene-Led02250-sp3	-2.933077577	1.43E-15	1.24E-14
gene-Led03560-sp3	1.265510729	1.72E-07	7.10E-07
gene-Led00681-sp3	2.907414378	1.19E-07	5.00E-07
gene-Led07010-sp3	0.745767128	1.83E-06	6.61E-06
gene-Led00108-sp3	1.215764326	9.26E-05	0.000265839
gene-Led03104-sp3	1.562768355	2.86E-09	1.42E-08
gene-Led07468-sp3	1.330919971	0.00323137	0.007135263
gene-Led06345-sp3	2.305230825	1.06E-21	1.37E-20
gene-Led00021-sp3	3.502847633	8.18E-10	4.26E-09
gene-Led02095-sp3	1.491892469	1.06E-12	7.15E-12
gene-Led07063-sp3	0.580213483	0.013488695	0.0262011
gene-Led04297-sp3	1.008699219	0.018009503	0.034023212
gene-Led09138-sp3	-0.678868012	0.026080301	0.047145159
gene-Led06870-sp3	0.6160171	7.31E-06	2.44E-05
gene-Led10325-sp3	1.559792816	4.23E-06	1.45E-05
gene-Led07822-sp3	-1.585131504	3.16E-08	1.42E-07
gene-Led09748-sp3	-1.628231888	1.14E-07	4.82E-07
gene-Led10005-sp3	2.131226477	1.80E-28	3.27E-27
gene-Led04105-sp3	0.587192545	0.003121149	0.006902488
gene-Led08024-sp3	0.617952756	0.001672056	0.003891148
gene-Led08396-sp3	1.069271007	0.000476073	0.001224952
gene-Led07676-sp3	1.564106936	4.30E-10	2.31E-09
gene-Led07926-sp3	0.75482348	0.003058698	0.00677548
gene-Led04258-sp3	0.714856718	0.002891824	0.006442075
gene-Led03144-sp3	1.708394069	1.95E-34	4.72E-33
gene-Led04928-sp3	2.082642286	6.26E-10	3.31E-09
gene-Led02494-sp3	8.922450655	0	0
gene-Led05510-sp3	2.187272431	3.29E-09	1.62E-08
gene-Led09366-sp3	1.318945634	9.74E-06	3.19E-05
gene-Led04267-sp3	1.143413267	7.16E-08	3.08E-07
gene-Led09683-sp3	1.729614516	0.013377613	0.026005417
gene-Led04697-sp3	3.176854586	7.95E-12	5.02E-11
gene-Led07037-sp3	1.21953469	3.10E-05	9.50E-05
gene-Led03820-sp3	-1.998655188	2.70E-16	2.47E-15
gene-Led08119-sp3	0.82179399	0.014269545	0.027579377
gene-Led00130-sp3	1.232594206	0.000958559	0.002330585

gene-Led09565-sp3	4.992128403	9.32E-89	1.32E-86
gene-Led06761-sp3	3.915386974	1.59E-55	8.74E-54
gene-Led00017-sp3	2.130839394	1.59E-06	5.76E-06
gene-Led00781-sp3	0.632009973	0.001995263	0.004577636
gene-Led05189-sp3	1.843336912	4.16E-10	2.24E-09
gene-Led11276-sp3	4.021218986	1.10E-10	6.24E-10
gene-Led03846-sp3	0.551397562	0.013547349	0.026299794
gene-Led03450-sp3	1.059595975	0.007102314	0.014626346
gene-Led01152-sp3	1.384212955	2.90E-25	4.42E-24
gene-Led11285-sp3	2.846969594	1.47E-27	2.53E-26
gene-Led02459-sp3	2.972361132	9.38E-28	1.63E-26
gene-Led08897-sp3	0.9078425	5.29E-07	2.05E-06
gene-Led09538-sp3	2.903724407	2.69E-18	2.83E-17
gene-Led02349-sp3	5.416596654	5.01E-38	1.42E-36
gene-Led08696-sp3	0.442582809	0.022107752	0.040740156
gene-Led02104-sp3	0.61213821	0.000502808	0.001287814
gene-Led06286-sp3	0.703404555	0.002863713	0.00638794
gene-Led11048-sp3	1.259853698	0.000193604	0.000532227
gene-Led05350-sp3	4.095116036	2.61E-51	1.18E-49
gene-Led09204-sp3	0.716041805	4.22E-05	0.000126601
gene-Led07508-sp3	1.965056427	0.000112553	0.000319882
gene-Led10601-sp3	1.717433738	1.83E-08	8.42E-08
gene-Led02065-sp3	1.919655889	1.78E-55	9.76E-54
gene-Led04914-sp3	1.680652177	1.91E-18	2.04E-17
gene-Led02856-sp3	1.129193757	1.41E-19	1.63E-18
gene-Led01831-sp3	0.840287828	0.001490578	0.003496324
gene-Led07368-sp3	4.006947233	3.83E-34	9.12E-33
gene-Led07297-sp3	3.491922642	8.63E-17	8.17E-16
gene-Led05927-sp3	2.030499585	6.63E-23	9.04E-22
gene-Led06613-sp3	2.632999241	1.19E-14	9.43E-14
gene-Led09593-sp3	1.598431645	6.45E-23	8.80E-22
gene-Led02330-sp3	5.634663619	1.13E-28	2.06E-27
gene-Led01468-sp3	3.58795367	1.56E-21	2.01E-20
gene-Led08180-sp3	0.818958317	2.04E-05	6.38E-05
gene-Led10256-sp3	-1.59256027	1.93E-09	9.74E-09
gene-Led09470-sp3	1.374217878	3.58E-08	1.59E-07
gene-Led04186-sp3	1.399659436	1.75E-05	5.52E-05
gene-Led02398-sp3	1.344669693	1.18E-08	5.55E-08
gene-Led07613-sp3	0.940656243	0.00046515	0.001198995
gene-Led08886-sp3	4.642961702	1.88E-49	8.14E-48
gene-Led10854-sp3	2.583828671	3.60E-19	4.05E-18
gene-Led00254-sp3	-0.496540014	0.01046513	0.020793812

gene-Led04488-sp3	2.242100251	1.83E-25	2.81E-24
gene-Led06476-sp3	1.314828107	3.15E-08	1.42E-07
gene-Led02066-sp3	1.592072351	0.001275735	0.003031266
gene-Led08117-sp3	0.654960183	0.022370892	0.041194879
gene-Led00393-sp3	0.949910349	8.65E-11	4.94E-10
gene-Led00850-sp3	5.442759696	6.43E-90	9.24E-88
gene-Led02644-sp3	2.389924467	2.06E-05	6.43E-05
gene-Led06229-sp3	1.072665871	3.09E-16	2.81E-15
gene-Led01483-sp3	2.491230647	3.15E-05	9.62E-05
gene-Led05676-sp3	-3.314396514	1.75E-08	8.09E-08
gene-Led07972-sp3	0.563326122	0.003853205	0.008343496
gene-Led07670-sp3	1.290605437	1.13E-11	7.06E-11
gene-Led05232-sp3	0.826743076	0.008450474	0.017160279
gene-Led07334-sp3	-1.200651897	1.04E-13	7.65E-13
gene-Led05570-sp3	3.186214478	1.34E-13	9.77E-13
gene-Led03429-sp3	1.046960622	0.012835602	0.025053462
gene-Led09475-sp3	0.896199688	0.005251737	0.011083083
gene-Led08012-sp3	1.530803459	2.08E-05	6.49E-05
gene-Led10006-sp3	4.33279782	2.11E-48	8.77E-47
gene-Led05901-sp3	-1.047107423	0.000907385	0.002215902
gene-Led07188-sp3	4.529955426	8.23E-37	2.25E-35
gene-Led06875-sp3	1.180673732	8.85E-07	3.33E-06
gene-Led02780-sp3	1.643864652	5.22E-06	1.78E-05
gene-Led09693-sp3	1.275601207	5.98E-06	2.02E-05
gene-Led03215-sp3	1.413358337	1.69E-12	1.11E-11
gene-Led03430-sp3	1.605423977	4.15E-10	2.24E-09
gene-Led07292-sp3	2.255183777	1.06E-13	7.80E-13
gene-Led01233-sp3	1.018006174	9.01E-05	0.000259195
gene-Led05048-sp3	-0.973696438	0.010740794	0.021274302
gene-Led05250-sp3	1.058288394	1.37E-06	5.01E-06
gene-Led00094-sp3	2.320837707	1.42E-09	7.24E-09
gene-Led05839-sp3	5.501924638	1.28E-66	1.01E-64
gene-Led07781-sp3	-1.794077513	1.35E-11	8.32E-11
gene-Led04585-sp3	4.467174388	4.56E-63	3.19E-61
gene-Led03489-sp3	1.653276692	2.68E-18	2.83E-17
gene-Led07698-sp3	0.912706947	8.02E-08	3.43E-07
gene-Led07369-sp3	1.058806274	4.70E-08	2.06E-07
gene-Led04968-sp3	7.993678179	1.91E-54	9.99E-53
gene-Led03007-sp3	1.301588838	0.00097423	0.002366862
gene-Led04236-sp3	2.38327342	1.80E-31	3.77E-30
gene-Led08686-sp3	6.919645257	2.30E-37	6.42E-36
gene-Led02546-sp3	-0.600452107	0.010579097	0.020999518

gene-Led05188-sp3	0.843401553	0.000113421	0.000321967
gene-Led10724-sp3	0.7461085	0.000907467	0.002215902
gene-Led09827-sp3	1.955512769	1.75E-08	8.07E-08
gene-Led04547-sp3	3.993680524	1.69E-12	1.11E-11
gene-Led02458-sp3	3.59931005	1.48E-52	7.04E-51
gene-Led00111-sp3	0.733418625	0.022759485	0.041833863
gene-Led10992-sp3	5.171199351	1.09E-34	2.70E-33
gene-Led09419-sp3	0.90007458	7.73E-07	2.93E-06
gene-Led07545-sp3	2.327543779	2.39E-11	1.44E-10
gene-Led08011-sp3	2.850878802	2.61E-16	2.39E-15
gene-Led01910-sp3	3.499955554	2.92E-19	3.30E-18
gene-Led01826-sp3	1.356956089	0.000879571	0.002158134
gene-Led11444-sp3	1.227118275	0.004618739	0.009838051
gene-Led11251-sp3	1.101314338	0.000303638	0.00080772
gene-Led08840-sp3	1.268958407	0.003294687	0.007257547
gene-Led03644-sp3	3.19626256	1.06E-11	6.64E-11
gene-Led06859-sp3	0.528720733	9.74E-05	0.000279021
gene-Led10193-sp3	1.447499098	0.00076828	0.001900016
gene-Led05898-sp3	-2.241362011	1.62E-32	3.55E-31
gene-Led08458-sp3	3.502369084	7.65E-39	2.26E-37
gene-Led09451-sp3	0.526116882	0.003838939	0.008317977
gene-Led11260-sp3	1.907021204	8.26E-08	3.53E-07
gene-Led04456-sp3	1.712796602	2.37E-08	1.08E-07
gene-Led03589-sp3	0.876940382	3.76E-08	1.67E-07
gene-Led09570-sp3	0.908334996	0.003945614	0.008523407
gene-Led04358-sp3	1.415948917	0.000813057	0.002006313
gene-Led04311-sp3	1.637858562	1.17E-17	1.18E-16
gene-Led05464-sp3	1.29751406	5.49E-08	2.39E-07
gene-Led02725-sp3	1.21570961	1.30E-07	5.42E-07
gene-Led03333-sp3	1.361848329	0.006277178	0.013093292
gene-Led06931-sp3	4.229135116	1.48E-49	6.45E-48
gene-Led08549-sp3	2.654563483	1.64E-21	2.10E-20
gene-Led02642-sp3	1.595299215	1.56E-31	3.29E-30
gene-Led10119-sp3	-0.692968579	0.007888119	0.016083255
gene-Led08446-sp3	0.911341473	0.000529789	0.001352784
gene-Led10045-sp3	0.921291056	0.001836855	0.004242271
gene-Led01298-sp3	2.171838011	0.000118421	0.000335233
gene-Led07891-sp3	-0.883881123	0.01043769	0.020751588
gene-Led06655-sp3	1.077683449	3.21E-05	9.80E-05
gene-Led06333-sp3	0.843520372	0.007982725	0.016262964
gene-Led02135-sp3	0.94988173	0.002996885	0.006654012
gene-Led03445-sp3	0.669479078	0.000198105	0.000543815

gene-Led03116-sp3	0.592970503	0.001533145	0.003588636
gene-Led07965-sp3	2.200899043	5.09E-11	2.97E-10
gene-Led03083-sp3	1.005514361	2.05E-06	7.35E-06
gene-Led03655-sp3	1.854440877	2.13E-15	1.80E-14
gene-Led05345-sp3	1.815362078	8.92E-16	7.80E-15
gene-Led08023-sp3	1.280831951	4.72E-08	2.07E-07
gene-Led06810-sp3	1.286745155	1.95E-05	6.13E-05
gene-Led05843-sp3	-3.643996469	7.36E-16	6.50E-15
gene-Led05271-sp3	2.082536549	2.76E-09	1.37E-08
gene-Led03785-sp3	1.551622127	1.04E-22	1.41E-21
gene-Led05070-sp3	0.918959511	0.00203125	0.004655951
gene-Led01705-sp3	2.061528575	8.47E-11	4.84E-10
gene-Led07862-sp3	-1.608223087	1.49E-05	4.74E-05
gene-Led06321-sp3	2.659749339	2.31E-43	8.05E-42
gene-Led05503-sp3	0.775197898	1.29E-05	4.16E-05
gene-Led10827-sp3	1.015001663	3.18E-07	1.26E-06
gene-Led09552-sp3	1.041783365	1.12E-08	5.28E-08
gene-Led07438-sp3	1.26283097	2.13E-08	9.75E-08
gene-Led01403-sp3	1.385843436	3.55E-06	1.23E-05
gene-Led10172-sp3	3.853770064	1.85E-45	6.80E-44
gene-Led09315-sp3	1.187466993	1.45E-05	4.61E-05
gene-Led04110-sp3	2.112915777	3.14E-05	9.61E-05
gene-Led03045-sp3	6.66052784	1.95E-31	4.07E-30
gene-Led06102-sp3	2.73844155	2.01E-12	1.32E-11
gene-Led10859-sp3	1.979298567	0.000383327	0.00100065
gene-Led06730-sp3	2.684722042	2.83E-06	9.99E-06
gene-Led03477-sp3	-1.395131705	1.62E-14	1.26E-13
gene-Led01631-sp3	4.519910659	6.70E-150	2.93E-147
gene-Led10916-sp3	4.011577765	3.84E-17	3.71E-16
gene-Led06919-sp3	-0.990421995	0.004315456	0.009258715
gene-Led01727-sp3	-0.77398468	0.000232859	0.000631632
gene-Led02150-sp3	0.891007276	2.32E-14	1.79E-13
gene-Led10046-sp3	0.49793385	0.001234849	0.002942458
gene-Led01552-sp3	3.300112005	3.76E-07	1.48E-06
gene-Led07586-sp3	1.015195095	0.01392328	0.026967138
gene-Led04509-sp3	0.60306375	0.00911106	0.018338811
gene-Led01382-sp3	0.611847222	0.026797599	0.04834529
gene-Led10862-sp3	1.146569844	9.51E-05	0.000272814
gene-Led09987-sp3	0.635197066	0.002184669	0.004980372
gene-Led09298-sp3	1.755748572	1.91E-09	9.65E-09
gene-Led06603-sp3	-2.54952958	6.14E-09	2.95E-08
gene-Led04325-sp3	0.670178479	0.0011198	0.002692553

gene-Led01591-sp3	1.601655435	1.05E-10	5.94E-10
gene-Led07116-sp3	0.938883673	4.69E-10	2.51E-09
gene-Led09033-sp3	1.895442751	2.90E-11	1.74E-10
gene-Led03199-sp3	0.682238947	0.000820128	0.002021472
gene-Led07916-sp3	0.754825718	0.000239813	0.00064805
gene-Led01972-sp3	0.882279405	3.21E-09	1.59E-08
gene-Led06313-sp3	1.472194657	0.002291144	0.005207758
gene-Led05509-sp3	0.49297199	0.020296593	0.037818291
gene-Led05137-sp3	2.23887508	4.92E-09	2.39E-08
gene-Led01531-sp3	1.028436544	0.001790558	0.004142005
gene-Led04991-sp3	-0.580609096	0.000463831	0.001195902
gene-Led05785-sp3	2.188579098	3.47E-15	2.89E-14
gene-Led06829-sp3	1.425878986	5.63E-05	0.000166251
gene-Led07590-sp3	-1.220934176	0.000398608	0.001038921
gene-Led04078-sp3	1.229442945	8.61E-24	1.23E-22
gene-Led04460-sp3	0.959088702	9.26E-06	3.05E-05
gene-Led00067-sp3	0.662887203	0.010974998	0.021674166
gene-Led05412-sp3	0.742103688	0.007515183	0.015382114
gene-Led00295-sp3	1.771716557	4.39E-33	9.91E-32
gene-Led02810-sp3	0.582552202	0.025085684	0.045543647
gene-Led10843-sp3	1.076304826	0.002697367	0.006036963
gene-Led11392-sp3	0.548288132	0.004559219	0.009721566
gene-Led01045-sp3	0.622617081	0.00104182	0.002520162
gene-Led00852-sp3	0.664902937	1.09E-05	3.53E-05
gene-Led05894-sp3	0.377230834	0.024438749	0.044562172
gene-Led02367-sp3	-1.192748986	0.000408654	0.001062903
gene-Led08113-sp3	0.432979788	0.022827781	0.041944067
gene-Led01920-sp3	0.928574683	0.004027765	0.008680364
gene-Led05385-sp3	0.654495445	5.60E-06	1.90E-05
gene-Led08940-sp3	0.579825211	0.003763602	0.008177644
gene-Led10896-sp3	0.493391204	0.007455681	0.015278981
gene-Led03470-sp3	1.438177396	0.000114908	0.000325836
gene-Led04694-sp3	1.270611549	0.006251478	0.013047803
gene-Led01537-sp3	3.560881294	1.79E-09	9.06E-09
gene-Led04187-sp3	1.412728426	1.21E-05	3.92E-05
gene-Led07149-sp3	3.701225926	3.03E-40	9.38E-39
gene-Led10372-sp3	0.717467972	6.58E-07	2.52E-06
gene-Led05563-sp3	1.299843221	1.18E-07	4.95E-07
gene-Led06645-sp3	0.794689995	0.002028934	0.004651703
gene-Led06271-sp3	3.725275586	2.51E-07	1.01E-06
gene-Led10731-sp3	0.835380302	7.69E-08	3.30E-07
gene-Led03417-sp3	1.278672313	6.56E-06	2.21E-05

gene-Led07709-sp3	0.339832754	0.023209784	0.042573522
gene-Led10809-sp3	1.091677217	0.00485051	0.010292496
gene-Led10564-sp3	0.837200835	0.009929233	0.01983788
gene-Led09364-sp3	2.041616825	1.83E-07	7.55E-07
gene-Led10528-sp3	-0.832564398	2.45E-07	9.90E-07
gene-Led08890-sp3	0.545707639	0.001242885	0.002958102
gene-Led08273-sp3	0.846379847	0.000103809	0.000296791
gene-Led05000-sp3	4.245495358	4.41E-07	1.72E-06
gene-Led02187-sp3	0.718316871	0.001700972	0.003952952
gene-Led01823-sp3	1.667763144	8.80E-06	2.91E-05
gene-Led07102-sp3	-1.367141658	0.000900878	0.002203021
gene-Led05142-sp3	2.762190766	1.34E-19	1.55E-18
gene-Led00717-sp3	2.10477794	7.57E-08	3.25E-07
gene-Led07008-sp3	-1.137306406	1.85E-15	1.58E-14
gene-Led08709-sp3	0.852896879	2.96E-06	1.04E-05
gene-Led01939-sp3	0.720749751	0.001221375	0.00291173
gene-Led01529-sp3	0.757913578	0.010645191	0.021105723
gene-Led01535-sp3	2.119701334	0.000174132	0.000482219
gene-Led04696-sp3	3.085245758	9.22E-08	3.92E-07
gene-Led09717-sp3	0.798756591	0.00154804	0.003621816
gene-Led00768-sp3	0.685902335	0.003712826	0.008079534
gene-Led10657-sp3	0.886626658	4.30E-08	1.90E-07
gene-Led02899-sp3	1.043425141	4.65E-05	0.00013847
gene-Led11265-sp3	1.050329349	2.72E-07	1.09E-06
gene-Led05276-sp3	-1.022410897	1.33E-06	4.91E-06
gene-Led02366-sp3	1.189419316	0.00021624	0.000590215
gene-Led01808-sp3	2.882142176	2.93E-13	2.06E-12
gene-Led04995-sp3	0.580018771	6.47E-06	2.18E-05
gene-Led09460-sp3	0.524956402	0.009070754	0.018268651
gene-Led06539-sp3	1.872032203	0.002550375	0.005741198
gene-Led04290-sp3	1.877856775	4.94E-15	4.05E-14
gene-Led07300-sp3	-3.094875813	5.93E-17	5.65E-16
gene-Led02138-sp3	-0.479884769	0.027185194	0.04896794
gene-Led05523-sp3	0.625687619	0.004488792	0.00958562
gene-Led01515-sp3	0.87817121	7.74E-07	2.93E-06
gene-Led05106-sp3	0.973958138	0.000400643	0.001043144
gene-Led05408-sp3	1.133819016	0.008455134	0.017166278
gene-Led05236-sp3	1.90543583	0.000154493	0.000431037
gene-Led03635-sp3	-1.197686516	0.016691686	0.031766315
gene-Led04983-sp3	0.43975726	0.012935355	0.025228582
gene-Led02795-sp3	1.866811529	5.27E-12	3.36E-11
gene-Led02802-sp3	-1.256172274	1.30E-06	4.79E-06

gene-Led09797-sp3	-0.801370887	0.009098832	0.018317865
gene-Led06729-sp3	1.140262759	1.38E-09	7.06E-09
gene-Led02405-sp3	0.430629312	0.009283743	0.018660249
gene-Led02176-sp3	1.341437874	0.000321151	0.000851605
gene-Led06063-sp3	0.821035457	4.57E-06	1.57E-05
gene-Led01681-sp3	0.691493315	0.018002592	0.034022936
gene-Led01165-sp3	0.462568129	0.022381492	0.041202838
gene-Led07346-sp3	0.9137825	0.018876032	0.03544057
gene-Led01901-sp3	0.494770286	0.023445955	0.042962181
gene-Led06779-sp3	0.502317648	0.015492761	0.029669875
gene-Led08903-sp3	0.569099607	7.72E-05	0.000224031
gene-Led10589-sp3	1.754946912	0.001576748	0.003684697
gene-Led02926-sp3	0.860401187	6.38E-09	3.06E-08
gene-Led00264-sp3	0.590047516	0.003971857	0.008578256

Table S6 Yellow module DEGS list filtered by Upset Venn diagram

gene	LeC7d_vs_LeC60d_log2FoldChange	LeC7d_vs_LeC60d_pvalue	LeC7d_vs_LeC60d_padj
gene-Led01180-sp3	3.602319152	3.59E-44	6.48E-43
gene-Led10638-sp3	2.851330455	2.04E-21	1.32E-20
gene-Led05170-sp3	2.284945038	1.94E-12	7.40E-12
gene-Led05888-sp3	4.298459828	2.54E-33	2.89E-32
gene-Led08154-sp3	3.936522594	3.32E-48	6.89E-47
gene-Led05808-sp3	2.509157077	6.91E-69	3.12E-67
gene-Led01311-sp3	0.685682613	5.67E-05	0.000114216
gene-Led00420-sp3	1.577540623	5.30E-14	2.25E-13
gene-Led09189-sp3	1.695783507	2.28E-37	3.07E-36
gene-Led02123-sp3	2.948774977	4.04E-57	1.14E-55
gene-Led00135-sp3	2.140588441	2.52E-23	1.80E-22
gene-Led10099-sp3	3.016390347	3.14E-57	8.88E-56
gene-Led02464-sp3	2.849381648	1.37E-42	2.30E-41
gene-Led00071-sp3	2.994977843	4.04E-11	1.41E-10
gene-Led10272-sp3	1.672609376	2.41E-13	9.82E-13
gene-Led02076-sp3	3.647785737	2.88E-84	2.04E-82
gene-Led06019-sp3	4.935221285	1.23E-38	1.76E-37
gene-Led02499-sp3	3.969637998	4.95E-35	6.10E-34
gene-Led06002-sp3	3.030876682	2.97E-26	2.45E-25
gene-Led08318-sp3	3.402349808	1.13E-39	1.70E-38
gene-Led09541-sp3	3.541115658	3.80E-21	2.42E-20

gene-Led02369-sp3	1.394175807	1.01E-23	7.40E-23
gene-Led05207-sp3	6.836567375	1.83E-70	8.85E-69
gene-Led11273-sp3	3.017552829	2.57E-25	2.03E-24
gene-Led10789-sp3	4.09344091	3.90E-43	6.71E-42
gene-Led04561-sp3	0.640300505	0.00041466	0.000763905
gene-Led04360-sp3	4.015485309	3.47E-40	5.32E-39
gene-Led06838-sp3	3.64730687	3.56E-44	6.44E-43
gene-Led04635-sp3	6.820709853	2.62E-204	1.38E-201
gene-Led05102-sp3	8.154768394	0	0
gene-Led00307-sp3	1.798310133	2.66E-24	2.00E-23
gene-Led10319-sp3	2.89479986	8.14E-44	1.44E-42
gene-Led04126-sp3	3.539755718	3.61E-32	3.89E-31
gene-Led10800-sp3	6.369450331	3.96E-210	2.34E-207
gene-Led10556-sp3	3.199756485	6.12E-29	5.67E-28
gene-Led05062-sp3	7.749417368	1.14E-65	4.48E-64
gene-Led01747-sp3	5.519452205	5.17E-97	4.82E-95
gene-Led03248-sp3	3.100254281	1.06E-56	2.91E-55
gene-Led01318-sp3	2.707101946	5.25E-55	1.36E-53
gene-Led04816-sp3	0.798270351	0.001004437	0.001770267
gene-Led02401-sp3	1.958535329	2.82E-32	3.06E-31
gene-Led07402-sp3	1.084413129	1.95E-05	4.11E-05
gene-Led05140-sp3	3.176387027	1.89E-18	1.03E-17
gene-Led07463-sp3	1.792265401	1.41E-17	7.29E-17
gene-Led04066-sp3	4.691009326	6.86E-96	6.27E-94
gene-Led04911-sp3	5.750911578	2.03E-57	5.79E-56
gene-Led04564-sp3	2.685173262	1.96E-25	1.55E-24
gene-Led08181-sp3	3.091722796	4.67E-65	1.80E-63
gene-Led04123-sp3	3.47872035	1.54E-64	5.81E-63
gene-Led08548-sp3	0.33490284	0.016569513	0.024699484
gene-Led01310-sp3	1.535438975	1.64E-25	1.31E-24
gene-Led06769-sp3	1.352778076	1.14E-12	4.43E-12
gene-Led01848-sp3	0.857510003	0.004090849	0.006657812
gene-Led07212-sp3	4.420615267	4.48E-145	9.38E-143
gene-Led00334-sp3	1.642590856	1.46E-18	8.02E-18
gene-Led08246-sp3	0.677426257	0.00011385	0.000222153
gene-Led08563-sp3	1.971723796	1.25E-11	4.50E-11
gene-Led10585-sp3	1.003406291	0.000888924	0.001575799
gene-Led03861-sp3	-1.202789088	1.91E-17	9.83E-17
gene-Led05060-sp3	1.94556818	4.11E-26	3.37E-25
gene-Led01148-sp3	3.024247729	1.01E-78	6.20E-77
gene-Led03190-sp3	1.786210464	8.35E-15	3.71E-14
gene-Led08443-sp3	1.205009205	1.14E-19	6.69E-19

gene-Led07117-sp3	7.672328849	5.62E-78	3.36E-76
gene-Led08569-sp3	6.002980904	1.91E-245	2.40E-242
gene-Led06951-sp3	6.628204334	4.21E-58	1.23E-56
gene-Led02408-sp3	1.452062104	7.71E-22	5.12E-21
gene-Led09812-sp3	1.745759183	2.94E-14	1.27E-13
gene-Led01964-sp3	3.202987204	9.50E-07	2.27E-06
gene-Led11092-sp3	1.751364344	4.78E-12	1.77E-11
gene-Led07397-sp3	4.459374924	2.99E-39	4.40E-38
gene-Led00037-sp3	2.998989138	2.09E-29	1.98E-28
gene-Led10799-sp3	2.168819497	1.37E-45	2.61E-44
gene-Led08977-sp3	1.643501095	7.03E-11	2.41E-10
gene-Led06159-sp3	0.380685761	0.031162873	0.044355141
gene-Led01888-sp3	4.12068743	3.51E-79	2.19E-77
gene-Led02390-sp3	2.193292984	1.01E-09	3.17E-09
gene-Led10054-sp3	6.297520609	2.59E-48	5.43E-47
gene-Led01433-sp3	2.191473773	3.79E-22	2.55E-21
gene-Led02036-sp3	3.006712077	1.20E-24	9.17E-24
gene-Led00019-sp3	3.649873527	4.10E-29	3.84E-28
gene-Led05604-sp3	3.391269258	9.14E-36	1.15E-34
gene-Led04836-sp3	1.069677262	9.14E-08	2.41E-07
gene-Led05752-sp3	2.361918384	4.37E-39	6.37E-38
gene-Led03421-sp3	3.675881748	5.12E-42	8.34E-41
gene-Led10746-sp3	0.297519732	0.034724475	0.048973786
gene-Led08250-sp3	2.677141115	4.01E-49	8.70E-48
gene-Led01870-sp3	2.283649353	1.72E-57	4.91E-56
gene-Led07083-sp3	1.515487365	1.19E-19	6.98E-19
gene-Led11391-sp3	3.291369595	4.06E-62	1.37E-60
gene-Led10244-sp3	2.365289872	4.88E-39	7.07E-38
gene-Led05235-sp3	0.761780562	1.12E-06	2.66E-06
gene-Led01674-sp3	3.366863868	9.05E-76	5.14E-74
gene-Led03701-sp3	3.601634359	5.03E-28	4.51E-27
gene-Led03854-sp3	2.219585099	4.60E-12	1.71E-11
gene-Led06120-sp3	4.75547349	2.59E-85	1.93E-83
gene-Led10807-sp3	1.213562687	0.001793758	0.00306313
gene-Led09753-sp3	5.466375584	2.24E-119	3.41E-117
gene-Led07874-sp3	2.759894223	4.84E-17	2.44E-16
gene-Led05328-sp3	2.370862664	6.13E-26	4.99E-25
gene-Led06492-sp3	2.488232091	8.94E-37	1.18E-35
gene-Led07988-sp3	2.106868789	3.82E-28	3.44E-27
gene-Led04749-sp3	2.927085247	1.78E-44	3.27E-43
gene-Led03170-sp3	3.221082628	1.64E-29	1.57E-28
gene-Led01384-sp3	2.2884727	9.35E-17	4.63E-16

gene-Led03718-sp3	2.425264114	5.75E-27	4.91E-26
gene-Led01300-sp3	3.919298363	1.16E-50	2.63E-49
gene-Led02914-sp3	1.971617972	1.19E-16	5.85E-16
gene-Led05052-sp3	2.247858638	3.38E-11	1.18E-10
gene-Led01965-sp3	2.686749266	2.12E-40	3.27E-39
gene-Led05541-sp3	1.052536478	6.13E-10	1.96E-09
gene-Led10637-sp3	2.457565589	9.08E-32	9.65E-31
gene-Led01050-sp3	0.446226012	0.000373702	0.000691355
gene-Led09407-sp3	0.883193697	6.48E-06	1.44E-05
gene-Led00581-sp3	0.903097756	6.66E-09	1.95E-08
gene-Led10998-sp3	-1.033290175	5.40E-06	1.21E-05
gene-Led00655-sp3	-1.03935504	1.58E-07	4.06E-07
gene-Led07527-sp3	2.954799708	4.77E-62	1.60E-60
gene-Led10059-sp3	0.380748744	0.025831046	0.037219365
gene-Led07583-sp3	1.665561096	5.22E-20	3.12E-19
gene-Led02526-sp3	4.880868147	8.20E-86	6.15E-84
gene-Led03264-sp3	4.857196902	7.56E-38	1.04E-36
gene-Led07315-sp3	2.650379235	2.85E-24	2.13E-23
gene-Led05204-sp3	2.758258998	6.53E-10	2.08E-09
gene-Led07608-sp3	1.821708617	1.72E-36	2.26E-35
gene-Led03654-sp3	4.426263111	6.01E-50	1.35E-48
gene-Led01117-sp3	-2.419943653	1.23E-19	7.19E-19
gene-Led06932-sp3	1.682348593	4.15E-41	6.56E-40
gene-Led09887-sp3	5.272869511	1.47E-52	3.51E-51
gene-Led00177-sp3	2.124965422	4.00E-09	1.19E-08
gene-Led09653-sp3	2.666121071	4.50E-46	8.71E-45
gene-Led04938-sp3	6.392409857	2.27E-102	2.33E-100
gene-Led04692-sp3	3.533326482	7.71E-30	7.51E-29
gene-Led07357-sp3	4.568654037	8.04E-37	1.06E-35
gene-Led06145-sp3	-2.895768299	3.51E-17	1.78E-16
gene-Led02240-sp3	-2.111215086	7.81E-42	1.27E-40
gene-Led01182-sp3	2.067086612	2.04E-29	1.94E-28
gene-Led07993-sp3	1.211399206	1.52E-15	7.00E-15
gene-Led08984-sp3	1.6799919	5.68E-14	2.40E-13
gene-Led10158-sp3	1.052719206	2.86E-10	9.35E-10
gene-Led09959-sp3	4.826021274	2.85E-38	4.00E-37
gene-Led01440-sp3	1.887902684	2.20E-07	5.58E-07
gene-Led06162-sp3	4.051097061	6.45E-33	7.22E-32
gene-Led08611-sp3	3.073385075	2.61E-109	3.08E-107
gene-Led06491-sp3	2.678896515	4.74E-17	2.39E-16
gene-Led09124-sp3	1.647443996	1.47E-20	9.04E-20
gene-Led07923-sp3	3.066508681	2.41E-53	5.91E-52

gene-Led02628-sp3	1.509462385	2.41E-08	6.71E-08
gene-Led01004-sp3	2.563196679	2.89E-16	1.40E-15
gene-Led10625-sp3	1.52150231	1.26E-12	4.86E-12
gene-Led02386-sp3	1.642014196	2.79E-26	2.31E-25
gene-Led07171-sp3	4.865122112	1.26E-239	1.41E-236
gene-Led08404-sp3	2.561882784	1.55E-30	1.57E-29
gene-Led06793-sp3	3.986728061	1.03E-24	7.87E-24
gene-Led10130-sp3	2.124755073	4.90E-15	2.20E-14
gene-Led09401-sp3	2.386517311	9.57E-14	3.98E-13
gene-Led10302-sp3	1.02954737	3.53E-13	1.42E-12
gene-Led06129-sp3	1.818068537	2.34E-13	9.52E-13
gene-Led02368-sp3	3.613307856	3.14E-54	7.94E-53
gene-Led03791-sp3	2.661461168	4.16E-49	9.02E-48
gene-Led10458-sp3	3.168129031	1.64E-12	6.30E-12
gene-Led02110-sp3	2.931771401	1.37E-32	1.52E-31
gene-Led10160-sp3	1.16506317	3.88E-06	8.81E-06
gene-Led02054-sp3	5.282255221	2.06E-16	1.00E-15
gene-Led05311-sp3	1.743469402	5.24E-29	4.88E-28
gene-Led07906-sp3	-0.697656895	3.35E-08	9.18E-08
gene-Led09666-sp3	-1.410544979	2.92E-10	9.56E-10
gene-Led04292-sp3	-0.934402672	5.09E-10	1.64E-09
gene-Led06024-sp3	4.132398954	1.53E-42	2.55E-41
gene-Led01122-sp3	2.192079126	1.90E-75	1.07E-73
gene-Led02124-sp3	1.229114257	1.02E-09	3.21E-09
gene-Led06015-sp3	2.274842652	1.38E-30	1.40E-29
gene-Led08476-sp3	1.449157764	2.11E-10	6.96E-10
gene-Led09647-sp3	3.068929913	1.08E-34	1.31E-33
gene-Led05929-sp3	1.051926532	1.80E-20	1.10E-19
gene-Led01835-sp3	1.239655653	8.26E-15	3.67E-14
gene-Led07277-sp3	2.521428062	1.48E-18	8.13E-18
gene-Led08310-sp3	2.854776316	1.80E-17	9.30E-17
gene-Led03385-sp3	3.619161593	3.31E-21	2.12E-20
gene-Led10175-sp3	5.228098359	1.46E-77	8.67E-76
gene-Led01195-sp3	2.194691082	1.86E-38	2.63E-37
gene-Led01936-sp3	4.808841824	1.03E-109	1.24E-107
gene-Led05996-sp3	4.530042835	7.95E-54	1.98E-52
gene-Led01286-sp3	3.01183629	1.06E-103	1.12E-101
gene-Led04784-sp3	4.974089807	1.72E-23	1.24E-22
gene-Led04501-sp3	1.550854752	2.36E-26	1.96E-25
gene-Led07298-sp3	6.177495682	3.54E-93	3.07E-91
gene-Led03051-sp3	2.385482688	5.43E-39	7.86E-38
gene-Led10353-sp3	2.190677902	1.45E-65	5.65E-64

gene-Led04192-sp3	1.780575138	5.42E-09	1.60E-08
gene-Led03617-sp3	1.793013873	8.30E-05	0.000164035
gene-Led05150-sp3	2.384231833	2.49E-11	8.79E-11
gene-Led10214-sp3	2.55783351	1.56E-16	7.64E-16
gene-Led01851-sp3	3.542024221	6.34E-58	1.84E-56
gene-Led04410-sp3	-0.807855252	1.69E-08	4.77E-08
gene-Led02364-sp3	2.729021892	1.00E-33	1.16E-32
gene-Led00385-sp3	1.53919118	6.77E-15	3.03E-14
gene-Led04988-sp3	-0.538491493	0.00510914	0.008214008
gene-Led01575-sp3	1.606242199	7.42E-13	2.91E-12
gene-Led01633-sp3	2.02634907	1.12E-17	5.82E-17
gene-Led08101-sp3	2.498780923	1.79E-15	8.24E-15
gene-Led04470-sp3	2.226981725	4.74E-14	2.02E-13
gene-Led09888-sp3	1.206102688	0.004194681	0.006817968
gene-Led00829-sp3	0.576349045	0.003159454	0.005228312
gene-Led01878-sp3	0.414363889	0.020936931	0.030664185
gene-Led05281-sp3	1.859140181	5.01E-09	1.48E-08
gene-Led10825-sp3	1.602986344	8.21E-05	0.000162253
gene-Led08270-sp3	2.499846116	3.46E-21	2.22E-20
gene-Led02186-sp3	0.634034742	0.003943147	0.006436183
gene-Led07304-sp3	1.765654154	3.64E-29	3.42E-28
gene-Led10917-sp3	3.031822326	1.89E-28	1.72E-27
gene-Led10032-sp3	1.857424366	3.34E-09	1.00E-08
gene-Led00378-sp3	0.990510276	0.014245947	0.021471439
gene-Led11368-sp3	1.582868808	2.20E-09	6.67E-09
gene-Led01199-sp3	-0.72863972	0.000677212	0.001219856
gene-Led01475-sp3	1.536638373	3.47E-15	1.58E-14
gene-Led09648-sp3	0.915609636	8.81E-06	1.92E-05
gene-Led10811-sp3	2.596505599	5.73E-44	1.02E-42
gene-Led00087-sp3	-0.502788239	0.005872989	0.009373101
gene-Led00930-sp3	-1.042714035	1.73E-14	7.57E-14
gene-Led09302-sp3	1.542792109	5.92E-07	1.44E-06
gene-Led03000-sp3	-0.860631088	8.51E-06	1.86E-05
gene-Led06719-sp3	-0.909249264	1.37E-08	3.92E-08
gene-Led01774-sp3	-0.657762728	1.41E-06	3.30E-06
gene-Led07294-sp3	1.375211252	1.26E-05	2.70E-05
gene-Led06058-sp3	2.53436606	8.90E-21	5.56E-20
gene-Led08433-sp3	-0.720113338	8.34E-05	0.000164803
gene-Led06030-sp3	2.399745782	2.89E-11	1.02E-10
gene-Led08499-sp3	-0.934276382	1.42E-08	4.06E-08
gene-Led09343-sp3	-2.247816231	4.09E-30	4.04E-29
gene-Led07793-sp3	-1.432565236	3.37E-17	1.72E-16

gene-Led01885-sp3	1.552629406	1.89E-18	1.03E-17
gene-Led09548-sp3	1.888768057	6.13E-07	1.49E-06
gene-Led02233-sp3	-0.679015482	2.22E-05	4.66E-05
gene-Led06739-sp3	2.223038388	5.92E-33	6.64E-32
gene-Led01447-sp3	1.981333974	3.61E-22	2.43E-21
gene-Led07211-sp3	1.287538417	1.01E-12	3.92E-12
gene-Led08319-sp3	4.870501678	8.01E-64	2.93E-62
gene-Led05836-sp3	3.102847421	1.42E-05	3.03E-05
gene-Led06003-sp3	3.872046826	7.29E-34	8.52E-33
gene-Led07367-sp3	1.64484273	3.07E-09	9.21E-09
gene-Led01819-sp3	5.86159612	5.35E-19	3.02E-18
gene-Led08079-sp3	0.93208633	3.25E-08	8.92E-08
gene-Led00700-sp3	0.921933382	1.39E-07	3.61E-07
gene-Led06168-sp3	1.112103195	1.31E-07	3.39E-07
gene-Led04233-sp3	2.023579897	8.90E-27	7.56E-26
gene-Led02004-sp3	1.19111406	2.35E-14	1.02E-13
gene-Led10666-sp3	1.116099261	6.76E-07	1.63E-06
gene-Led10105-sp3	-0.535824288	0.000753723	0.001347773
gene-Led10676-sp3	2.660830438	5.87E-14	2.48E-13
gene-Led05648-sp3	-1.100616717	1.03E-11	3.73E-11
gene-Led10355-sp3	1.096522045	2.39E-10	7.87E-10
gene-Led08265-sp3	1.547094395	1.18E-11	4.27E-11
gene-Led06825-sp3	1.321821957	1.51E-17	7.83E-17
gene-Led01954-sp3	1.319425191	6.92E-10	2.20E-09
gene-Led10009-sp3	-0.345567947	0.018377888	0.027181538
gene-Led00048-sp3	0.662082682	2.76E-05	5.73E-05
gene-Led05781-sp3	1.089584574	2.07E-08	5.79E-08
gene-Led01588-sp3	1.066148299	1.09E-12	4.23E-12
gene-Led01088-sp3	1.348592362	3.23E-06	7.36E-06
gene-Led01317-sp3	2.324764196	6.33E-19	3.55E-18
gene-Led00767-sp3	-1.398449057	5.40E-18	2.86E-17
gene-Led00939-sp3	0.801124846	3.38E-08	9.26E-08
gene-Led03934-sp3	1.709656995	9.94E-14	4.13E-13
gene-Led08201-sp3	-0.851752146	1.14E-08	3.27E-08
gene-Led09892-sp3	0.985122428	6.18E-08	1.65E-07
gene-Led05887-sp3	3.319953357	1.94E-38	2.75E-37
gene-Led04927-sp3	4.087718229	3.64E-27	3.15E-26
gene-Led02197-sp3	0.32243314	0.017618065	0.026134633
gene-Led05028-sp3	0.81115679	0.004744353	0.007658153
gene-Led04475-sp3	5.348287896	2.59E-12	9.80E-12
gene-Led05357-sp3	-0.597249933	0.025102123	0.036236594
gene-Led10240-sp3	1.451649254	1.53E-08	4.36E-08

gene-Led06521-sp3	-0.632089936	4.62E-05	9.39E-05
gene-Led08078-sp3	1.483063061	2.58E-13	1.05E-12
gene-Led00534-sp3	1.707388684	5.06E-12	1.87E-11
gene-Led08312-sp3	0.760347439	0.004775408	0.007705806
gene-Led07101-sp3	-0.423756212	0.011544724	0.017662321
gene-Led04835-sp3	2.322593505	6.40E-22	4.27E-21
gene-Led04633-sp3	-0.648219717	0.000421295	0.00077555
gene-Led03194-sp3	1.016407873	1.87E-07	4.79E-07
gene-Led10348-sp3	0.930643784	1.39E-08	3.97E-08
gene-Led05143-sp3	-0.463808563	0.002043327	0.003464008
gene-Led09620-sp3	1.663100823	0.013989842	0.021111588
gene-Led06037-sp3	0.730653307	5.73E-05	0.000115368
gene-Led04339-sp3	-0.645023762	0.000784167	0.001399722
gene-Led02851-sp3	-0.825095764	1.07E-08	3.08E-08
gene-Led06061-sp3	0.740778771	2.42E-05	5.05E-05
gene-Led04041-sp3	-0.388111213	0.01340684	0.020313333
gene-Led10354-sp3	0.42539637	0.016297158	0.024331229
gene-Led02889-sp3	0.74707634	7.15E-05	0.000142458
gene-Led10756-sp3	-0.309980823	0.017136635	0.02547952
gene-Led02772-sp3	0.454073363	0.000737922	0.001321635
gene-Led11004-sp3	0.617532611	0.01240746	0.018913146
gene-Led00537-sp3	1.168918056	7.53E-08	2.00E-07
gene-Led05225-sp3	0.480099311	0.004310683	0.006997464
gene-Led02557-sp3	-0.430674621	0.008397177	0.013096001
gene-Led05621-sp3	0.391694446	0.011861967	0.018121141
gene-Led02394-sp3	0.340879056	0.020492248	0.030064157
gene-Led09429-sp3	1.051655037	3.51E-07	8.73E-07
gene-Led05064-sp3	1.715143944	2.01E-06	4.67E-06
gene-Led06409-sp3	1.690106863	2.55E-26	2.12E-25
gene-Led01798-sp3	-0.733812657	0.000237443	0.000448688
gene-Led03596-sp3	0.956912804	3.99E-09	1.19E-08
gene-Led06040-sp3	-0.405968439	0.015997724	0.02391096
gene-Led03630-sp3	-0.334669655	0.025458167	0.036718931
gene-Led05660-sp3	-1.043288487	5.41E-11	1.87E-10
gene-Led00513-sp3	-0.896721124	2.68E-09	8.09E-09
gene-Led08658-sp3	-3.730892894	3.45E-09	1.03E-08
gene-Led02631-sp3	-0.815553247	0.002936854	0.00489054
gene-Led00159-sp3	-0.586419348	5.77E-06	1.29E-05
gene-Led09335-sp3	0.659475473	1.87E-06	4.35E-06
gene-Led03071-sp3	-0.658227375	0.0047051	0.007602116
gene-Led09954-sp3	-0.619447608	2.60E-06	5.97E-06
gene-Led05013-sp3	-0.375991762	0.003477982	0.005718703

gene-Led07201-sp3	-1.117994811	2.94E-06	6.71E-06
gene-Led05663-sp3	-0.876630004	9.04E-09	2.62E-08
gene-Led01944-sp3	0.666917771	0.017112643	0.02546207
gene-Led08644-sp3	-0.634331165	0.001881763	0.003203618
gene-Led04116-sp3	0.573398516	0.001069001	0.001877476
gene-Led10774-sp3	-0.726091077	0.000385985	0.00071316
gene-Led09293-sp3	-0.944000633	0.00482381	0.007780165
gene-Led02515-sp3	-0.589471529	0.007543674	0.011867503
gene-Led08891-sp3	0.994079432	8.06E-07	1.94E-06
gene-Led05524-sp3	-0.661165476	0.000450916	0.000827503
gene-Led02482-sp3	0.612169152	0.00725957	0.011449711
gene-Led00881-sp3	-0.329228695	0.033854256	0.04784448
gene-Led07855-sp3	-0.665801767	2.87E-07	7.20E-07
gene-Led08876-sp3	0.337922121	0.007613607	0.011963049
gene-Led08978-sp3	0.76090869	3.87E-05	7.93E-05
gene-Led08920-sp3	0.542336897	0.002215608	0.00373842
gene-Led02569-sp3	-1.25317367	1.10E-22	7.60E-22
gene-Led00588-sp3	0.559326589	0.005296151	0.008499714
gene-Led06737-sp3	-0.365076336	0.024531811	0.035464235
gene-Led02963-sp3	-0.622209923	0.002310913	0.003891391
gene-Led10645-sp3	0.573760154	0.003792852	0.006202952
gene-Led10188-sp3	0.869839845	4.35E-07	1.07E-06
gene-Led06663-sp3	-1.015549768	5.87E-05	0.000117962
gene-Led03372-sp3	-0.562896618	7.49E-06	1.65E-05
gene-Led04279-sp3	0.703654522	0.003130853	0.005186958
gene-Led06597-sp3	-0.377227001	0.016780393	0.024987896
gene-Led02388-sp3	0.597843565	9.05E-05	0.000178102
gene-Led08729-sp3	0.806208445	2.31E-07	5.84E-07
gene-Led09551-sp3	2.622041483	1.75E-07	4.49E-07
gene-Led02085-sp3	-0.526171951	0.001588279	0.002735007
gene-Led02762-sp3	-1.577894112	0.001752814	0.002999837
gene-Led01777-sp3	0.903866061	5.02E-06	1.13E-05
gene-Led04552-sp3	-0.662199967	0.00012481	0.000242317
gene-Led01541-sp3	-0.654027723	0.029394302	0.041980526
gene-Led09314-sp3	0.49854539	0.004171354	0.006783342
gene-Led10597-sp3	0.83909103	0.000828322	0.001474087
gene-Led08488-sp3	-0.535131745	0.003257175	0.005375863
gene-Led00891-sp3	-0.493182581	0.007502733	0.011812838
gene-Led07454-sp3	-0.352805157	0.006171609	0.009813854
gene-Led03763-sp3	-0.646511227	0.000873159	0.001550312
gene-Led04771-sp3	0.961627345	0.004068636	0.006627025
gene-Led04184-sp3	-0.508640081	0.023606723	0.034225322

gene-Led00790-sp3	0.800634875	8.93E-09	2.59E-08
gene-Led05214-sp3	-0.448377242	0.003052549	0.005066415
gene-Led00864-sp3	0.616953232	0.028640621	0.040997318
gene-Led11123-sp3	1.384889447	1.08E-16	5.35E-16
gene-Led06515-sp3	-0.270861399	0.018026008	0.02668465
gene-Led05960-sp3	-1.111878337	0.000264757	0.000497217
gene-Led11098-sp3	-1.80215842	5.74E-19	3.23E-18
gene-Led05088-sp3	-0.65450354	0.001614581	0.002776968
gene-Led01658-sp3	0.588009445	0.000279206	0.00052357
gene-Led02088-sp3	-0.64932746	3.89E-06	8.82E-06
gene-Led05628-sp3	-1.002262192	4.73E-10	1.52E-09
gene-Led03499-sp3	1.060811965	1.33E-09	4.10E-09
gene-Led06594-sp3	-0.814270459	9.48E-06	2.06E-05
gene-Led05358-sp3	0.85081649	4.59E-07	1.13E-06
gene-Led07760-sp3	-0.719279654	3.31E-07	8.28E-07
gene-Led00912-sp3	0.778849891	0.000743184	0.001330347
gene-Led02986-sp3	0.4592942	0.012794322	0.019455653
gene-Led01872-sp3	1.702280944	1.70E-18	9.31E-18
gene-Led01383-sp3	0.865087408	1.33E-05	2.83E-05
gene-Led07514-sp3	0.524920322	0.001537695	0.002652901
gene-Led02809-sp3	-0.44409907	0.000383012	0.000707797
gene-Led09017-sp3	-0.392373676	0.011762222	0.017975923
gene-Led02758-sp3	1.446377277	4.76E-07	1.17E-06
gene-Led06130-sp3	1.008949438	0.002905619	0.004842538
gene-Led07440-sp3	0.483988515	0.006555524	0.010399665
gene-Led10929-sp3	-0.663788475	0.000522383	0.000952052
gene-Led03049-sp3	1.020401409	9.71E-10	3.05E-09
gene-Led02195-sp3	0.688314529	0.000412594	0.000760369
gene-Led06775-sp3	-0.318148617	0.017865541	0.026466591
gene-Led04155-sp3	-0.320200476	0.007530775	0.011851422
gene-Led05163-sp3	0.475277657	0.000777353	0.001388075
gene-Led05616-sp3	1.033176993	1.88E-06	4.37E-06
gene-Led02359-sp3	0.662360119	1.87E-06	4.35E-06
gene-Led02127-sp3	0.74048691	0.002863623	0.004775713
gene-Led08470-sp3	-0.368438064	0.018909695	0.027910609
gene-Led03746-sp3	-0.582184922	0.000357843	0.000664094
gene-Led02136-sp3	0.601724819	0.003642956	0.005974329
gene-Led10226-sp3	0.80224479	1.06E-07	2.79E-07
gene-Led07491-sp3	-1.209976063	7.62E-10	2.41E-09
gene-Led09032-sp3	0.384585107	0.000974451	0.00171983
gene-Led02290-sp3	-1.559274976	0.000272949	0.000512218
gene-Led05585-sp3	1.185084084	3.56E-13	1.43E-12

gene-Led03124-sp3	0.725358402	7.23E-06	1.59E-05
gene-Led05256-sp3	-0.954076811	6.74E-07	1.63E-06
gene-Led03453-sp3	-1.030120239	2.15E-05	4.51E-05
gene-Led04450-sp3	-0.65775514	0.016727275	0.024912488
gene-Led06881-sp3	0.979867041	1.95E-09	5.95E-09
gene-Led02303-sp3	-0.432713142	0.000452862	0.000830771
gene-Led02987-sp3	-0.711046073	2.37E-05	4.95E-05
gene-Led10265-sp3	2.295692864	2.49E-09	7.52E-09
gene-Led03716-sp3	-0.891451733	0.028950128	0.041393207
gene-Led11162-sp3	-0.775972136	0.000792049	0.001412788
gene-Led07986-sp3	-0.34950209	0.029213396	0.041734016
gene-Led08645-sp3	0.990454286	2.25E-07	5.70E-07
gene-Led10775-sp3	-0.475656255	0.000433345	0.000796419
gene-Led01360-sp3	0.853444588	0.006815808	0.010790444
gene-Led04713-sp3	0.742344737	0.000502321	0.000917317
gene-Led09053-sp3	0.381761368	0.005489559	0.008794667
gene-Led09859-sp3	0.53129897	0.000166917	0.000319622
gene-Led08690-sp3	0.87931747	3.50E-08	9.59E-08
gene-Led08877-sp3	1.602977622	9.21E-08	2.43E-07
gene-Led11274-sp3	0.949317693	0.007656243	0.012018772
gene-Led05640-sp3	-0.815951056	1.31E-09	4.06E-09
gene-Led10288-sp3	-0.431769726	0.003752866	0.006141553
gene-Led00508-sp3	0.579998785	9.43E-05	0.000185176
gene-Led00970-sp3	-0.27900532	0.012059151	0.018410121
gene-Led07893-sp3	0.670631321	0.000231456	0.000438033
gene-Led09675-sp3	-0.401539399	0.015853879	0.023710064
gene-Led03970-sp3	-0.479526767	9.63E-05	0.00018914
gene-Led05930-sp3	0.459942496	0.0090021	0.013991668
gene-Led07594-sp3	-0.616256037	0.007699164	0.01208049
gene-Led07989-sp3	-0.444937095	0.005676932	0.009074606
gene-Led00949-sp3	0.611126779	1.94E-06	4.50E-06
gene-Led08137-sp3	-0.394024752	0.015247562	0.02286452
gene-Led10485-sp3	0.841382465	7.66E-07	1.84E-06
gene-Led02952-sp3	-0.539403802	0.000893681	0.001583674
gene-Led00536-sp3	0.722754278	7.87E-06	1.72E-05
gene-Led00568-sp3	-0.363398491	0.019647411	0.028939989
gene-Led09211-sp3	0.619062056	0.000846592	0.001505269
gene-Led00923-sp3	-0.695949899	6.05E-07	1.47E-06
gene-Led04242-sp3	-0.390634389	0.000746435	0.001335929
gene-Led06602-sp3	-0.740664502	4.46E-05	9.09E-05
gene-Led08659-sp3	-2.48824501	0.000177677	0.000339128
gene-Led08594-sp3	0.423155343	0.01766277	0.026197082

gene-Led05553-sp3	-0.314675416	0.021495333	0.031399678
gene-Led08292-sp3	0.666723185	0.004642	0.00750982
gene-Led04504-sp3	-0.394696985	0.024635837	0.0356095
gene-Led06255-sp3	-0.538682463	0.00274317	0.004586245
gene-Led07058-sp3	-0.606853652	0.003875617	0.006331098
gene-Led03600-sp3	0.673701263	9.79E-05	0.000192092
gene-Led06667-sp3	1.703306146	3.20E-17	1.63E-16
gene-Led08814-sp3	-0.590756786	3.44E-05	7.08E-05
gene-Led10120-sp3	-0.734881907	0.003198072	0.005283519
gene-Led05215-sp3	-0.695671316	0.000517445	0.000943394
gene-Led01462-sp3	1.51130489	2.73E-07	6.87E-07
gene-Led10347-sp3	1.080696299	0.00160113	0.002755719
gene-Led10782-sp3	-0.517400973	0.00065512	0.001182391
gene-Led02336-sp3	0.776418015	3.54E-07	8.79E-07
gene-Led05727-sp3	-0.94932277	9.56E-10	3.00E-09
gene-Led01708-sp3	-0.557003006	0.001517673	0.002619706
gene-Led02923-sp3	0.550971401	0.014347546	0.021601899
gene-Led10675-sp3	-0.730771305	0.000105275	0.000205959
gene-Led06114-sp3	0.654458634	1.01E-05	2.19E-05
gene-Led03561-sp3	0.976780951	0.000171047	0.00032698
gene-Led00465-sp3	0.70799844	5.51E-06	1.23E-05
gene-Led00895-sp3	0.844612856	1.75E-07	4.51E-07
gene-Led06001-sp3	0.645164573	0.000401478	0.000740834
gene-Led02955-sp3	0.578400068	0.010805624	0.016599777
gene-Led05179-sp3	-0.444148698	0.018598669	0.027465927
gene-Led07830-sp3	-0.847270828	5.93E-07	1.44E-06
gene-Led06495-sp3	-0.563903553	6.29E-06	1.40E-05
gene-Led01737-sp3	-0.417715292	0.012853342	0.019533582
gene-Led02313-sp3	-0.951364488	0.001353922	0.002351585
gene-Led10306-sp3	1.59627981	1.84E-12	7.03E-12
gene-Led06520-sp3	0.769512492	0.000115735	0.000225525
gene-Led02392-sp3	0.478358348	0.002479567	0.004160061
gene-Led07616-sp3	-0.428822895	0.000595141	0.001078204
gene-Led09934-sp3	-0.936656125	5.62E-06	1.25E-05
gene-Led00916-sp3	-0.296763727	0.022254119	0.032418585
gene-Led05500-sp3	-0.756235163	3.26E-05	6.72E-05
gene-Led07720-sp3	0.544807386	0.000459649	0.000842761
gene-Led00303-sp3	0.514971331	0.020346712	0.029882468
gene-Led10293-sp3	0.892899341	0.001161624	0.002031983
gene-Led05642-sp3	-0.513550947	0.001288462	0.002240601
gene-Led06092-sp3	0.62423447	0.00176802	0.003023286
gene-Led00975-sp3	-1.099459573	0.00050849	0.000928246

gene-Led02069-sp3	1.779147584	1.42E-09	4.39E-09
gene-Led03128-sp3	0.734708296	1.31E-08	3.77E-08
gene-Led02271-sp3	-1.053628709	5.16E-16	2.46E-15
gene-Led10264-sp3	0.702378501	0.01179845	0.018028549
gene-Led02182-sp3	-0.548630617	0.000545783	0.000992359
gene-Led05680-sp3	-0.62597148	7.97E-05	0.000157701
gene-Led09267-sp3	-0.673345974	0.000203449	0.000386484
gene-Led10083-sp3	-0.479539848	0.025720646	0.037081551
gene-Led04812-sp3	0.644008294	0.00103736	0.001823816
gene-Led00892-sp3	0.688129559	0.000910264	0.001610788
gene-Led00779-sp3	0.356255825	0.017007461	0.025318527
gene-Led02697-sp3	-0.398238911	0.007124336	0.011254078

Table S7 The top 40 enriched KEGG terms of DEGs in blue and yellow modle								
**KEGG_A_ class**	**Pathway**	**out (228)**	**All (2063)**	**Pvalue**	**Qvalue**	**Pathway ID**	**Genes**	**K_IDs**
Metabolism	Phenylalanine, tyrosine and tryptophan biosynthesis	11	21	3.08E-06	0.000317	ko00400	bluegene-Led00242-sp3;bluegene-Led02135-sp3;bluegene-Led07116-sp3;bluegene-Led00717-sp3;bluegene-Led05523-sp3;yellowgene-Led05170-sp3;yellowgene-Led11273-sp3;yellowgene-Led02036-sp3;yellowgene-Led01633-sp3;yellowgene-Led10676-sp3;yellowgene-Led05640-sp3	K01694;K01736;K00817;K01657;K00211;K13501;K00766;K00766;K13830;K01626;K13501

Metabolism	Metabolic pathways	126	933	0.0008138	0.0419097	ko01100	bluegene-Led04826-sp3;bluegene-Led07043-sp3;bluegene-Led07148-sp3;bluegene-Led03563-sp3;bluegene-Led09414-sp3;bluegene-Led08115-sp3;bluegene-Led02291-sp3;bluegene-Led04609-sp3;bluegene-Led00416-sp3;bluegene-Led01865-sp3;bluegene-Led01020-sp3;bluegene-Led05720-sp3;bluegene-Led05184-sp3;bluegene-Led07502-sp3;bluegene-Led09363-sp3;bluegene-Led01439-sp3;bluegene-Led10636-sp3;bluegene-Led09381-sp3;bluegene-Led08479-sp3;bluegene-Led00283-sp3;bluegene-Led06506-sp3;bluegene-Led05131-sp3;bluegene-Led02284-sp3;bluegene-Led06378-sp3;bluegene-Led00805-sp3;bluegene-Led00242-sp3;bluegene-Led06651-sp3;bluegene-Led06571-sp3;bluegene-Led05522-sp3;bluegene-Led02641-sp3;bluegene-Led10332-sp3;bluegene-Led02321-sp3;bluegene-Led11358-sp3;bluegene-Led05186-sp3;bluegene-Led09307-sp3;bluegene-Led06223-sp3;bluegene-Led00741-sp3;bluegene-Led00871-sp3;bluegene-Led05872-sp3;bluegene-Led03564-sp3;bluegene-Led04605-sp3;bluegene-Led03479-sp3;bluegene-Led09684-sp3;bluegene-Led03549-sp3;bluegene-Led03998-sp3;bluegene-Led07015-sp3;bluegene-Led03809-sp3;bluegene-Led06807-sp3;bluegene-Led07154-sp3;bluegene-Led02612-sp3;bluegene-Led09673-sp3;bluegene-Led02084-sp3;bluegene-Led07542-sp3;bluegene-Led06683-sp3;bluegene-Led09268-sp3;bluegene-Led07169-sp3;bluegene-Led00924-sp3;bluegene-Led10653-sp3;bluegene-Led03727-sp3;bluegene-Led04307-sp3;bluegene-Led09884-sp3;bluegene-Led00681-sp3;bluegene-Led00108-sp3;bluegene-Led09138-sp3;bluegene-Led04105-sp3;bluegene-Led08024-sp3;bluegene-Led05927-sp3;bluegene-Led01468-sp3;bluegene-Led00254-sp3;bluegene-Led04488-sp3;bluegene-Led05570-sp3;bluegene-Led05901-	K00459;K17818;K00008;K00128;K01613;K01886;K00480;K00101;K06966;K20247;K01455;K01183;K01178;K03948;K01950;K01187;K00852;K00254;K01613;K01915;K00480;K00306;K00558;K03146;K05349;K01694;K01178;K01115;K01687;K15634;K01785;K01874;K01613;K02564;K01679;K00480;K01580;K02144;K21196;K00128;K17066;K17066;K01427;K01480;K01514;K01110;K00463;K15371;K01176;K00720;K05349;K01426;K11262;K20246;K07512;K00928;K00020;K00968;K18369;K12350;K01796;K01113;K00480;K00147;K00953;K01915;K01230;K20838;K18083;K01426;K00122;K00737;K07748;K20246;K00102;K07542;K00432;K01736;K00480;K00817;K00318;K01443;K00128;K00065;K01477;K00993;K00799;K01657;K00029;K00211;K00757;K01176;K01649;K13501;K18097;K08967;K00766;K05917;K01497;K11426;K01613;K00766;K07511;K01580;K00451;K00621;K00877;K00899;K13830;K00059;K00801;K18551;K01626;K06210;K05857;K01739;K03844;K00698;K08678;K08764;K13501;K00706;K04486;K00698;K00222;K01228

sp3;bluegene-Led03215-sp3;bluegene-Led05188-
sp3;bluegene-Led09419-sp3;bluegene-Led11444-
sp3;bluegene-Led10119-sp3;bluegene-Led02135-
sp3;bluegene-Led01552-sp3;bluegene-Led07116-
sp3;bluegene-Led01972-sp3;bluegene-Led05785-
sp3;bluegene-Led01537-sp3;bluegene-Led07149-
sp3;bluegene-Led05563-sp3;bluegene-Led03417-
sp3;bluegene-Led07102-sp3;bluegene-Led00717-
sp3;bluegene-Led06539-sp3;bluegene-Led05523-
sp3;bluegene-Led01515-sp3;bluegene-Led01681-
sp3;bluegene-Led01165-sp3;yellowgene-Led05170-
sp3;yellowgene-Led05888-sp3;yellowgene-
Led00071-sp3;yellowgene-Led11273-
sp3;yellowgene-Led04360-sp3;yellowgene-
Led08246-sp3;yellowgene-Led08563-
sp3;yellowgene-Led08569-sp3;yellowgene-
Led02036-sp3;yellowgene-Led05235-
sp3;yellowgene-Led06932-sp3;yellowgene-
Led09653-sp3;yellowgene-Led01182-
sp3;yellowgene-Led01286-sp3;yellowgene-
Led02364-sp3;yellowgene-Led01633-
sp3;yellowgene-Led05281-sp3;yellowgene-
Led03000-sp3;yellowgene-Led01774-
sp3;yellowgene-Led10676-sp3;yellowgene-
Led05781-sp3;yellowgene-Led00534-
sp3;yellowgene-Led04339-sp3;yellowgene-
Led05621-sp3;yellowgene-Led05358-
sp3;yellowgene-Led07514-sp3;yellowgene-
Led11274-sp3;yellowgene-Led05640-
sp3;yellowgene-Led00508-sp3;yellowgene-
Led08594-sp3;yellowgene-Led02336-
sp3;yellowgene-Led10083-sp3;yellowgene-
Led00779-sp3

Metabolism	Steroid biosynthesis	6	15	0.0036129	0.1240413	ko00100	bluegene-Led08897-sp3;bluegene-Led03215-sp3;yellowgene-Led04360-sp3;yellowgene-Led03000-sp3;yellowgene-Led05179-sp3;yellowgene-Led10083-sp3	K00637;K07748;K05917;K00801;K01052;K00222
Metabolism	Histidine metabolism	8	29	0.0107206	0.2317661	ko00340	bluegene-Led03563-sp3;bluegene-Led01865-sp3;bluegene-Led03564-sp3;bluegene-Led06683-sp3;bluegene-Led05188-sp3;bluegene-Led07116-sp3;bluegene-Led01537-sp3;yellowgene-Led08594-sp3	K00128;K20247;K00128;K20246;K20246;K00817;K00128;K04486
Metabolism	Phosphonate and phosphinate metabolism	3	5	0.0112508	0.2317661	ko00440	bluegene-Led05872-sp3;bluegene-Led10653-sp3;bluegene-Led03417-sp3	K21196;K00968;K00993
Metabolism	Nitrogen metabolism	5	15	0.0186933	0.320902	ko00910	bluegene-Led04826-sp3;bluegene-Led01020-sp3;bluegene-Led00283-sp3;bluegene-Led06807-sp3;bluegene-Led08024-sp3	K00459;K01455;K01915;K15371;K01915

Metabolism	Biosynthesis of amino acids	20	115	0.0235361	0.346317	ko01230	bluegene-Led00283-sp3;bluegene-Led00242-sp3;bluegene-Led05522-sp3;bluegene-Led02641-sp3;bluegene-Led07169-sp3;bluegene-Led09138-sp3;bluegene-Led08024-sp3;bluegene-Led02135-sp3;bluegene-Led07116-sp3;bluegene-Led00717-sp3;bluegene-Led05523-sp3;bluegene-Led01165-sp3;yellowgene-Led05170-sp3;yellowgene-Led11273-sp3;yellowgene-Led02036-sp3;yellowgene-Led01633-sp3;yellowgene-Led10676-sp3;yellowgene-Led04339-sp3;yellowgene-Led05640-sp3;yellowgene-Led08594-sp3	K01915;K01694;K01687;K15634;K00928;K00147;K01915;K01736;K00817;K01657;K00211;K01649;K13501;K00766;K00766;K13830;K01626;K01739;K13501;K04486
Metabolism	Arginine and proline metabolism	8	35	0.0328432	0.4228562	ko00330	bluegene-Led03563-sp3;bluegene-Led03564-sp3;bluegene-Led03549-sp3;bluegene-Led02084-sp3;bluegene-Led09138-sp3;bluegene-Led04488-sp3;bluegene-Led01972-sp3;bluegene-Led01537-sp3	K00128;K00128;K01480;K01426;K00147;K01426;K00318;K00128

Metabolism	beta-Alanine metabolism	6	24	0.0415357	0.475353	ko00410	bluegene-Led03563-sp3;bluegene-Led00741-sp3;bluegene-Led03564-sp3;bluegene-Led01537-sp3;yellowgene-Led05235-sp3;yellowgene-Led06932-sp3	K00128;K01580;K00128;K00128;K07511;K01580
Cellular Processes	Mitophagy - yeast	7	34	0.0732351	0.5801491	ko04139	bluegene-Led00777-sp3;bluegene-Led03258-sp3;bluegene-Led05903-sp3;bluegene-Led05581-sp3;yellowgene-Led02394-sp3;yellowgene-Led07855-sp3;yellowgene-Led09032-sp3	K08341;K04441;K11644;K11227;K11644;K03115;K07203
Metabolism	Butanoate metabolism	4	15	0.0745412	0.5801491	ko00650	bluegene-Led00741-sp3;bluegene-Led03727-sp3;yellowgene-Led05235-sp3;yellowgene-Led06932-sp3	K01580;K18369;K07511;K01580
Environmental Information Processing	MAPK signaling pathway - yeast	14	85	0.0785848	0.5801491	ko04011	bluegene-Led03469-sp3;bluegene-Led02165-sp3;bluegene-Led10012-sp3;bluegene-Led03652-sp3;bluegene-Led03258-sp3;bluegene-Led06250-sp3;bluegene-Led05581-sp3;bluegene-Led03820-sp3;bluegene-Led05137-sp3;bluegene-Led02367-sp3;bluegene-Led10528-sp3;yellowgene-Led00508-sp3;yellowgene-Led04242-sp3;yellowgene-Led00465-sp3	K19860;K19806;K19842;K04371;K04441;K11232;K11227;K04627;K11215;K11246;K04563;K00706;K06666;K11228

Metabolism	Other glycan degradation	3	10	0.0889718	0.5801491	ko00511	bluegene-Led08107-sp3;bluegene-Led01656-sp3;yellowgene-Led00881-sp3	K01192;K15923;K01191
Genetic Information Processing	Non-homologous end-joining	3	10	0.0889718	0.5801491	ko03450	bluegene-Led06361-sp3;yellowgene-Led02088-sp3;yellowgene-Led09267-sp3	K10866;K10885;K10777
Metabolism	Arginine biosynthesis	4	16	0.0911211	0.5801491	ko00220	bluegene-Led00283-sp3;bluegene-Led09684-sp3;bluegene-Led06807-sp3;bluegene-Led08024-sp3	K01915;K01427;K15371;K01915
Metabolism	Tryptophan metabolism	8	43	0.0940599	0.5801491	ko00380	bluegene-Led03563-sp3;bluegene-Led03564-sp3;bluegene-Led03809-sp3;bluegene-Led02084-sp3;bluegene-Led04488-sp3;bluegene-Led01537-sp3;yellowgene-Led05235-sp3;yellowgene-Led03617-sp3	K00128;K00128;K00463;K01426;K01426;K00128;K07511;K14338
Metabolism	Glycerophospholipid metabolism	8	44	0.1045589	0.5801491	ko00564	bluegene-Led09414-sp3;bluegene-Led08479-sp3;bluegene-Led06571-sp3;bluegene-Led11358-sp3;bluegene-Led10653-sp3;bluegene-Led03417-sp3;yellowgene-Led05060-sp3;yellowgene-Led08569-sp3	K01613;K01613;K01115;K01613;K00968;K00993;K13621;K01613
Metabolism	Sesquiterpenoid and triterpenoid biosynthesis	1	1	0.1105187	0.5801491	ko00909	yellowgene-Led03000-sp3	K00801

Metabolism	Pyruvate metabolism	9	54	0.1341332	0.5801491	ko00620	bluegene-Led03563-sp3;bluegene-Led04609-sp3;bluegene-Led09307-sp3;bluegene-Led03564-sp3;bluegene-Led07542-sp3;bluegene-Led09419-sp3;bluegene-Led01537-sp3;bluegene-Led06539-sp3;bluegene-Led01165-sp3	K00128;K00101;K01679;K00128;K11262;K00102;K00128;K00029;K01649
Metabolism	Fatty acid elongation	2	6	0.1353202	0.5801491	ko00062	bluegene-Led09268-sp3;yellowgene-Led05235-sp3	K07512;K07511
Metabolism	Thiamine metabolism	2	6	0.1353202	0.5801491	ko00730	bluegene-Led06378-sp3;yellowgene-Led01286-sp3	K03146;K00877
Metabolism	Fatty acid biosynthesis	3	12	0.1385869	0.5801491	ko00061	bluegene-Led07542-sp3;bluegene-Led09268-sp3;yellowgene-Led05281-sp3	K11262;K07512;K00059
Metabolism	Taurine and hypotaurine metabolism	3	12	0.1385869	0.5801491	ko00430	bluegene-Led00741-sp3;bluegene-Led06807-sp3;yellowgene-Led06932-sp3	K01580;K15371;K01580
Metabolism	Starch and sucrose metabolism	8	47	0.1397566	0.5801491	ko00500	bluegene-Led05184-sp3;bluegene-Led01439-sp3;bluegene-Led00805-sp3;bluegene-Led06651-sp3;bluegene-Led07154-sp3;bluegene-Led09673-sp3;bluegene-Led01681-sp3;yellowgene-Led00508-sp3	K01178;K01187;K05349;K01178;K01176;K05349;K01176;K00706

Metabolism	Biosynthesis of secondary metabolites	46	359	0.1408129	0.5801491	ko01110	bluegene-Led03563-sp3;bluegene-Led09414-sp3;bluegene-Led01439-sp3;bluegene-Led08479-sp3;bluegene-Led00805-sp3;bluegene-Led00242-sp3;bluegene-Led06571-sp3;bluegene-Led05522-sp3;bluegene-Led02641-sp3;bluegene-Led10332-sp3;bluegene-Led11358-sp3;bluegene-Led09307-sp3;bluegene-Led00741-sp3;bluegene-Led03564-sp3;bluegene-Led07154-sp3;bluegene-Led09673-sp3;bluegene-Led07542-sp3;bluegene-Led07169-sp3;bluegene-Led09138-sp3;bluegene-Led04105-sp3;bluegene-Led03215-sp3;bluegene-Led02135-sp3;bluegene-Led07116-sp3;bluegene-Led01972-sp3;bluegene-Led01537-sp3;bluegene-Led03417-sp3;bluegene-Led00717-sp3;bluegene-Led05523-sp3;bluegene-Led01681-sp3;bluegene-Led01165-sp3;yellowgene-Led05170-sp3;yellowgene-Led11273-sp3;yellowgene-Led04360-sp3;yellowgene-Led08246-sp3;yellowgene-Led08569-sp3;yellowgene-Led02036-sp3;yellowgene-Led05235-sp3;yellowgene-Led06932-sp3;yellowgene-Led01633-sp3;yellowgene-Led05281-sp3;yellowgene-Led03000-sp3;yellowgene-Led10676-sp3;yellowgene-Led04339-sp3;yellowgene-Led05640-sp3;yellowgene-Led08594-sp3;yellowgene-Led10083-sp3	K00128;K01613;K01187;K01613;K05349;K01694;K01115;K01687;K15634;K01785;K01613;K01679;K01580;K00128;K01176;K05349;K11262;K00928;K00147;K00953;K07748;K01736;K00817;K00318;K00128;K00993;K01657;K00211;K01176;K01649;K13501;K00766;K05917;K01497;K01613;K00766;K07511;K01580;K13830;K00059;K00801;K01626;K01739;K13501;K04486;K00222

Metabolism	Alanine, aspartate and glutamate metabolism	5	27	0.1703684	0.6253595	ko00250	bluegene-Led00283-sp3;bluegene-Led00741-sp3;bluegene-Led06807-sp3;bluegene-Led08024-sp3;yellowgene-Led06932-sp3	K01915;K01580;K15371;K01915;K01580
Environmental Information Processing	ABC transporters	2	7	0.1761385	0.6253595	ko02010	bluegene-Led06953-sp3;yellowgene-Led06409-sp3	K15628;K05658
Metabolism	Aflatoxin biosynthesis	1	2	0.2088706	0.6253595	ko00254	bluegene-Led07542-sp3	K11262
Metabolism	Carbapenem biosynthesis	1	2	0.2088706	0.6253595	ko00332	bluegene-Led09138-sp3	K00147
Metabolism	Lysine degradation	6	37	0.2184713	0.6253595	ko00310	bluegene-Led03563-sp3;bluegene-Led05131-sp3;bluegene-Led03564-sp3;bluegene-Led01537-sp3;yellowgene-Led08563-sp3;yellowgene-Led05235-sp3	K00128;K00306;K00128;K00128;K11426;K07511
Metabolism	Riboflavin metabolism	2	8	0.2185251	0.6253595	ko00740	bluegene-Led04105-sp3;yellowgene-Led08246-sp3	K00953;K01497
Metabolism	Methane metabolism	4	22	0.2195608	0.6253595	ko00680	bluegene-Led02641-sp3;bluegene-Led04605-sp3;bluegene-Led03479-sp3;bluegene-Led05570-sp3	K15634;K17066;K17066;K00122
Cellular Processes	Autophagy - other	4	22	0.2195608	0.6253595	ko04136	bluegene-Led00777-sp3;bluegene-Led00427-sp3;bluegene-Led05503-sp3;yellowgene-Led09032-sp3	K08341;K17906;K08334;K07203
Metabolism	Ascorbate and aldarate metabolism	3	15	0.2254547	0.6253595	ko00053	bluegene-Led03563-sp3;bluegene-Led03564-sp3;bluegene-Led01537-sp3	K00128;K00128;K00128

Metabolism	Folate biosynthesis	3	15	0.2254547	0.6253595	ko00790	bluegene-Led05615-sp3;bluegene-Led00681-sp3;yellowgene-Led08246-sp3	K06897;K01113;K01497
Metabolism	Biosynthesis of various plant secondary metabolites	3	15	0.2254547	0.6253595	ko00999	bluegene-Led00805-sp3;bluegene-Led09673-sp3;yellowgene-Led01633-sp3	K05349;K05349;K13830
Cellular Processes	Autophagy - yeast	10	70	0.2390583	0.6253595	ko04138	bluegene-Led01833-sp3;bluegene-Led07671-sp3;bluegene-Led00777-sp3;bluegene-Led00427-sp3;bluegene-Led00254-sp3;bluegene-Led02642-sp3;bluegene-Led05503-sp3;yellowgene-Led06737-sp3;yellowgene-Led09032-sp3;yellowgene-Led08814-sp3	K12761;K19800;K08341;K17906;K18083;K20181;K08334;K20184;K07203;K08493
Metabolism	Fatty acid degradation	5	31	0.2529572	0.6253595	ko00071	bluegene-Led03563-sp3;bluegene-Led03564-sp3;bluegene-Led01537-sp3;yellowgene-Led05235-sp3;yellowgene-Led03617-sp3	K00128;K00128;K00128;K07511;K14338
Metabolism	Phenylalanine metabolism	3	16	0.2564547	0.6253595	ko00360	bluegene-Led02084-sp3;bluegene-Led04488-sp3;bluegene-Led07116-sp3	K01426;K01426;K00817
Metabolism	Nicotinate and nicotinamide metabolism	3	16	0.2564547	0.6253595	ko00760	bluegene-Led09363-sp3;yellowgene-Led01774-sp3;yellowgene-Led05781-sp3	K01950;K18551;K06210
